# Seroprevalence estimates for toxocariasis in people worldwide: A systematic review and meta-analysis

**DOI:** 10.1371/journal.pntd.0007809

**Published:** 2019-12-19

**Authors:** Ali Rostami, Seyed Mohammad Riahi, Celia V. Holland, Ali Taghipour, Mohsen Khalili-Fomeshi, Yadolah Fakhri, Vahid Fallah Omrani, Peter J. Hotez, Robin B. Gasser

**Affiliations:** 1 Infectious Diseases and Tropical Medicine Research Center, Health Research Institute, Babol University of Medical Sciences, Babol, Iran; 2 Immunoregulation Research Center, Health Research Institute, Babol University of Medical Sciences, Babol, Iran; 3 Cardiovascular Disease Research Center, Department of Epidemiology and Biostatistics, Faculty of Health, Birjand University of Medical Sciences, Birjand, Iran; 4 Department of Zoology, School of Natural Sciences, Trinity College Dublin, Dublin, Ireland; 5 Department of Parasitology, Faculty of Medical Sciences, Tarbiat Modares University, Tehran, Iran; 6 Student Research Committee, Babol University of Medical Sciences, Babol, Iran; 7 Department of Environmental Health Engineering, School of Public Health and Safety, Student Research Committee, Shahid Beheshti University of Medical Sciences, Tehran, Iran; 8 Cellular and Molecular Biology Research Center, Shahid Beheshti University of Medical Sciences, Tehran, Iran; 9 Texas Children’s Hospital Center for Vaccine Development, National School of Tropical Medicine, Department of Pediatrics, Baylor College of Medicine, Houston, Texas, United States of America; 10 Department of Biology, Baylor University, Waco, Texas, United States of America; 11 Department of Veterinary Biosciences, Melbourne Veterinary School, The University of Melbourne, Parkville, VIC, Australia; University of Catania, ITALY

## Abstract

**Background:**

Human toxocariasis is an important neglected disease. We performed a systematic review and meta-analysis study to estimate the global and regional prevalence of anti-*Toxocara* serum antibodies (referred to as ‘*T-*seroprevalence’) in human populations around the world.

**Methods:**

We searched five international databases (PubMed, EMBASE, Web of Science, SciELO and Scopus) for seroprevalence studies published from 1 January 1980 to 15 March 2019. We used random effect models to calculate the overall *T*-seroprevalence (with 95% CIs) in all six WHO regions and worldwide. We also conducted subgroup and linear meta-regression analyses to evaluate the impact of socio-demographic, geographical and climatic parameters on seroprevalence.

**Results:**

We identified 250 eligible studies (253 datasets) comprising 265,327 participants in 71 countries for inclusion in the present meta-analysis. The estimated global *T*-seroprevalence rate was 19.0% (95%CI, 16.6–21.4%; 62,927/265,327); seroprevalence was highest in the African region (37.7%; 25.7–50.6%) and lowest in the Eastern Mediterranean region (8.2%; 5.1–12.0%). The pooled seroprevalence for other WHO regions was 34.1% (20.2–49.4%) in the South-East Asia; 24.2% (16.0–33.5%) in the Western Pacific; 22.8% (19.7–26.0%) in the American; and 10.5% (8.5–12.8%) in the European regions. A significantly higher *T*-seroprevalence was associated with a lower income level; lower human development index (HDI); lower latitude; higher humidity; higher temperature; and higher precipitation (*P*-value < 0.001). Potential risk factors associated with seropositivity to *Toxocara* included male gender; living in a rural area; young age; close contact with dogs, cats or soil; consumption of raw meat; and the drinking of untreated water.

**Conclusions:**

The present findings indicate high levels of infection with, or exposure to *Toxocara* spp. in many countries, which calls for increased attention to human toxocariasis and improved measures to prevent adverse health risks of this disease.

## Author summary

There have been many epidemiological studies on the seroprevalence of *Toxocara* infection or toxocariasis in humans in individual countries, but there has been no systematic review/meta-analysis to estimate the extent of *Toxocara* infection or exposure in human populations worldwide. By reviewing 250 studies of 265,327 people from 71 countries, we provide the first comprehensive global estimates of *T*-seroprevalence in humans to date. The findings indicate that almost one fifth (19%) of the world’s human population is seropositive to *Toxocara*. The highest seroprevalence rates were found in Africa (37.7%) and the lowest in the Eastern Mediterranean region (8.2%). Individual countries with high HDIs and high income levels usually had low *T*-seroprevalence rates. Our findings also showed that *T*-seroprevalence varies markedly among countries, according to geographical location (coordinates) and climatic features. Evidence from this study indicates that human toxocariasis is a prevalent, neglected tropical disease and potentially one of major importance to global public health. Our study emphasizes the need for governmental authorities to pay increased attention to the risks and socioeconomic impact of HT in many countries around the world, and for them to design and implement HT prevention or control programs, considering risk predictors identified here.

## Introduction

Human toxocariasis (HT) is a neglected zoonosis with a worldwide distribution [1]. It is mainly caused by larvae of the *Toxocara canis* or *Toxocara cati*, which are intestinal ascarid nematodes of canids and felids, respectively [2]. *Toxocara* spp. have a faecal-oral transmission route, and human infection occurs following the ingestion of *Toxocara* eggs from contaminated raw vegetables [3], from contaminated soil (in gardens, sandpits and playgrounds) [4] and from larvae in undercooked or raw meats from paratenic hosts [5], and possibly through direct contact with pets [6, 7]. HT is associated with several clinical syndromes, including visceral larva migrans (VLM), ocular larva migrans (OLM), covert/common toxocariasis (CT), and can precipitate neurological and psychiatric or cardiac, allergic skin disorders and/or asthma [8–13]. Additional studies indicate that CT may represent a major cause of lung dysfunction, cognitive disturbances and intellectual deficits in children living in poverty [14–17]. Nevertheless, most infections remain undiagnosed due to the asymptomatic, mild or non-specific clinical nature of infection(s) [18].

The diagnosis of HT can be made using conventional methods, including blood tests (blood count and eosinophilia) and histopathological examination, or molecular techniques usually based on the polymerase chain reaction (PCR) to identify larval DNA in tissue or body fluid samples. In most epidemiological studies, however, serological methods, such as enzyme-linked immunosorbent assays (ELISAs) and/or Western blot analysis using *Toxocara* spp. excretory-secretory antigens (TES) are mostly employed [1, 19]. The diagnostic sensitivity and specificity of these methods can vary, and depend on the antigens (e.g., crude larval, native or recombinant TES, or glycan or deglycosylated TES antigens), antibody conjugates (affinity-purified or not), their dilutions and the blockers utilised, the antibodies being detected (e.g., total IgG, IgG subclass, or IgM) and the extent of assay optimization [1, 20–22].

Despite the many epidemiological surveys in different countries [1], there is limited knowledge of the seroprevalence of HT and *Toxocara* infections in humans and other animals at the global, country and regional levels. There is also often limited clinical awareness about the adverse effects of HT, and a lack of information or databases on the efficacy of clinical, treatment and management, even in countries with high HT burdens [23], and there are currently no reported estimates for HT in any of the Global Burden of Disease (GBD) studies. However, global and regional estimates of prevalence of anti-*Toxocara* serum antibodies (referred to henceforth as “*T-*seroprevalence”) in human populations can increase the awareness of health-care policymakers about the global burden of infections, disease or exposure and, consequently, lead to a prioritization of cost-effective screening and intervention(s) for HT. For example, because HT can be treated with albendazole and other anthelminthic drugs, there is potential for adding HT to World Health Organization (WHO) programs of preventive chemotherapy, which currently target other helminthiases. In this paper, we conducted a comprehensive systematic review and meta-analysis to estimate the *T-*seroprevalence worldwide in the healthy human population. We also evaluated the impact of geographical, climatic and socio-demographic factors on this *T-*seroprevalence in different countries and regions.

## Methods

This study followed the Preferred Reporting Items for Systematic Reviews and Meta-analyses guidelines [24]. The protocol for this meta-analysis is published and accessible in PROSPERO (CRD42018118172).

### Search strategy and selection criteria

To identify all relevant papers pertaining to *T-*seroprevalence data in the healthy population, two independent authors (A.R. and A.T.) performed an extensive search of the PubMed/MEDLINE, Web of Science, EMBASE, Scopus and SciELO databases, for the period from 1 January 1980 to 15 March 2019. No geographic or language restrictions were applied. Google Translate was used for studies published in languages other than English. The “healthy population” refers to healthy people without known disease, infection or exposure to *Toxocara* spp. (e.g., without eosinophilia, allergy, ocular or neurological disorders) who do *not* have an occupation (e.g., gardener, waste collector or veterinarian) with a high risk of exposure to *Toxocara* spp. A *T-*seroprevalence in the healthy population was considered as the main outcome of interest. *T-*seroprevalence was defined as the number of people test-positive for anti-*Toxocara* IgG serum antibody divided by the total number of people in the population screened using one or more serological methods. The performance (diagnostic sensitivity and specificity) of in-house or commercial serological and/or Western blot assays were not assessed, because this information was usually not described or adequately described in published studies, but was known to vary, sometimes quite considerably, among investigations. We accepted individual authors’ definitions (criteria value) of cut-off values for test-positivity in diagnostic methods in published studies.

A combination of the following search terms using the Boolean operators “OR” and/or “AND” were used in the literature searches: “*Toxocara* infection”, “*Toxocara* spp.”, “*Toxocara canis*”, “*Toxocara cati*”, “toxocariasis”, “seroprevalence”, “seroepidemiology”, “prevalence”, and “risk factor”. We also manually scanned the reference lists of all articles collected (See S1 Text: Supplementary file for the details of the databases searches). When required, corresponding authors were contacted for additional data or information as required.

After deleting duplicates, the titles, abstracts and/or entire articles were screened for relevance by two authors (VFO and MKF) and any discrepancy was resolved by a third author (AR). Inclusion criteria were: (i) original peer-reviewed studies reporting the *T-*seroprevalence in the healthy population; (ii) studies in which at least 50 participants were recruited; (iii) studies that used methods for specific anti-*Toxocara* serum antibody detection; and (iv) in case-control studies, data were collected only for healthy people (i.e. controls).

Articles that did not meet the four criteria (i-iv) were excluded. Other exclusions were studies conducted on people (populations) at high risk of acquiring HT (e.g., patients with ocular, allergic, or neurological disorders, patients with eosinophilia, mentally retarded patients, psychiatric patients, gardeners, hunters, veterinarians and/or waste collectors), investigations of animals other than humans, and those with overlapping participation in multiple studies (in such cases, only the study with the higher sample size was included), case-reports or case-series studies, studies that included patients proven to be *Toxocara*-infected at the baseline, diagnostic studies, or reviews, systematic reviews or meta-analyses.

### Data extraction and quality assessment

After reviewing eligibility criteria, information from eligible studies was extracted independently and in duplicate by two authors (AT and MKF) into a Microsoft Excel spreadsheet (2016 version; Microsoft, Redmond, WA, USA). In cases of inconsistencies, a third reviewer (AR) was consulted, and a decision was made by consensus. The following information was recorded: first author’s last name, year of publication, years of study implementation, country, city, type of study (cross-sectional or case-control), type of diagnostic method used (ELISA and/or Western blot), type of population (children [≤ 18 years of age], adults [≥ 19 years of age], or both), sample size and number of seropositive people. Moreover, we extracted data on sample size in distinct age groups (≤ 10, 11–20, 21–40, 41–60, ≥ 61 years), if available.

For each study area, we recorded data on the corresponding World Hemisphere, WHO-defined regions (Africa [AFR], Eastern Mediterranean [EMR], Europe [EUR], Southeast Asia [SEAR], the Americas [AMR], and Western Pacific [WPR]), WHO-defined sub-regions based on mortality strata (A to E) [25, 26], income and human development index [HDI] levels of each country studied, geographical latitude and longitude, relative humidity, mean temperature and precipitation rate. Information on sources of income and HDI levels were taken from the World Bank Group and the United Nations Development Program [27, 28]. The data sources for geographical and climatic status were: https://www.timeanddate.com/, https://en.climate-data.org/ and https://gps-coordinates.org/.

### Meta-analysis

The pooled seroprevalence estimates were calculated using a random effects model (REM) at a 95% confidence interval (CI) [29], to give a conservative estimate of the worldwide *T-*seroprevalence in the healthy population. The seroprevalence estimates were stratified according to World Hemispheres, WHO-defined regions and sub-regions. Furthermore, for individual countries with two or more eligible studies, we synthesised a country-specific seroprevalence rate employing REM. To estimate the number of people exposed to or infected with *Toxocara*, we extrapolated our seroprevalence estimates to the total population living in WHO-defined regions in 2016, perusing the WHO database [30]. We assumed that countries with data missing for a particular region had comparable *T-*seroprevalence to our pooled mean *T-*seroprevalence. Heterogeneity among studies was calculated and the Cochran Q-test, with a *P* value of < 0.05 and an *I*^2^ statistic with a cut-off of 50% [31], were used to define a statistically significant degree of heterogeneity. We did not assess publication bias, as it is not relevant for prevalence studies [32].

We undertook subgroup and meta-regression analyses to explore the potential drivers of heterogeneity. Subgroup analyses were executed for the following variables (1) study characteristics (type of participants, type of diagnostic method, type of study design, sample size, years of study implementation); and (2) socio-demographic, geographical and climate variables (country’s the income level, country’s HDI level, country’s latitude, longitude, relative humidity, mean temperature and precipitation rate). Meta-regression analyses were performed on the following parameters: years of study implementation, and country’s income level, country’s HDI level, country’s latitude, longitude, relative humidity, mean temperature, precipitation rate and country’s overall contamination rate of *Toxocara* spp. eggs in soil [available from a previous study]) [4].

In order to identify the potential risk factors related to HT seroprevalence, we assessed various risk factors by determining an odds ratio (OR) and the related 95% CI in 2*2 tables. Risk factors examined in this study included sex (male or female), residence (rural or urban), age (≤ 10, 11–20, 21–40, 41–60, ≥ 61 years), close contact with dogs and cats (Yes/No), contact with soil (Yes/No), consumption of raw/unwashed vegetables (Yes/No), consumption of raw/undercooked meat (Yes/No) and drinking untreated or unfiltered water (Yes/No). Publication-bias was measured by Egger’s regression asymmetry test (quantitative method) for these analyses. All statistical analyses were conducted using STATA v.13 (STATA Corp., College Station, Texas, USA). The results from statistical analyses were significant if the *P* value was < 0.05.

## Results

### Study characteristics

**Fig 1:**
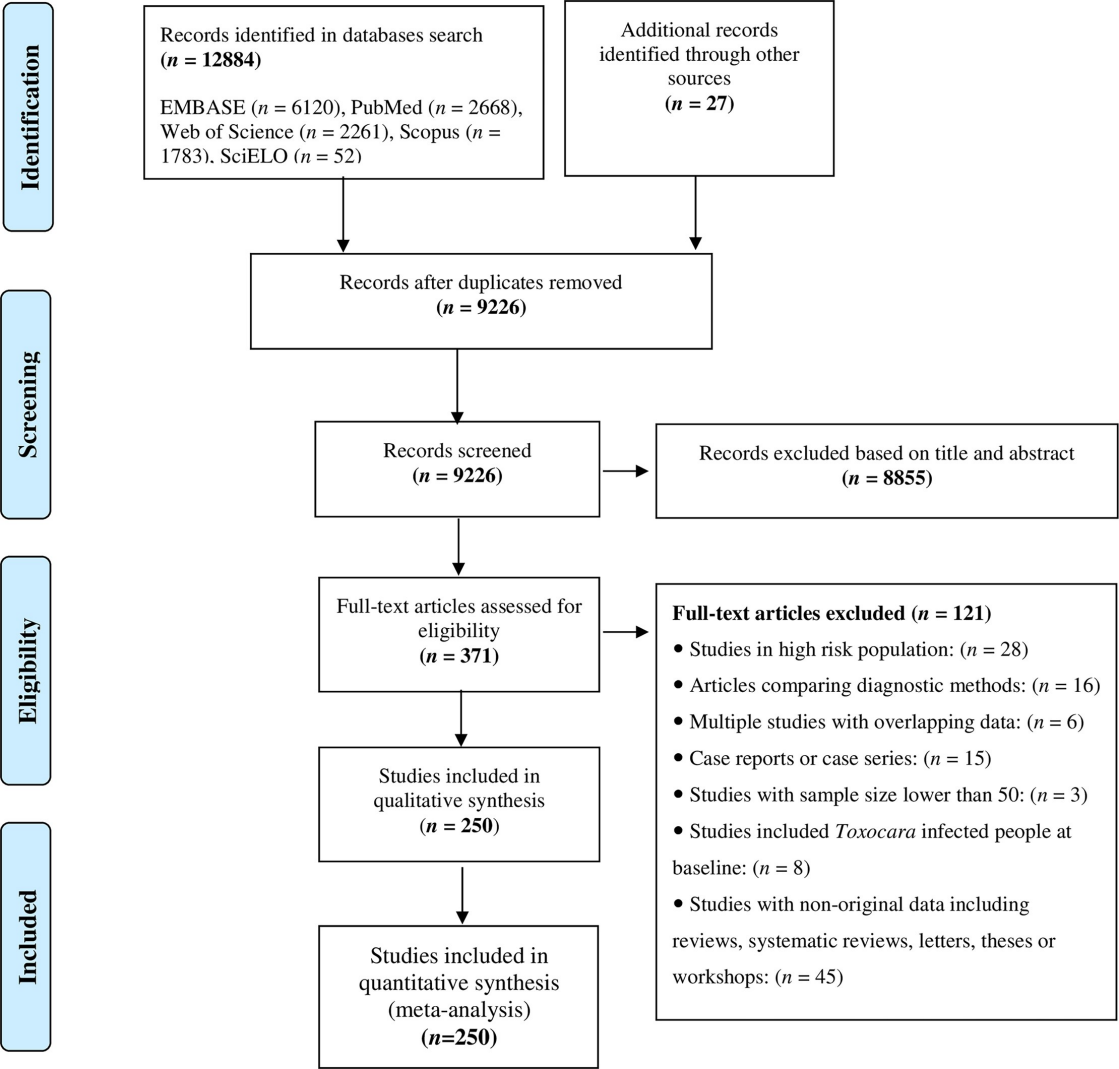
PRISMA flowchart, showing the search and study selection strategy.

The initial literature search revealed 12,911 articles, 371 of which were inferred to be relevant to the study topic (Fig 1). After applying inclusion and exclusion criteria, 250 articles containing 253 datasets from 71 countries fulfilled the eligibility criteria and were retained for data extraction. These 253 datasets represented 265,327 individual people (participants) and covered all six WHO-regions; 104 datasets were available for the Americas (87,173 participants), 64 for Europe (67,610 participants), 30 for the Eastern Mediterranean region (11,095 participants), 24 for the Western Pacific region (89,997 participants), 19 for Africa (6,360 participants), and 12 for the South-East Asia region (3,092 participants). Of all articles included, 29 were published in languages other than English, and were from South America or Europe; most of them had an English abstract. Data were obtained for 43.3% of countries in the European region, 45.7% of countries in the region of the Americas, 25.5% of countries in the African region, 37% of countries in the Western Pacific region, 24% of countries in the Eastern Mediterranean region and 45.5% of countries in the Southeast Asian region. The countries with the highest numbers of reports were Brazil (40 studies), Iran (23 studies), Argentina (12 studies) and Canada (9 studies). Most of the studies (*n* = 216) included in the meta-analysis had a cross-sectional design, although 34 investigations had a case-control design. For these studies, we extracted data only for healthy people.

For the detection of anti-*Toxocar*a serum antibody (IgG) in individual people, ELISA was used in most studies (*n* = 240), Western blot analysis was used in ten studies and both of these techniques were used simultaneously in nine studies. For studies that employed both diagnostic methods, ELISA results were used. The recruited populations were adults for 51 datasets, children for 106 datasets and both children and adults for 93 datasets. The study references, main study characteristics, diagnostic methods, sample sizes and numbers of participants with evidence of seropositivity reported in all 250 studies are presented in S1 Table.

### Worldwide seroprevalence of toxocariasis

We estimated pooled global *T*-seroprevalence according to WHO-epidemiological regions (Table 1), which are further subdivided into sub-regions according to mortality strata (A to E). The global prevalence in the healthy population, when data for all 253 datasets representing 71 countries were pooled, was 19.0% (95%CI, 16.6–21.4%; 62,927/265,327). There was statistically significant heterogeneity among studies (*I*^2^ = 99.6%, *P* < 0.001; Table 1). The overall prevalence rates in the Eastern, Western, Northern and Southern hemispheres were 16.5% (13.3–20.0%), 22.0% (19.1–25.1%), 14.8% (12.2–17.5%) and 30.3% (25.4–35.6%), respectively (S1 Fig and S2 Fig).

**Table 1:** Global, regional and sub-regional pooled *T*-seroprevalence (results from 250 studies performed in 71 countries).

WHO-regions*/subregions#	Number of datasets	Number of seropositive samples/total number of samples	Pooled seroprevalence (%) (meta-analysis) [95% CI]	Weight (%)	Heterogeneity	
					*I*^2^ (%)	χ^2^
**Global**	**253**	**62,927/265,327**	**19.0 (16.6**–**21.4)**	**100**	**57983.1**	**99.6**
**African region**	**19**	**2,339/6,360**	**37.7 (25.7**–**50.6)**	**7.5**	**1920.3**	**99.1**
AFR-D	9	1,480/2,543	52.6 (34.7–70.2)	3.5	96.6	672.5
AFR-E	10	859/3,817	25 (17.6–33.3)	4.0	97.6	264.7
**South-East Asia region**	**12**	**1,193/3,092**	**34.1 (20.2**–**49.4)**	**4.8**	**784.0**	**98.6**
SEAR-B	6	847/2,069	39.2 (19.2–61.3)	2.4	98.9	442.1
SEAR-D	6	346/1,023	29.1 (9.6–53.8)	2.4	98.5	327.8
**Western Pacific region**	**24**	**37,662/89,997**	**24.2 (16.0**–**33.5)**	**9.5**	**6030.8**	**99.6**
WPR-A	4	70/1,143	3.7 (1.5–6.7)	1.6	80.2	15.2
WPR-B	20	37,592/88,584	29.9 (20.8–40)	7.9	99.6	4712.9
**Region of the Americas**	**104**	**13,524/87,173**	**22.8 (19.7**–**26.0)**	**41.1**	**11780.8**	**99.1**
AMR-A	21	5,947/60,614	9.6 (6.9–12.7)	8.1	99.2	2454.5
AMR-B	74	6,818/23,300	26.8 (22.2–31.6)	29.2	98.5	4847.8
AMR-D	9	759/3,259	27.3 (17.3–38.6)	3.6	97.8	360.4
**European region**	**64**	**7,024/67,610**	**10.5 (8.5**–**12.8)**	**25.2**	**4861.6**	**98.7**
EUR-A	38	3,759/45,497	9.8 (7.3–12.6)	15.1	98.7	2950.5
EUR-B	21	2,105/14,987	12.4 (8.8–16.5)	8.2	97.7	851.2
**EUR-C**	5	1,160/7,126	9.4 (4.1–16.5)	2.0	98.4	249.8
**Eastern Mediterranean region**	**30**	**1,185/11,095**	**8.2 (5.1**–**12.0)**	**11.8**	**1202.4**	**97.6**
EMR-B	24	1,034/10,141	6.5 (3.6–10)	9.5	97.6	976.9
EMR-D	6	151/954	18.1 (4.4–38)	2.3	97.6	208.2

*WHO regions are sorted according to prevalence rates.

#Subregions are sorted based on mortality strata (A to E). A, very low child mortality and low adult mortality; B, A, low child mortality and low adult mortality; C, low child mortality and high adult mortality; D, high child mortality and high adult mortality; E, high child mortality and very high adult mortality. The developed countries are at (AMR-A, EUR-A, EUR-B, EUR-C and WPR-A), low-mortality developing countries are at (AMR-B, EMR-B, SEAR-B and WPR-B), and high-mortality developing countries are at (AFR-D, AFR-E, AMR-D, EMR-D and SEAR-D).

**Fig 2:**
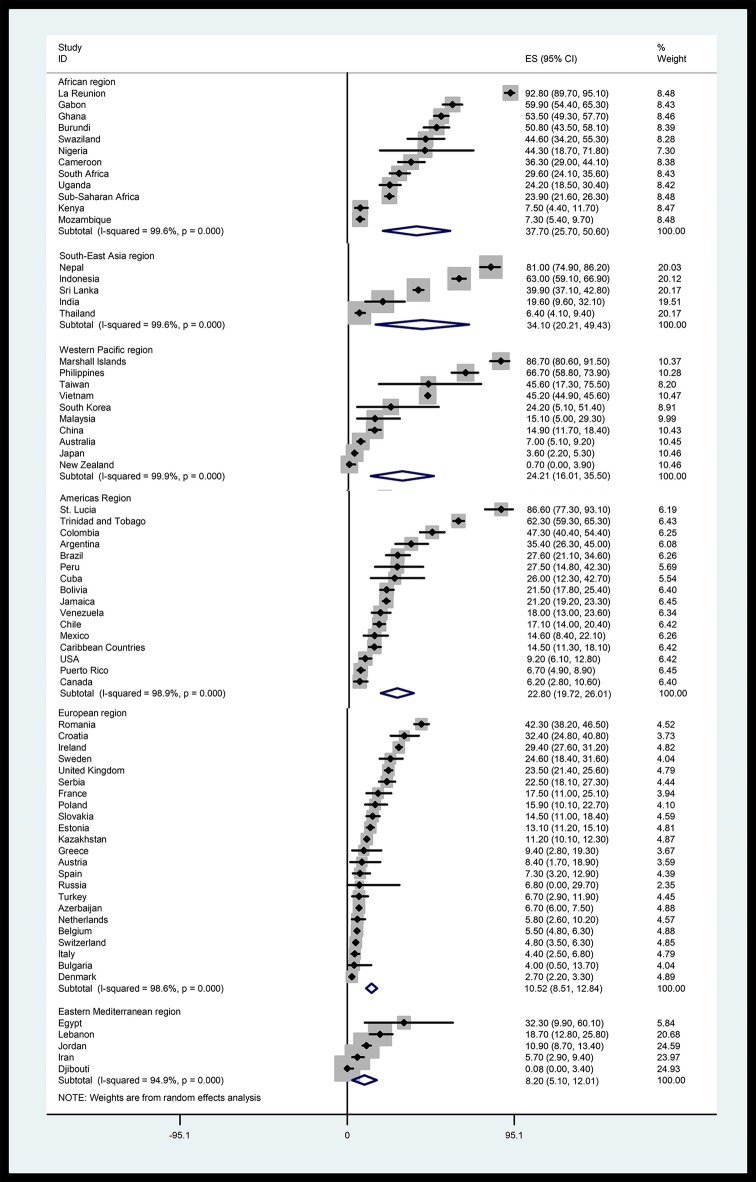
Forest plot of the *T*-seroprevalence by WHO-region and globally. ES: estimated *T*-seroprevalence for WHO-regions and individual countries.

**Fig 3:**
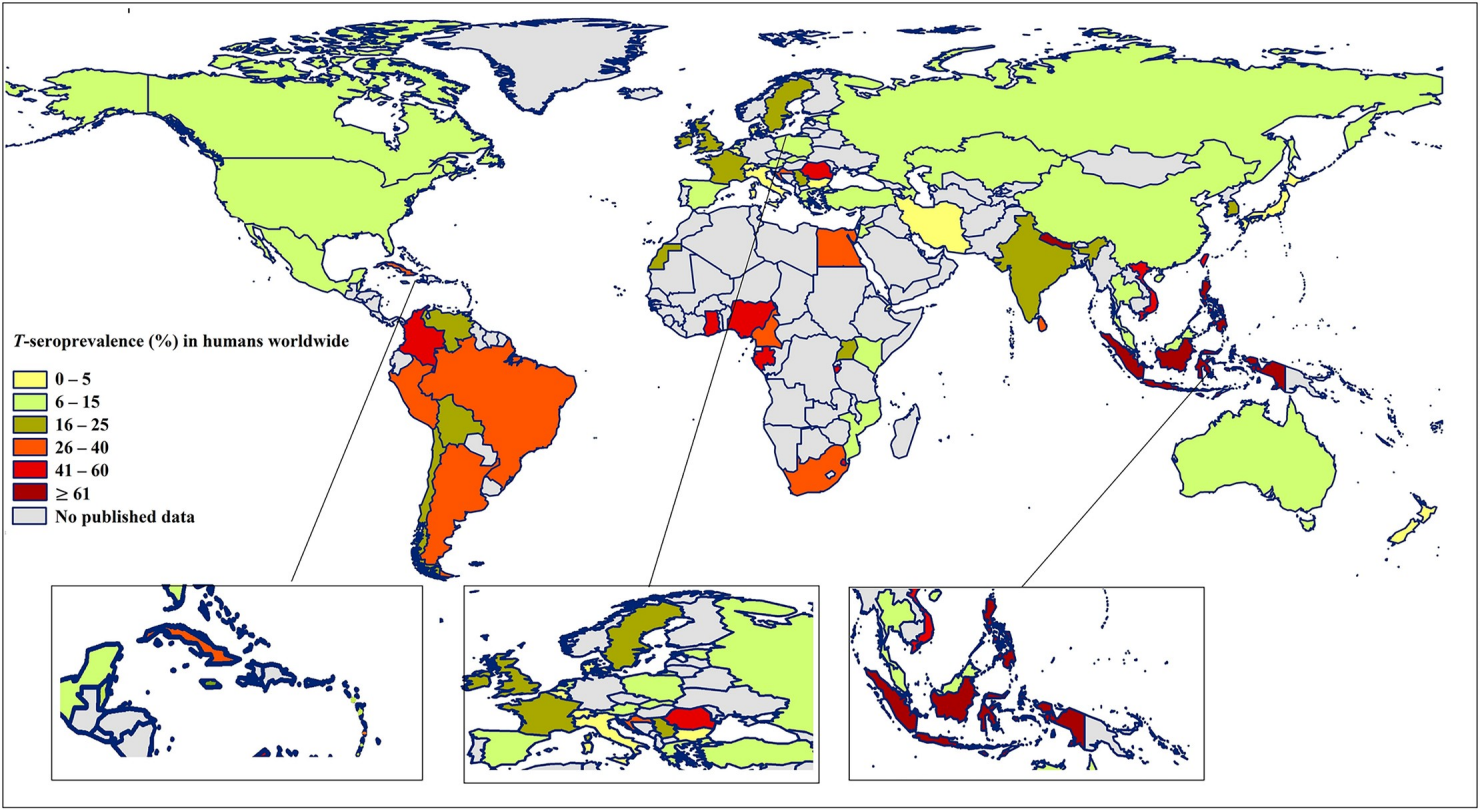
*T*-seroprevalence (%) in humans worldwide. Maps drawn by the authors; they are original and have not been published previously.

Concerning WHO-epidemiological regions, the highest seroprevalence of HT (37.7%; 25.7–50.6%) was found in the African region, and the lowest seroprevalence (8.2%; 5.1–12.0%) was found in the Eastern Mediterranean region. The pooled *T*-seroprevalence rates in other WHO regions were: 34.1% (20.2–49.4%) in the South-East Asia, 24.2% (16.0–33.5%) in the Western Pacific, 22.8% (19.7–26.0%) in the American and 10.5% (8.5–12.8%) in the European regions. Due to socio-demographic and climate differences among regions, we further divided the American region into North- and South-American countries. Based on this analysis, the overall prevalence rates were 12.8% (10.0–15.8%) in North America and 27.8% (23.1–32.7%) in South America (Fig 2 and S2 Table). With regard to WHO sub-regions based on mortality strata (A to E), the highest seroprevalence rates in the sub-regions were: 52.6% in AFR-D (34.7–70.2%) and 39.2% (19.2–61.3%) in SEAR-B, and the lowest seroprevalence rates were 3.7% (1.5–6.7%) in WPR-A, 6.5% (3.6–10%) in EMR-B, 9.4% (4.1–16.5%) in EUR-C and 9.6% (6.9–12.7%) in AMR-A. Further details regarding the *T*-seroprevalence rates in WHO-regions, sub-regions, and individual countries and results of the analyses of heterogeneity are presented in Table 1, Fig 2, Fig 3 and S2 Table. An extrapolation to the world population in 2016 estimated that 1.4 (1.2–1.5) billion individuals worldwide are infected by or exposed to *Toxocara*, or have HT, with the highest burden predicted in the South-East Asia region (664 million individuals) and lowest in Eastern Mediterranean region (54 million individuals) (Table 2). The highly populated Southeast Asian countries of Indonesia, Philippines and Vietnam exhibited some of the highest global seroprevalence rates, as did Nepal in South Asia; Gabon, Ghana, and Nigeria in West Africa; Colombia in Latin America; and Romania in Eastern Europe.

**Table 2:** The global *T*-seroprevalence and number of seropositive people in the general population in the WHO regions.

WHO-region(s)	Seroprevalence estimates (%) [95% CI]	Population size per region*	Number of seropositive people (range)
**Global (all 6 WHO regions)**	**19.0 (16.6–21.4)**	**7,430,261,000**	**1,411,749,590 (1,233,423,326–1,590,075,854)**
The Americas	22.8 (19.7–26.0)	992,155,000	226,211,340 (19,5454,535–257,960,300)
European	10.5 (8.5–12.8)	916,315,000	96,213,075 (77,886,775–117,288,320)
South-East Asia	34.1 (20.2–49.4)	1,947,632,000	664,142,512 (393,421,664–962,130,208)
African	37.7 (25.7–50.6)	1,019,922,000	384,510,594 (262,119,954–516,080,532)
Eastern Mediterranean	8.2 (5.1–12.0)	664,336,000	54,475,552 (33,881,136–79,720,320)
Western Pacific	24.2 (16.0–33.5)	1,889,901,000	457,356,042 (302,384,160–633,116,835)

* Population data according to WHO region (2016). Accessible via http://apps.who.int/gho/data/view.main.POP2020?lang=en.

### Subgroup analyses according to study characteristics

In subgroup analysis according to the type of population recruited in studies included, the global *T*-seroprevalence was 18.3% (14.5–22.5%) in studies of children, 12.8% (8.4–17.9%) in adults and 22.7% (19.1–26.5%) in both children and adults (all age groups). Considering the type of diagnostic methods used, seroprevalence rates were 18.6% (16.3–21.1%) in studies that used ELISA and 27.8% (8.7–52.5%) in those that employed Western blot analysis. Additional subgroup analyses, according to study design, study sample size and years of study-implementation, are given in S3 Table.

### Subgroup analyses based on socio-demographic, geographical and climatic parameters

We conducted subgroup analyses according to socio-demographic characteristics and different geographic or climate parameters that might affect the *T*-seroprevalence (Table 3). In subgroup analysis according to country’s income, the highest prevalence (39.4%; 20.1–60.6%) was found in countries with low income and the lowest prevalence (14.2; 12.2–16.3) was found in countries with high income levels (Table 3). In addition, the highest (36.5%; 15.0–61.4%) and lowest (13.6%; 11.7–15.6%) prevalence were found in countries with low and very high levels of HDI, respectively (Table 3). Additional subgroup analyses with respect to geographic and climate parameters (i.e. latitude, longitude, humidity, mean temperature, and precipitation) are given in Table 3.

**Table 3:** *T*-seroprevalence in the general population based on sub-groups according to different socio-demographic and geographic parameters, calculated using a random effects model (REM).

Parameters/subgroups	Number of datasets	Number of seropositive people/number of people tested	Seroprevalence (%) [95% CI]	Heterogeneity	
				χ^2^	I^2^ (%)
**Income**					
Low	9	1,254/3,824	39.4 (20.1–60.6)	1338.3	99.4
Lower middle	28	37,340/83,496	33.8 (27.1–40.9)	1780.1	98.5
Upper middle	117	11,302/56,202	18.7 (15.8–21.7)	9484.8	98.8
High	99	13,032/121,805	14.2 (12.2–16.3)	9819.5	99.0
**Human Development Index**					
Low	11	1,108/2,580	36.5 (15.0–61.4)	1598.1	99.4
Medium	25	37,103/83,884	35.0 (28.1–42.3)	1734.9	98.6
High	117	11,283/55,205	19.3 (16.2–22.7)	10974.8	98.9
Very high	100	13,433/123,658	13.6 (11.7–15.6)	9049.1	98.9
**Latitude**					
0–10°	39	4,123/12,203	33.8 (27.5–40.4)	2160.7	98.2
10–20°	33	38,969/92,528	24.6 (18.4–31.5)	5406.4	99.4
20–30°	47	4,611/29,620	26.4 (19.2–34.3)	7948.4	99.4
30–40°	70	7,863/67,634	13.4 (10.9–16.0)	5434.4	98.7
40–50°	38	4,038/35,187	10.5 (8.3–12.9)	1612.7	97.7
≥ 50°	26	3,323/28,155	10.6 (7.1–14.7)	2541.8	99.0
**Longitude**					
0–10°	30	3,757/27,487	16.0 (11.1–21.5)	3886.9	99.2
10–20°	18	1,903/24,613	13.8 (8.8–19.5)	1693.9	99.0
20–30°	15	1,127/6,354	15.3 (9.6–22.2)	662.7	97.9
30–40°	28	3,229/11,608	25.0 (19.1–31.5)	1522.2	98.2
40–50°	35	1,974/15,896	13.8 (9.6–18.6)	2118.7	98.4
50–60°	26	2,221/9,086	21.5 (12.9–31.7)	2976.0	99.2
60–70°	24	2,392/11,080	25.2 (16.3–35.2)	2990.5	99.2
70–80°	25	3,056/16,469	20.3 (15.5–25.7)	1457.5	98.4
80–90°	7	1,002/4,781	23.9 (8.2–44.5)	851.3	99.3
90–100°	7	4,029/45,477	9.8 (6.2–14.0)	875.3	99.3
100–110°	13	35,430/80,268	14.0 (5.4–25.7)	1655.2	99.3
110–120°	4	650/3,588	34.0 (9.5–64.3)	491.6	99.4
≥ 120°	21	2,127/8,620	23.8 (14.2–34.9)	2473.7	99.2
**Relative humidity (%)**					
< 40	12	399/4,934	6.6 (2.9–11.5)	409.3	97.3
40–50	13	4,092/46,628	7.3 (4.8–10.3)	999.3	98.8
50–60	22	1,007/9,036	10.2 (7.0–14.0)	535.5	96.1
60–70	48	3,940/39,168	14.6 (10.9–18.6)	4704.9	99.0
70–80	122	48,743/142,581	23.7 (20.1–27.5)	23209.5	99.5
≥ 80	36	4,746/22,980	25.9 (19.1–33.3)	5145.3	99.3
**Mean temperature (°C)**					
< 7	13	1,362/11,585	6.9 (3.1–11.8)	907.7	98.7
7.1–13	55	7,085/51,768	13.2 (10.3–16.3)	5397.2	99.0
13.1–19	90	10,152/82,673	15.9 (13.6–18.3)	6856.4	98.7
19.1–25	53	4,060/28,696	23.0 (17.4–29.0)	5589.0	99.1
25.1–30	42	40,268/90,605	35.7 (29.8–41.9)	4084.2	99.0
**Precipitation (mm)**					
0–250	24	1,721/26,619	10.8 (7.3–14.7)	1560.1	98.5
250–500	50	3,422/27,072	12.0 (9.0–15.2)	2932.8	98.3
500–1000	87	11,968/94,576	16.6 (14.2–19.1)	8132.6	98.9
1000–2000	82	44,738/114,194	27.3 (22.9–31.9)	13048.6	99.4
≥ 2000	10	1,078/2,866	38.3 (24.0–53.7)	631.0	98.6

### Meta-regressions based on socio-demographic, geographical and climatic parameters

**Fig 4:**
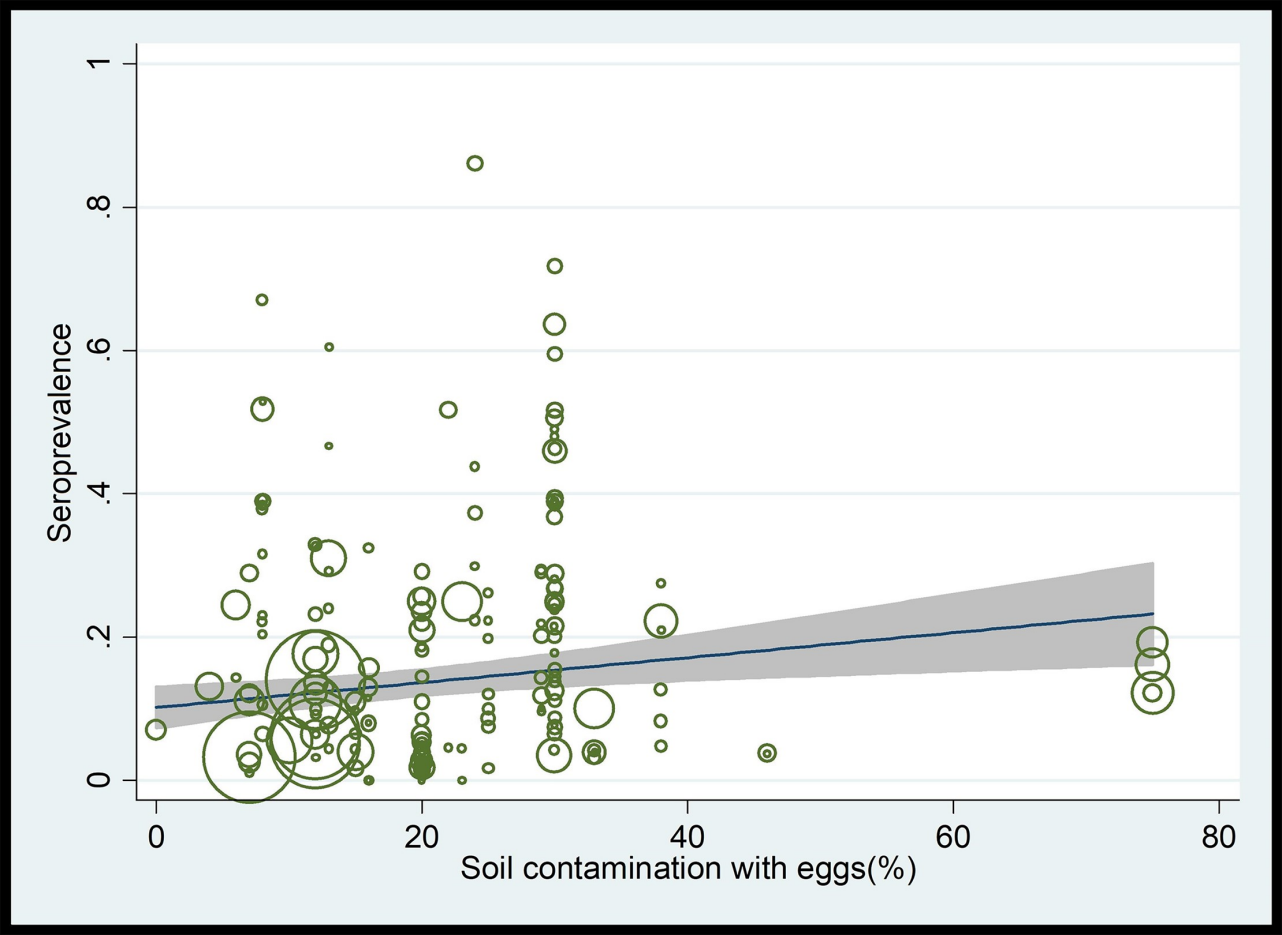
Meta-regression analysis showing a non-statistically significant upward trend in the rates of *T*-seroprevalence in humans with increasing contamination of soil with *Toxocara* eggs.

We also investigated temporal, socio-demographic, geographical and climatic effects on *T*-seroprevalence by meta-regression. Meta-regression on years of study-implementation showed a non-significant increase in prevalence over time (1980–2019) (coefficient [*C*] = 0.0007; *P*-value = 0.57). With respect socio-demographic parameters, we revealed a significant decreasing trend in prevalence with increasing income (*C* = -0.082; *P*-value < 0.001) and HDI levels (*C* = -0.084; *P*-value < 0.001) in a country. In addition, a significant decreasing trend was observed for *T*-seroprevalence with increasing geographical latitude (*C* = -0.005, *P*-value < 0.001). With regard to longitude, we observed a non-significant increasing trend in prevalence in countries with higher geographical longitudes (*C* = 0.0005, *P*-value = 0.1). We found a significant increasing prevalence with an increase in relative humidity (*C* = 0.005, *P*-value < 0.001), mean temperature (*C* = 0.01; *P*-value < 0.001) and precipitation rates (*C* = 0.0001, *P*-value < 0.001) (S3A–S3H Fig). Finally, meta-regression analyses showed a non–statistically significant increasing trend in *T*-seroprevalence rates with an increasing overall contamination rate with *Toxocara* spp. eggs in public places (*C* = 0.006, *P*-value = 0.5) (Fig 4).

### Risk factors for seropositivity

Considering the aspect of risk, our results revealed that being male (odds ratio [OR], 1.27; 95% CI, 1.17–1.39), living in rural areas (OR, 1.76; 95%CI, 1.35–2.31), being younger in age (OR, 1.89; 95%CI, 1.72–2.8), having contact with dogs (OR, 1.72; 95%CI, 1.47–2.02), contact with cats (OR, 1.61; 95%CI, 1.14–2.29), contact with soil (OR, 2.1; 95%CI, 1.59–2.79), consuming raw meat (OR, 1.59; 95%CI, 1.03–2.47) and drinking untreated water (OR, 1.97; 95%CI, 1.44–2.71) represent possible risk factors for seropositivity to *Toxocara* worldwide (Table 4). Egger’s test results, to identify the publication bias for these analyses, are present in Table 4.

**Table 4:** Risk factors associated with seropositivity to *Toxocara* in the general population in the world.

Variables (number of datasets)	Number of seropositive people/number of people screened	Seroprevalence (%) of human toxocariasis [95% CI]	Odds ratio [OR] [95% CI]	Heterogeneity		Publication bias *P*-value |t|
				χ^2^	I^2^ (%)	
**Gender (92)**						0.14
Male	20,746/66,202	25.5 (20.8–30.6)	1.27 (1.17–1.39)	14970.8	99.4	
Female	25,583/84,599	21.6 (16.9–26.8)	1	18938.0	99.5	
**Residence (25)**						0.04
Rural	1,866/8,297	20.9 (14.9–27.5)	1.76 (1.35–2.31)	1140.1	97.9	
Urban	1,251/8,272	13.1 (8.3–18.8)	1	1010.8	97.9	
**Age**						< 0.05
≤ 10 (59)	4,518/23,812	22.5 (17.1–28.4)	1.89 (1.72–2.8)	60004.3	99.0	
11–20 (31)	1,074/8,055	19.2 (13.4–25.7)	1.24 (1.11–1.39)	1229.6	97.6	
21–40 (19)	1,044/14,208	16.2 (11.2–22.0)	0.59 (0.53–0.66)	1048.6	98.3	
41–60 (15)	678/7,691	17.4 (11.5–24.2)	0.71 (0.63–0.8)	480.2	97.1	
≥ 60 (10)	521/4,731	18.1 (10.0–27.6)	1	217.0	95.9	
**Close contact with dogs (44)**						0.07
Yes	2,326/8,974	25.8 (20.5–31.5)	1.72 (1.47–2.02)	1501.5	97.1	
No	2,029/12,654	16.6 (12.6–21.1)	1	1601.4	97.3	
**Close contact with cats (16)**						0.2
Yes	518/1,851	26.8 (15.7–39.5)	1.61 (1.14–2.29)	453.4	96.7	
No	1,248/5,379	19.0 (12.2–26.7)	1	669.7	97.8	
**Contact with soil (19)**						0.45
Yes	1,770/6,274	30.4 (21.2–40.5)	2.1 (1.59–2.79)	1141.3	98.4	
No	847/6,061	17.1 (12.2–22.7)	1	477.3	96.2	
**Consumption of raw/unwashed vegetable (11)**						0.77
Yes	827/3,639	32.0 (19.0–46.5)	1.34 (0.93–1.92)	620.8	98.4	
No	610/3,287	27.6 (15.5–41.6)	1	600.4	98.3	
**Consumption of raw meat (10)**						0.22
Yes	1,099/4,297	40.0 (23.7–57.5)	1.59 (1.03–2.47)	981.9	99.1	
No	740/3,381	28.9 (19.0–39.9)	1	355.5	97.5	
**Drinking untreated water (7)**						0.17
Yes	363/892	45.9 (29.0–70.2)	1.97 (1.44–2.71)	209.8	97.1	
No	344/1,544	31.5 (21.4–42.7)	1	111.5	94.6	

## Discussion

To our knowledge, this is the first comprehensive meta-analysis to predict the global, regional and national seroprevalence rates of HT. We believe that our estimates are robust, because we have assembled data from all available studies and applied rigorous statistical methods to calculate estimates. Our results show that, globally, approximately one fifth (1.4 billion individuals) of the world’s human population is exposed to *Toxocara* and that *T*-seroprevalence varies substantially, depending on country and region. The prevalence rates estimated here could be the cause of many clinical sequelae (allergic, ocular and neurological disorders), suggesting a marked public health impact and a need to implement preventive and control strategies. However, *T*-seroprevalence rates reported here must be interpreted with caution because of possible variability in diagnostic sensitivity and specificity among different serological methods. In present study, ELISA was used in most studies included. It has been shown that differences in TES antigen preparation in studies that used in-house ELISAs and also different protocols by manufacturers to preparation of TES result in variation in the sensitivity and specificity of ELISAs used in published studies worldwide [19, 33]. Another issue that needs to be mentioned in relation to TES-ELISA is possible cross-reactivity with serum antibodies directed against other helminths, such as *Ascaris*, *Strongyloides* and/or *Trichinella* species [19, 33]. Such cross-reactivity depends on the quality of the antigen used, how well an ELISA has been standardised, and how the assay is performed (cf. [19]), and might have led to an overestimation of prevalence in some studies, particularly those conducted in geographical areas where polyparasitism in humans is prevalent.

The *T*-seroprevalence rates estimated varied among WHO-regions. The highest seroprevalence rates were found in African (37.7%) and South-East Asian countries (34.1%). For example, the highly populated Southeast Asian countries of Indonesia, Philippines, and Vietnam exhibited some of the highest global seroprevalence rates, as did Gabon, Ghana and Nigeria in West Africa. Overall, the lowest seroprevalence rates in countries located in the Eastern Mediterranean (8.1%) and European (10.5%) regions. However, Romania in Eastern Europe exhibited one of the highest seroprevalence rates globally. Examining the root causes of this variation, particularly in areas or countries with similar characteristics, would be useful in the formulation of improved public health policies. We assumed that the factors responsible for this variation likely represent differences in public health and sanitation status, cultural and social conditions, environmental hygiene and in climate. For this reason, we conducted several subgroup and meta-regression analyses to assess the impact of these parameters on *T*-seroprevalence. Many of these parameters have an overlapping effect on seroprevalence rates and relate to risk factors of HT (Table 4).

Our analyses indicated that the *T*-seroprevalence has had a non-significant increasing trend in recent years, which might have a number of possible explanations. One explanation could be an increasing tendency of world population to have a pet or have close contact to dogs (e.g., shepherd or stray) and/or cats. As it is well known, these animals are key reservoirs and definitive hosts of *Toxocara* spp. [34, 35]. In agreement with this statement, we have shown that contact with dogs and cats are likely risk factors for seropositivity (Table 4). Furthermore, urbanisation accelerates in recent years in throughout the world, reflected in increasing numbers of both pet and stray animals in many of countries [36]. These animals live in or transit through public places, such as beaches, parks and children’s playgrounds; a proportion of these animals defaecate in these places, leaving behind eggs that become infective and contaminate the environment [37, 38]. In this regard, we have shown that contact with soil is a potential risk factor for the seropositivity, and meta-regression analysis showed an increasing trend of prevalence with an increasing contamination rate of soil with *Toxocara* eggs. Moreover, the results of this study are relatively consistent with the global prevalence of *Toxocara* eggs in public places reported in a previous study [4], which indicated that approximately one-fifth of public areas in the world are contaminated with *Toxocara* eggs, with soil as a major source for HT.

With regard to geographical and climatic parameters, the results of the present study are consistent with a previous proposal [4] that contamination in public places with *Toxocara* eggs is a highly significant risk factor for seropositivity In previous study, we showed that a high prevalence *Toxocara* eggs in public places was significantly associated with a low geographical latitude, high longitude, and high relative humidity, but not significantly linked to a high rate of precipitation [4]. The findings of the present study indicated that the *T*-seroprevalence is significantly lower in countries located in a higher latitude, which could be explained in a number of ways. Countries at higher latitudes such as in the European region and North American countries, are recognized as more developed, with people having ready accesses to health care centers and being relatively well educated about preventive measures against infectious agents. In contrast, some countries located in lower latitudes (0–30°) might be less privileged and less advanced in public health education, personal hygiene, and social and environmental hygiene [39–41]. In accord with this statement, some findings of present study demonstrate that countries with high levels of income and HDI have a significantly lower *T*-seroprevalence than countries with reverse levels. Another explanation might be that regions at higher latitudes are colder than countries at lower latitudes; clearly, temperature is a key factor in the life cycle of *Toxocara* spp. [42, 43]. The optimum temperatures for the complete embryonation of *Toxocara* eggs in soil is 20–30°C, while temperatures of < 10°C or warmer than 37°C are detrimental to the embryonation and survival of *Toxocara* eggs [42, 43]. The findings of present study also showed that the lowest *T*-seroprevalence (6.9%) was found in regions with an annual mean temperature of < 7°C, and the highest prevalence rate (35.7%) was found in regions with an annual mean temperature of 25–30°C. Furthermore, our meta-regression analysis indicated a significant increasing trend of *T*-seroprevalence with an increasing mean annual temperature.

With regard to longitude, some results of present study demonstrated a non-significant increasing trend in *T*-seroprevalence with increase in geographical longitude. This might be explained by substantial contamination of soil or public places in countries located at higher longitudes, as reported previously [4]. In that study [4] we indicated that public places in the South American, Western Pacific and South-East Asia regions were likely heavily contaminated with *Toxocara* eggs, which was explained by these regions having suitable climatic and environmental conditions for the survival of *Toxocara* eggs. It is well known that moisture in soil is a key factor for the long-term survival of *Toxocara* eggs [42, 44]. In the abovementioned regions, mean temperatures are optimal for *Toxocara*, and the relative humidity and annual precipitation are high, while in East Mediterranean countries, where the *T*-seroprevalence was estimated to be low, it is warm and dry, and precipitation is low. Another for the situation in South America or East Asia regions might be that contact with pets and stray animals is close [45–47], and in some countries of East Asia such as Korea, China and Vietnam dog and cat meat is commonly consumed [48–50], while in East Mediterranean countries, due to religious beliefs, close contact with dogs and cats seems to be relatively limited. In addition, in some of East Mediterranean countries, there are legal restrictions to having pets and taking them to public places [51, 52].

In subgroup analyses, we have found that studies of children had higher *T*-seroprevalence rates, and risk factor analyses revealed that young age is a likely risk factor for seropositivity. This could be explained by this fact that children have more contact with soil and pets and also have lower personal hygiene scores [1, 53, 54]. Moreover, the present risk factor analyses showed that males and people living in rural area are at a greater risk for exposure to HT, which might be explained by agricultural activities and more contact with *Toxocara* egg-contaminated soil and stray animals [55–57]. Another subgroup analysis showed that the *T*-seroprevalence in studies that used the Western blot method (27.8%) was higher than in those that used TES-ELISA (18.6%). This difference in prevalence estimates was somewhat expected, and might be explained by a bias in the number of studies (*n* = 240; sample size: 263,235) that employed ELISA compared with Western blot (*n* = 10; sample size: 2,092). Another important consideration is that there was a marked variation in the protocols employed in different studies. The main technical issues that can influence test results and need to be considered include: (i) specificity and non-specific background; and (ii) sensitivity of each assay (cf. [19]). Although these parameters were often not reported in the publications included in the present study, they are highly dependent on a range of aspects, including the amount and batch of antigen used; the blocking agent employed; the antibody conjugate utilised (and whether it is affinity-purified or not); incubation times and temperatures; enzyme system used; and the approach used to measure band intensity and/or size (Western blot) or absorbance (ELISA). In the present study, it was not possible to appraise all of these technical aspects in individual studies, because, in many cases, inadequate detail was provided.

In our opinion, this study has a number of strengths. The main strength is that it represents the most comprehensive assessment of *T*-seroprevalence worldwide using statistical methods. We included data for healthy people and excluded high risk groups, with the intent of minimising prevalence overestimates. However, this may have led to *T*-seroprevalence underestimates; therefore, our findings might not be extrapolated to the context of individuals in all communities. We applied a random-effects model to provide a conservative estimate of the global *T*-seroprevalence. In addition, we indicate the impact of geographical, climatic and socio-demographic parameters on the *T*-seroprevalence, using pooled data to highlight differences within and among different regions around the world. However, this study has some limitations. First, despite our comprehensive search, there was a paucity or absence of data for a number of countries, and many of the available studies had limited sample sizes and a lack of data on sex, age and/or risk factors. In addition, in some countries only one eligible study was identified, which could compromise somewhat the interpretation of our estimates. However, we suggested that the *T*-seroprevalence in countries for which limited or no data were available would be similar to pooled seroprevalence data in the same country or region. Second, studies included were undertaken during different time periods, with an absence of recent data for some countries, limiting the accuracy of inter-regional comparisons. Third, another limitation is this that some studies used different sero-diagnostic methods to detect and measure levels of serum antibody (IgG) against *Toxocara*. Some studies used in-house ELISA and some other used commercial ELISA kits and western blot; these methods can have marked differences in diagnostic sensitivity and specificity, possibly resulting in some inaccuracies in our *T*-seroprevalence estimates. Nonetheless, we performed subgroup analysis in an attempt to minimise such inaccuracies. Fourth, there was a high degree of heterogeneity in analyses among WHO-regions, sub-regions and among countries within the same WHO-region and in the other analyses conducted. Such heterogeneities are expected in global estimates among locations and across time, and likely relate to the use of distinct diagnostic methods (a variety of ELISA and Western blot methods), differences in the performance of these methods and differences in cut-off values for test-positivity among studies; variable *T*-seroprevalence associated with different geographic regions and the lack of data for some geographic regions; and differences in populations, study designs and sample sizes among studies included. Despite these limitations, this meta-analysis provides the most comprehensive estimates of the global *T*-seroprevalence to date, and suggests that one fifth of world’s population has been exposed to HT. This study provides useful information for national or international health policymakers to prioritise prevention efforts and intervention programs to reduce the HT burden. For example, WHO now leads a global initiative to implement preventive chemotherapy regimens to target soil-transmitted helminth and filarial infections, but none of these efforts target HT despite its susceptibility to albendazole and other anthelminthic drugs now used in mass drug treatment packages. Given HT’s public health importance and association with lung dysfunction, asthma, and cognitive and intellectual delays, there is potentially a strong rationale for including this neglected disease as an essential target. The present study also identified geographic regions for which there are little or no epidemiological data for HT. Thus, we advocate for well-designed epidemiological studies in areas with limited data and to establish and apply innovative strategies to reduce HT and *T*-seroprevalence, particularly in underprivileged countries, in order to contribute to some of the Sustainable Development Goals of the United Nations. Moreover, we suggest preventive veterinary and environmental measures (particularly in regions with high seroprevalence rates), in order to reduce the risk of human infection. Such measures could include: strategic anthelminthic treatment of both pet and stray dogs, and cats; rigorous and regular removal of animal faeces from soil, especially in public places, and regular exchange or sterilisation of sand or soil in children’s playgrounds.

## Supporting information

S1 Checklist PRISMA checklist. (DOCX)

S1 Table Main characteristics of all eligible studies reporting seroprevalence of *Toxocara* infection in healthy people. (DOCX)

S2 Table Global, regional and national pooled *T*-seroprevalence among healthy population (results from 250 studies performed in 71 countries). (DOCX)

S3 Table *T*-seroprevalence worldwide according to *a priori* defined subgroups. (DOCX)

S1 Fig *T*-seroprevalence in the Eastern and Western hemispheres. **Abbreviations:** CI, confidence interval; *T*-seropositive, number of seropositive people for toxocariasis
(TIF)

S2 Fig *T*-seroprevalence in the Northern and Southern hemispheres. **Abbreviations:** CI, confidence interval; *T*-seropositive, number of seropositive people for toxocariasis.
(TIF)

S3 Fig Ecological linear meta-regression analyses of the global *T*-seroprevalence: (panel A) implementation years of screening showing a non-statistically significant upward trend in seroprevalence in more recent years (*C* = 0.0007, *P*-value = 0.57); (panel B) country’s income level showing a showing a statistically significant downward trend in seroprevalence in countries with higher level of income (*C* = -0.082, *P*-value < 0.001); (panel C) human development index (HDI) showing a statistically significant downward trend in seroprevalence in countries with higher levels of HDI (*C* = -0.084, *P*-value < 0.001); (panel D) geographical latitude a showing a statistically significant downward trend in seroprevalence with increasing geographical latitude (*C* = -0.005, *P-*value < 0.001); (panel E) geographical longitude a showing a non-statistically significant upward trend in seroprevalence with increasing geographical longitude (*C* = 0.0005, *P*-value = 0.1); (panel F) the relative humidity showing a statistically significant upward trend in seroprevalence in areas with higher relative humidity (*C* = 0.005, *P*-value < 0.001); (panel G) the mean temperature showing a statistically significant upward trend in seroprevalence with increasing mean temperature (*C* = 0.01, *P*-value < 0.001); (panel H) the annual precipitation showing a statistically significant upward trend in seroprevalence with increasing rate of precipitation (*C* = 0.0001, *P*-value < 0.001). *C* = coefficient.
(TIF)

S1 Text Details of the databases searches. (DOCX)
