# Supplementary material for: Seroprevalence estimates for toxocariasis in people worldwide: A systematic review and meta-analysis
Source: PLoS Negl Trop Dis. 2019 Dec 19;13(12):e0007809. doi: 10.1371/journal.pntd.0007809 (PMC6922318; doi:10.1371/journal.pntd.0007809)
Supplement: S1 Table — (DOCX) [file pntd.0007809.s002.docx]

**­­­S1 Table.** Main characteristics of all eligible studies reporting seroprevalence of *Toxocara* infection in healthy people

| Author | Publish year | Study period | Type of population | WHO sub-region | Country | HDI | Income level | Rain fall (mm) | Humidity (%) | Mean temp (°C) | Latitude | Longitude | Total number of samples tested | Number of seropositive people |
| --- | --- | --- | --- | --- | --- | --- | --- | --- | --- | --- | --- | --- | --- | --- |
| African region |  |  |  |  |  |  |  |  |  |  |  |  |  |  |
| Magnaval [1] | 1994 | 1987-1989 | Adult | AFR-D | La Reunion | Low | Low | 859 | 68 | 12.3 | 21.07 S | 55.31 E | 387 | 359 |
| Kenny [2] | 1995 | 1993 | Children & Adult | AFR-E | Kenya | Medium | Lower middle | 402 | 70 | 17 | 3.07 N | 35.35 E | 228 | 17 |
| Ajayi [3] | 2000 | 1996 | Children & Adult | AFR-D | Nigeria | Low | Lower middle | 710 | 52 | 17.4 | 9.34 N | 9.04 E | 104 | 31 |
| Nicoletti *, ⁂[4] | 2007 | 2001 | Children & Adult | AFR-E | Burundi | Low | Low | 1382 | 70 | 16.9 | 2.82 S | 29.98 E | 191 | 97 |
| Yakubu [5] | 2009 | 2007 | Children & Adult | AFR-D | Nigeria | Low | Lower middle | 710 | 52 | 17.4 | 9.34 N | 9.04 E | 184 | 41 |
| Liao [6] | 2010 | 2008 | Children | AFR-E | Swaziland | Medium | Lower middle | 426 | 63 | 21 | 27.21 S | 31.13 E | 92 | 41 |
| Nkouawa [7] | 2011 | 2009 | Children & Adult | AFR-D | Cameroon | Medium | Lower middle | 1936 | 87 | 20.4 | 7.36 N | 12.35 E | 168 | 61 |
| Ngugi *[8] | 2013 | 2008-2011 | children | AFR-E | Sub-Saharan Africa | Medium | Low | 789 | 71 | 19 | 8.78 S | 34.5 E | 510 | 123 |
| Ngugi *[8] | 2013 | 2008-2011 | Adult | AFR-E | Sub-Saharan Africa | Medium | Low | 789 | 71 | 19 | 8.78 S | 34.5 E | 611 | 160 |
| Wagner *[9] | 2014 | 2008 | Children & Adult | AFR-E | South Africa | Medium | Upper middle | 339 | 70 | 17 | 28.4 S | 31.2 E | 260 | 77 |
| Noormahomed [10] | 2014 | 2011-2013 | Adult | AFR-E | Mozambique | Low | Low | 1312 | 77 | 24.6 | 6.5 N | 21.9 E | 601 | 44 |
| Kamuyu *[11] | 2014 | 2012 | Children & Adult | AFR-E | Sub-Saharan Africa | Medium | Low | 789 | 71 | 19 | 8.78 S | 34.5 E | 1121 | 250 |
| Gyang ⁂[12] | 2015 | 2013-2014 | Children | AFR-D | Nigeria | Low | Lower middle | 1683 | 82 | 27 | 6.49 N | 3.38 E | 366 | 315 |
| Kyei [13] | 2015 | 2012 | Children | AFR-D | Ghana | Medium | Lower middle | 1165 | 79 | 27 | 7.94 N | 1.02 W | 566 | 303 |
| Okewole [14] | 2016 | 2003 | Children | AFR-D | Nigeria | Low | Lower middle | 1311 | 76 | 26.5 | 7.37 N | 3.9 E | 128 | 56 |
| Lotsch [15] | 2016 | 2014-2015 | Adult | AFR-D | Gabon | High | Upper middle | 1215 | 83 | 27 | 0.44 S | 10.43 E | 332 | 199 |
| Sowemimo ⁂[16] | 2017 | 2016 | Children | AFR-D | Nigeria | Low | Lower middle | 1292 | 76 | 24.7 | 7.45 N | 4.33 E | 308 | 115 |
| Kakooza-Mwesige *[17] | 2017 | 2009 | Children | AFR-E | Uganda | Low | Low | 1313 | 80 | 22.3 | 0.2 N | 33.4 E | 130 | 24 |
| Kakooza-Mwesige *[17] | 2017 | 2009 | Adult | AFR-E | Uganda | Low | Low | 1313 | 80 | 22.3 | 0.2 N | 33.4 E | 73 | 26 |
| [South-East Asia](http://www.who.int/about/regions/searo/en/)  region |  |  |  |  |  |  |  |  |  |  |  |  |  |  |
| Chomel [18] | 1993 | 1990 | Children | SEAR-B | Indonesia | Medium | Lower middle | 474 | 80 | 27 | 8.34 S | 115.09 E | 190 | 120 |
| Uga [19] | 1996 | 1992-1993 | Children & Adult | SEAR-B | Indonesia | Medium | Lower middle | 2717 | 80 | 26.3 | 7.47 S | 112.66 E | 244 | 154 |
| Rai [20] | 1996 | 1994 | Children & Adult | SEAR-D | Nepal | Medium | Low | 1154 | 67 | 19 | 26.48 N | 88.09 E | 200 | 162 |
| Malla [21] | 2002 | 1996-1999 | Children & Adult | SEAR-D | India | Medium | Lower middle | 1076 | 71 | 24.6 | 27.9 N | 80.79 E | 94 | 6 |
| Iddawela [22] | 2003 | 2000 | Children | SEAR-B | Sri Lanka | High | Lower middle | 517 | 79 | 28 | 5.94 N | 81.84 E | 1020 | 441 |
| Hayashi [23] | 2005 | 2003 | Children | SEAR-B | Indonesia | Medium | Lower middle | 2376 | 75 | 28 | 2.07 S | 120.16 E | 158 | 99 |
| Dar [24] | 2008 | 2004-2005 | Children | SEAR-D | India | Medium | Lower middle | 740 | 69 | 14 | 32.44 N | 74.54 E | 286 | 94 |
| Dar [25] | 2008 | 2006 | Children | SEAR-D | India | Medium | Lower middle | 740 | 69 | 14 | 32.44 N | 74.54 E | 110 | 36 |
| Fernando *[26] | 2009 | 2006-2007 | Children | SEAR-B | Sri Lanka | High | Lower middle | 517 | 79 | 28 | 5.94 N | 81.84 E | 96 | 10 |
| Singh [27] | 2015 | 2013 | Children & Adult | SEAR-D | India | Medium | Lower middle | 729 | 60 | 24 | 31.14 N | 75.34 E | 122 | 27 |
| Doi [28] | 2016 | 2013-2014 | Children | SEAR-B | Thailand | High | Upper middle | 1572 | 72 | 26.2 | 17.1 N | 104.1 E | 361 | 23 |
| Modi*[29] | 2018 | 2010-2013 | Children & Adult | SEAR-D | India | Medium | Lower middle | 729 | 60 | 24 | 31.4 N | 75.34 E | 211 | 21 |
| [Western Pacific](http://www.who.int/about/regions/wpro/en/)  region |  |  |  |  |  |  |  |  |  |  |  |  |  |  |
| Matsumura [30] | 1982 | 1980 | Children | WPR-A | Japan | Very high | High | 1830 | 70 | 14.9 | 34.18 N | 131.47 E | 83 | 3 |
| Matsumura [30] | 1982 | 1980 | Adult | WPR-A | Japan | Very high | High | 1830 | 70 | 14.9 | 34.18 N | 131.47 E | 530 | 20 |
| Nicholas [31] | 1986 | 1984 | Adult | WPR-A | Australia | Very high | High | 638 | 68 | 13 | 35.28 S | 149.12 E | 660 | 46 |
| Hakim [32] | 1992 | 1990 | Children & Adult | WPR-B | Malaysia | Very high | Upper middle | 1492 | 81 | 28 | 4 N | 102.29 E | 480 | 153 |
| Hakim [33] | 1993 | 1989-1991 | Children & Adult | WPR-B | Malaysia | Very high | Upper middle | 1492 | 81 | 28 | 4 N | 102.29 E | 331 | 65 |
| Auer [34] | 1995 | 1991-1992 | Children & Adult | WPR-B | Philippines | Medium | Lower middle | 1435 | 83 | 28 | 13.05 N | 121.4 E | 162 | 108 |
| Luo [35] | 1999 | 1996 | Children | WPR-B | China | High | Upper middle | 961 | 78 | 16.4 | 30.66 N | 104.06 E | 519 | 63 |
| Chan *[36] | 2001 | 1998 | Children | WPR-B | Malaysia | Very high | Upper middle | 1834 | 82 | 28 | 4.21 N | 101.97 E | 58 | 5 |
| Park [37] | 2002 | 1999 | Adult | WPR-B | South Korea | Very high | High | 920 | 65 | 12 | 37.29 N | 128.14 E | 314 | 16 |
| Fan [38] | 2004 | 1998-2000 | Children | WPR-B | Taiwan | Very high | High | 487 | 77 | 23 | 22 N | 121.75 E | 329 | 252 |
| Fan [39] | 2004 | 1998-2000 | Adult | WPR-B | Taiwan | Very high | High | 487 | 77 | 23 | 22 N | 121.75 E | 537 | 247 |
| Fan [40] | 2005 | 1998-2000 | Children | WPR-B | Taiwan | Very high | High | 487 | 77 | 23 | 22 N | 121.75 E | 73 | 42 |
| Zarkovic [41] | 2007 | 2005 | Adult | WPR-A | New Zealand | Very high | High | 871 | 76 | 15 | 38.05 S | 175.43 E | 140 | 1 |
| Romano [42] | 2010 | 2008 | Children & Adult | WPR-B | Malaysia | Very high | Upper middle | 1834 | 82 | 28 | 4.21 N | 101.97 E | 188 | 9 |
| Kim [43] | 2014 | 2012 | Adult | WPR-B | South Korea | Very high | High | 570 | 71 | 12 | 33.25 N | 129.36 E | 610 | 53 |
| Fu ⁂[44] | 2014 | 2012 | Children | WPR-B | Marshall Islands | High | Upper middle | 3365 | 75 | 27.3 | 7.13 N | 171.18 E | 166 | 144 |
| Cong [45] | 2014 | 2011-2013 | Children & Adult | WPR-B | China | High | Upper middle | 656 | 76 | 12 | 36.29 N | 118 E | 2866 | 351 |
| Won [46] | 2015 | 2010-2012 | Adult | WPR-B | South Korea | Very high | High | 570 | 71 | 12 | 33.25 N | 129.36 E | 228 | 153 |
| Jung [47] | 2015 | 2013 | Adult | WPR-B | South Korea | Very high | High | 1003 | 66 | 13 | 36.51 N | 126.8 E | 231 | 18 |
| Fu ⁂[48] | 2015 | 2009 | Adult | WPR-B | Taiwan | Very high | High | 2574 | 77 | 22 | 25.04 N | 121.53 E | 203 | 17 |
| Lee [49] | 2015 | 2012-2013 | Adult | WPR-B | South Korea | Very high | High | 1373 | 71 | 11.9 | 37.56 N | 126.97 E | 633 | 324 |
| Cong [50] | 2015 | 2013-2014 | Cildren | WPR-B | China | High | Upper middle | 259 | 65 | 12 | 18.24 N | 134.28 E | 1458 | 281 |
| Nguyen [51] | 2016 | 2012 | Children & Adult | WPR-B | Vietnam | Medium | Lower middle | 1868 | 78 | 27.4 | 10.82 N | 106.62 E | 77356 | 34995 |
| Yang [52] | 2016 | 2013-2015 | Children & Adult | WPR-B | China | High | Upper middle | 259 | 65 | 12 | 18.24 N | 134.28 E | 1842 | 296 |
| South Americas region |  |  |  |  |  |  |  |  |  |  |  |  |  |  |
| Lynch [53] | 1988 | 1984 | Children & Adult | AMR-B | Venezuela | High | High | 933 | 75 | 20.9 | 6.42 N | 66.58 W | 476 | 56 |
| Chieffi [54] | 1990 | 1983 | Children & Adult | AMR-B | Brazil | High | Upper middle | 1340 | 76 | 18.5 | 23.54 S | 46.63 W | 2025 | 70 |
| Agudelo [55] | 1990 | 1987-1988 | Children & Adult | AMR-B | Colombia | High | Upper middle | 866 | 81 | 13.5 | 4.6 N | 74.08 W | 207 | 98 |
| Virginia [56] | 1991 | 1989 | Children | AMR-B | Brazil | High | Upper middle | 1804 | 83 | 25.8 | 8.05 S | 34.88 W | 54 | 21 |
| Lynch [57] | 1993 | 1991 | Children | AMR-B | Venezuela | High | High | 933 | 75 | 20.9 | 10.48 N | 66.9 W | 368 | 74 |
| Cancrini [58] | 1998 | 1996 | Children & Adult | AMR-D | Bolivia | Medium | Lower middle | 1130 | 82 | 22 | 19.13 S | 61.15 W | 216 | 73 |
| Navarrete [59] | 1998 | 1997 | Adult | AMR-B | Chile | Very high | High | 277 | 80 | 24.6 | 39.8 S | 73.2 W | 188 | 10 |
| Moreira-Silva [60] | 1998 | 1996-1997 | Children | AMR-D | Brazil | High | Upper middle | 1103 | 75 | 24.8 | 19.1 S | 40.3 W | 100 | 39 |
| Lescano [61] | 1998 | 1989-1990 | Adult | AMR-D | Peru | High | Upper middle | 16 | 82 | 18.7 | 12.04 S | 77.02 W | 1023 | 75 |
| Taranto [62] | 2000 | 1998 | Children | AMR-B | Argentina | Very high | High | 502 | 75 | 22 | 34.6 S | 58.38 W | 98 | 20 |
| Radman [63] | 2000 | 1998 | Children & Adult | AMR-B | Argentina | Very high | High | 946 | 73 | 16.3 | 34.92 S | 57.95 W | 156 | 61 |
| Minvielle [64] | 2000 | 1998 | Adult | AMR-B | Argentina | Very high | High | 979 | 70 | 27 | 34.6 S | 58.38 W | 180 | 19 |
| Alonso [65] | 2000 | 1998 | Children | AMR-B | Argentina | Very high | High | 851 | 72 | 19.6 | 38.41 S | 63.61 W | 206 | 78 |
| Nicoletti *[66] | 2002 | 2000 | Children & Adult | AMR-D | Bolivi | Medium | Lower middle | 1130 | 82 | 22 | 19.13 S | 61.15 W | 233 | 28 |
| Anaruma Filho [67] | 2002 | 1998-1999 | Children & Adult | AMR-B | Brazil | High | Upper middle | 1315 | 74 | 19.3 | 22.9 S | 47.06 W | 138 | 33 |
| Baboolal [68] | 2002 | 1997-1998 | Children | AMR-B | Trinidad and Tobago | High | High | 1519 | 81 | 25.9 | 10.69 N | 61.22 W | 1009 | 629 |
| Campos Júnior [69] | 2003 | 2000 | Children | AMR-B | Brazil | High | Upper middle | 643 | 76 | 21 | 14.23 S | 51.92 W | 602 | 75 |
| Taranto [70] | 2003 | 2000 | Children & Adult | AMR-B | Argentina | Very high | High | 689 | 71 | 16.9 | 24.78 S | 65.42 W | 154 | 34 |
| Alderete [71] | 2003 | 1998-1999 | Children | AMR-B | Brazil | High | Upper middle | 1340 | 76 | 18.5 | 23.54 S | 46.63 W | 399 | 155 |
| Coelho [72] | 2004 | 2000-2001 | Children | AMR-B | Brazil | High | Upper middle | 1224 | 74 | 19.4 | 23.5 S | 47.45 W | 180 | 69 |
| García-Pedrique [73] | 2004 | 2002 | Children | AMR-B | Venezuela | High | Upper middle | 115 | 73 | 29 | 10.39 N | 71.36 W | 73 | 7 |
| Aguiar-Santos [74] | 2004 | 1987-1996 | Children & Adult | AMR-B | Brazil | High | Upper middle | 1804 | 83 | 25.8 | 8.05 S | 34.88 W | 386 | 152 |
| Alonso [75] | 2004 | 2002-2003 | Adult | AMR-B | Argentina | High | Upper middle | 1324 | 78 | 21.3 | 27.4 S | 58.9 W | 355 | 138 |
| Lopez Mde [76] | 2005 | 2003 | Children | AMR-B | Argentina | Very high | High | 1324 | 75 | 21.3 | 27.45 S | 58.98 W | 182 | 122 |
| Figueiredo [77] | 2005 | 2000-2001 | Children | AMR-B | Brazil | High | Upper middle | 1340 | 76 | 18.5 | 23.54 S | 46.63 W | 102 | 49 |
| Coelho [78] | 2005 | 2002 | Children | AMR-B | Brazil | High | Upper middle | 1660 | 75 | 25.3 | 8.11 S | 35.01 W | 215 | 53 |
| Muradian [79] | 2005 | 2001 | Children | AMR-B | Brazil | High | Upper middle | 1340 | 76 | 18.5 | 23.54 S | 46.63 W | 338 | 91 |
| Teixeira [80] | 2006 | 2004 | Children | AMR-B | Brazil | High | Upper middle | 457 | 71 | 22 | 18.51 S | 44.55 W | 242 | 21 |
| Chiodo [81] | 2006 | 2004 | Children & Adult | AMR-B | Argentina | Very high | High | 1040 | 72 | 16.8 | 34.61 S | 58.37 W | 100 | 23 |
| Fillaux ⁂[82] | 2007 | 2002-2004 | Children & Adult | AMR-B | Argentina | Very high | High | 851 | 72 | 19.6 | 38.41 S | 63.61 W | 114 | 36 |
| Ferreira [83] | 2007 | 2002-2004 | Children | AMR-B | Brazil | High | Upper middle | 1462 | 82 | 24.4 | 8.44 S | 67.21 W | 483 | 104 |
| Paludo [84] | 2007 | 2004-2005 | Children | AMR-B | Brazil | High | Upper middle | 1276 | 68 | 17.7 | 23.42 S | 51.93 W | 450 | 130 |
| Damian [85] | 2007 | 2006 | Children & Adult | AMR-B | Brazil | High | Upper middle | 1122 | 83 | 27 | 3.11 S | 60.02 W | 100 | 49 |
| Prestes-Carneiro [86] | 2008 | 2004 | Children & Adult | AMR-B | Brazil | High | Upper middle | 1340 | 76 | 18.5 | 23.54 S | 46.63 W | 79 | 17 |
| Rubinsky-Elefant [87] | 2008 | 2004 | Children & Adult | AMR-B | Brazil | High | Upper middle | 1122 | 82 | 28 | 3.11 S | 60.02 W | 403 | 108 |
| Espinoza [88] | 2008 | 2005 | Children | AMR-D | Peru | High | Upper middle | 22 | 76 | 22.3 | 6.7 S | 79.9 W | 182 | 59 |
| Roldan [89] | 2008 | 2006 | Children | AMR-D | Peru | High | Upper middle | 16 | 82 | 18.7 | 12.04 S | 77.02 W | 646 | 201 |
| Prestes-Carneiro [90] | 2009 | 2006-2008 | Children & Adult | AMR-B | Brazil | High | Upper middle | 1340 | 76 | 18.5 | 23.54 S | 46.63 W | 182 | 25 |
| Roldan [91] | 2009 | 2005 | Adult | AMR-D | Peru | High | Upper middle | 801 | 60 | 13.8 | 7.37 S | 78.02 W | 256 | 115 |
| Correa [92] | 2009 | 2007 | Children | AMR-B | Brazil | High | Upper middle | 1315 | 74 | 19.3 | 22.9 S | 47.06 W | 100 | 28 |
| Colli [93] | 2010 | 2005-2007 | Children | AMR-B | Brazil | High | Upper middle | 2034 | 84 | 20.5 | 25.44 S | 49.27 W | 376 | 194 |
| Diaz-Suarez [94] | 2010 | 2009 | Children & Adult | AMR-B | Venezuela | High | High | 405 | 73 | 28.2 | 10.6 N | 71.6 W | 110 | 24 |
| Espinoza [95] | 2010 | 2007 | Children & Adult | AMR-D | Peru | High | Upper middle | 324 | 82 | 11.3 | 11.46 S | 76.62 W | 303 | 62 |
| Lopez *[96] | 2010 | 2008 | Children | AMR-B | Argentina | Very high | High | 851 | 72 | 19.6 | 38.41 S | 63.61 W | 53 | 28 |
| Roldan [97] | 2010 | 2008 | Children & Adult | AMR-D | Peru | High | Upper middle | 2098 | 82 | 26.6 | 5.9 S | 76.12 W | 300 | 107 |
| Santaram [98] | 2011 | 2008 | Children | AMR-B | Brazil | High | Upper middle | 1340 | 76 | 18.5 | 23.54 S | 46.63 W | 252 | 28 |
| Souza [99] | 2011 | 2009 | Children & Adult | AMR-B | Brazil | High | Upper middle | 1781 | 78 | 25.2 | 12.97 S | 38.51 W | 338 | 201 |
| Dattoli [100] | 2011 | 2009 | Children & Adult | AMR-B | Brazil | High | Upper middle | 1781 | 78 | 25.2 | 12.97 S | 38.51 W | 268 | 124 |
| Fragoso [101] | 2011 | 2009 | Children | AMR-B | Brazil | High | Upper middle | 1007 | 75 | 25.8 | 22.11 S | 46.68 W | 391 | 202 |
| Mattia [102] | 2011 | 2005-2007 | Children | AMR-B | Brazil | High | Upper middle | 2034 | 84 | 20.5 | 25.44 S | 49.27 W | 353 | 130 |
| Manini [103] | 2012 | 2010-2011 | Children | AMR-B | Brazil | High | Upper middle | 1512 | 75 | 20.7 | 23.76 S | 53.32 W | 90 | 16 |
| Mendonca [104] | 2012 | 1997-2005 | Children | AMR-B | Brazil | High | Upper middle | 1781 | 78 | 25.2 | 12.97 S | 38.51 W | 890 | 409 |
| Guilherme [105] | 2013 | 2009-2010 | Children | AMR-B | Brazil | High | Upper middle | 2034 | 84 | 20.5 | 25.44 S | 49.27 W | 167 | 7 |
| Negri [106] | 2013 | 2010 | Adult | AMR-B | Brazil | High | Upper middle | 1340 | 76 | 18.5 | 23.54 S | 46.63 W | 253 | 22 |
| Prestes-Carneiro [107] | 2013 | 2008-2009 | Children & Adult | AMR-B | Brazil | High | Upper middle | 1340 | 76 | 18.5 | 23.54 S | 46.63 W | 194 | 28 |
| Schoenardie [108] | 2013 | 2011 | Children | AMR-B | Brazil | High | Upper middle | 1378 | 78 | 18 | 31.77 S | 52.34 W | 427 | 216 |
| Cassenote [109] | 2014 | 2007-2010 | Children | AMR-B | Brazil | High | Upper middle | 1340 | 76 | 18.5 | 23.54 S | 46.63 W | 252 | 39 |
| Martin [110] | 2014 | 2011-2013 | Children & Adult | AMR-B | Argentina | High | Upper middle | 970 | 74 | 18.5 | 31.6 S | 60.6 W | 857 | 444 |
| Marchioro [111] | 2014 | 2012-2013 | Children | AMR-B | Brazil | High | Upper middle | 2034 | 84 | 20.5 | 25.44 S | 49.27 W | 544 | 136 |
| Oliart-Guzman [112] | 2014 | 203-2010 | Children | AMR-B | Brazil | High | Upper middle | 1122 | 82 | 28 | 3.11 S | 60.02 W | 539 | 134 |
| Archelli [113] | 2014 | 2012 | Children | AMR-B | Argentina | Very high | High | 946 | 73 | 16.3 | 34.92 S | 57.95 W | 120 | 46 |
| Araujo [114] | 2015 | 2013 | Children & Adult | AMR-B | Venezuela | High | High | 604 | 72 | 28 | 8.83 N | 61.51 W | 50 | 5 |
| Martinez [115] | 2015 | 2013 | Children | AMR-B | Venezuela | High | High | 1065 | 69 | 26.9 | 10.14 N | 67.35 W | 224 | 65 |
| Santos [116] | 2015 | 2013-2014 | Adult | AMR-B | Brazil | High | Upper middle | 1205 | 78 | 18.3 | 32.03 S | 52.09 W | 280 | 18 |
| Cadore *[117] | 2016 | 2012-2013 | Children | AMR-B | Brazil | High | Upper middle | 1205 | 78 | 18.3 | 32.03 S | 52.09 W | 52 | 4 |
| Cermeño [118] | 2016 | 2007 | Children & Adult | AMR-B | Venezuela | High | High | 189 | 74 | 26 | 10.03 N | 64.42 W | 146 | 43 |
| Pereira [119] | 2016 | 2013 | Adult | AMR-B | Brazil | High | Upper middle | 1668 | 67 | 21.1 | 15.77 S | 47.92 W | 311 | 23 |
| Vargas [120] | 2016 | 2011-2012 | Children & Adult | AMR-B | Chile | Very high | High | 2204 | 76 | 11.7 | 39.52 S | 73.23 W | 355 | 90 |
| Silva [121] | 2017 | 2010 | Children | AMR-B | Brazil | High | Upper middle | 1781 | 78 | 25.2 | 12.97 S | 38.51 W | 791 | 503 |
| Santos [122] | 2017 | 2015 | Children | AMR-B | Brazil | High | Upper middle | 1205 | 78 | 18.3 | 32.03 S | 52.09 W | 280 | 56 |
| Martinez [123] | 2018 | 2016 | Children | AMR-B | Venezuela | High | High | 1065 | 69 | 26.9 | 10.14 N | 67.35 W | 259 | 37 |
| Araujo [124] | 2018 | 2012-2014 | Adult | AMR-B | Brazil | High | Upper middle | 1205 | 78 | 18.3 | 32.03 S | 52.09 W | 344 | 247 |
| North America region |  |  |  |  |  |  |  |  |  |  |  |  |  |  |
| Berrocal[125] | 1980 | 1978-1979 | Children | AMR-A | Puerto Rico | Very high | High | 165 | 80 | 20.8 | 18.2 N | 66.5 W | 641 | 43 |
| Desowitz*[126] | 1981 | 1979 | Children | AMR-A | USA | Very high | High | 293 | 75 | 17.5 | 19.89 N | 155.58 W | 96 | 3 |
| Ellis [127] | 1986 | 1981 | Children | AMR-A | USA | Very high | High | 1403 | 66 | 17 | 35.75 N | 79.01 W | 333 | 77 |
| Thompson [128] | 1986 | 1984 | Children | AMR-B | St. Lucia | High | Upper Middle | 1794 | 76 | 27 | 13.59 N | 61 W | 82 | 71 |
| Herrmann [129] | 1985 | 1971-1973 | Children | AMR-A | USA | Very high | High | 585 | 50 | 15 | 37.09 N | 95.71 W | 1369 | 88 |
| Tanner[130] | 1987 | 1985 | Children & Adult | AMR-A | Canada | Very high | High | 1101 | 64 | 4 | 46.81 N | 71.21 W | 2600 | 260 |
| Bass[131] | 1987 | 1978-1984 | Children | AMR-A | USA | Very high | High | 585 | 50 | 15 | 37.09 N | 95.71 W | 153 | 14 |
| Marmor[132] | 1987 | 1980-1981 | Children | AMR-A | USA | Very high | High | 1144 | 61 | 12 | 40.71 N | 74 W | 4648 | 506 |
| Embil[133] | 1988 | 1982-1984 | Children | AMR-A | Canada | Very high | High | 1351 | 71 | 6 | 45.1 N | 63 W | 973 | 165 |
| Montalvo[134] | 1994 | 1992 | Children | AMR-A | Cuba | High | Upper middle | 1244 | 79 | 24.9 | 23.11 N | 82.36 W | 156 | 8 |
| Levesque[135] | 2007 | 2005 | Adult | AMR-A | Canada | Very high | High | 1101 | 64 | 4 | 46.81 N | 71.21 W | 50 | 2 |
| Jones[136] | 2008 | 1988-1994 | Children & Adult | AMR-A | USA | Very high | High | 585 | 50 | 15 | 37.09 N | 95.71 W | 16646 | 2330 |
| Tinoco-Gracia[137] | 2008 | 2001-2002 | Children | AMR-B | Mexico | High | Upper middle | 67 | 65 | 24 | 32.52 N | 117.04 W | 288 | 25 |
| Munoz-Guzman*[138] | 2010 | 2008 | Children | AMR-B | Mexico | High | Upper middle | 625 | 54 | 15.9 | 23.63 N | 102.55 W | 152 | 30 |
| Campagna[139] | 2011 | 2007 | Children & Adult | AMR-A | Canada | Very high | High | 1101 | 64 | 4 | 46.81 N | 71.21 W | 251 | 8 |
| Messier[140] | 2012 | 2004 | Adult | AMR-A | Canada | Very high | High | 369 | 64 | 4 | 55 N | 64 W | 917 | 36 |
| Sampasa-Kanyinga[141] | 2012 | 2005-2008 | Adult | AMR-A | Canada | Very high | High | 1101 | 64 | 4 | 46.81 N | 71.21 W | 267 | 11 |
| Sariego [142] | 2012 | 2003-2004 | Children | AMR-A | Cuba | High | Upper middle | 1299 | 75 | 25 | 21.33 N | 79.36 W | 1011 | 392 |
| Alvarado-Esquivel[143] | 2013 | 2011 | Children & Adult | AMR-B | Mexico | High | Upper middle | 463 | 46 | 17.6 | 37.27 N | 107.88 W | 126 | 33 |
| Romero Núñez [144] | 2013 | 2010 | Children | AMR-B | Mexico | High | Upper middle | 608 | 54 | 15.5 | 19.61 N | 99.06 W | 108 | 24 |
| Schurer[145] | 2013 | 2010-2011 | Children & Adult | AMR-A | Canada | Very high | High | 377 | 70 | 2.9 | 54.4 N | 106 W | 201 | 26 |
| Kanobana [146] | 2013 | 2011 | Children | AMR-A | Cuba | High | Upper middle | 1299 | 75 | 25 | 21.33 N | 79.36 W | 958 | 384 |
| Alvarado-Esquivel *[147] | 2014 | 2010-2013 | Adult | AMR-B | Mexico | High | Upper middle | 463 | 46 | 17.6 | 37.27 N | 107.88 W | 183 | 3 |
| Heredia [148] | 2014 | 2008-2010 | Adult | AMR-B | Mexico | High | Upper middle | 625 | 54 | 15.9 | 23.63 N | 102.55 W | 200 | 20 |
| Schurer [149] | 2014 | 2011-2013 | Children & Adult | AMR-A | Canada | Very high | High | 377 | 70 | 2.9 | 54.4 N | 106 W | 113 | 5 |
| Goyette [150] | 2014 | 2007-2008 | Adult | AMR-A | Canada | High | High | 26 | 68 | 3 | 65.24 N | 60.46 W | 2213 | 38 |
| Nava Cortés [151] | 2015 | 2013-2014 | Children | AMR-B | Jamaica | Highe | Upper middle | 1112 | 71 | 7.1 | 18.1 N | 77.29 W | 183 | 22 |
| Ortega-Pacheco [152] | 2015 | 2013 | Adult | AMR-B | Mexico | High | Upper middle | 980 | 74 | 25.8 | 21.21 N | 87.74 W | 89 | 26 |
| Cook [153] | 2016 | 2010 | Children & Adult | AMR-B | Jamaica | High | Upper middle | 1112 | 71 | 7.1 | 18.1 N | 77.29 W | 1544 | 327 |
| Farmer [154] | 2017 | 2011-2014 | Children & Adult | AMR-A | USA | Very high | High | 585 | 50 | 15 | 37.09 N | 95.71 W | 13509 | 858 |
| Guo [155] | 2017 | 2008-2011 | Adult | AMR-B | Caribbean Countries | High | Upper Middle | 422 | 76 | 27 | 15.32 N | 76.15 W | 435 | 63 |
| Berrett [156] | 2017 | 2011-2014 | Children & Adult | AMR-A | USA | Very high | High | 585 | 50 | 15 | 37.09 N | 95.71 W | 13509 | 693 |
| European region |  |  |  |  |  |  |  |  |  |  |  |  |  |  |
| Josephs [157] | 1981 | 1979 | Children | EUR-A | United Kingdom | Very high | High | 621 | 73 | 11.1 | 55.37 N | 3.43 W | 133 | 19 |
| Sturchler [158] | 1981 | 1979 | Children | EUR-A | Switzerland | Very high | High | 1018 | 75 | 10 | 46.81 N | 8.22 E | 134 | 5 |
| Ree [159] | 1984 | 1982-1983 | Children & Adult | EUR-A | United Kingdom | Very high | High | 414 | 79 | 10 | 54 N | 4 W | 1405 | 344 |
| Sturchler [160] | 1986 | 1984 | Children & Adult | EUR-A | Switzerland | Very high | High | 778 | 78 | 10 | 47.55 N | 7.57 E | 765 | 39 |
| Caucanas [161] | 1988 | 1986 | Adult | EUR-A | France | Very high | High | 525 | 79 | 14 | 46.22 N | 2.21 E | 255 | 21 |
| Knney [162] | 1987 | 1985 | Children | EUR-A | Ireland | Very high | High | 1257 | 82 | 11 | 53.14 N | 7.69 W | 302 | 57 |
| Portus [163] | 1989 | 1987 | Children & Adult | EUR-A | Spain | Very high | High | 612 | 74 | 16.5 | 41.38 N | 2.15 E | 1018 | 37 |
| Garcia [164] | 1989 | 1987 | Children | EUR-A | Spain | Very high | High | 408 | 68 | 12.1 | 40.96 N | 5.66 W | 650 | 16 |
| Ljungstrom [165] | 1989 | 1985-1986 | Children & Adult | EUR-A | Sweden | Very high | High | 492 | 82 | 8 | 60.12 N | 18.64 E | 175 | 43 |
| Van Gemund [166] | 1989 | 1987-1988 | Children | EUR-A | Netherlands | Very high | High | 777 | 83 | 9.5 | 52 N | 4.3 E | 234 | 26 |
| Genchi [167] | 1990 | 1987 | Adult | EUR-A | Italy | Very high | High | 258 | 75 | 15 | 41.87 N | 12.56 E | 2112 | 84 |
| Chobanov [168] | 1990 | 1987 | Children & Adult | EUR-B | Azerbaijan | High | Upper middle | 258 | 79 | 15.1 | 40.4 N | 49.86 E | 4765 | 320 |
| Stefancikova [169] | 1993 | 1986-1990 | Children & Adult | EUR-B | Slovakia | Very high | High | 678 | 71 | 11 | 48.66 N | 19.69 E | 3442 | 610 |
| Havasiova [170] | 1993 | 1991-1992 | Children & Adult | EUR-B | Slovakia | Very high | High | 678 | 71 | 11 | 48.66 N | 19.69 E | 908 | 124 |
| Gueglio [171] | 1994 | 1992 | Children & Adult | EUR-A | France | Very high | High | 525 | 79 | 14 | 46.22 N | 2.21 E | 1801 | 401 |
| Buijs [172] | 1994 | 1987-1989 | Children | EUR-A | Netherlands | Very high | High | 549 | 82 | 10 | 52.13 N | 5.29 E | 639 | 42 |
| Holland [173] | 1995 | 1992 | Children | EUR-A | Ireland | Very high | High | 1257 | 82 | 11 | 53.14 N | 7.69 W | 2129 | 660 |
| Guerra [174] | 1995 | 1992 | Children | EUR-A | Spain | Very high | High | 450 | 55 | 13.7 | 40.41 N | 3.7 W | 100 | 1 |
| Gundlach [175] | 1996 | 1994 | Children & Adult | EUR-B | Poland | Very high | High | 540 | 79 | 7.6 | 51.2 N | 22.5 E | 346 | 38 |
| Wolfrom *[176] | 1996 | 1992-1993 | Children & Adult | EUR-A | France | Very high | High | 931 | 77 | 12.7 | 44.83 N | 0.57 W | 81 | 17 |
| Cilla [177] | 1996 | 1991-1994 | Children | EUR-A | Spain | Very high | High | 1477 | 79 | 14 | 43.17 N | 2.21 W | 546 | 66 |
| Fenoy [178] | 1996 | 1994 | Children & Adult | EUR-A | Spain | Very high | High | 500 | 55 | 14 | 40.46 N | 3.74 W | 1413 | 156 |
| Buijs [179] | 1997 | 1989-1992 | Children | EUR-A | Netherlands | Very high | High | 804 | 80 | 9.3 | 52.09 N | 5.12 E | 1285 | 97 |
| Jimenez [180] | 1997 | 1995 | Children & Adult | EUR-A | Spain | Very high | High | 118 | 63 | 21 | 28.05 N | 15.23 W | 14074 | 459 |
| Vassalou [181] | 1998 | 1996 | Children | EUR-A | Greece | Very high | High | 380 | 60 | 19 | 37.59 N | 23.43 E | 341 | 9 |
| Humbert *[182] | 2000 | 1994-1995 | Children & Adult | EUR-A | France | Very high | High | 525 | 79 | 14 | 46.22 N | 2.21 E | 236 | 30 |
| Giacometti [183] | 2000 | 1998-1999 | Children & Adult | EUR-A | Italy | Very high | High | 757 | 75 | 14.4 | 43.59 N | 13.5 E | 163 | 7 |
| Hermanowska-Szpakowicz [184] | 2001 | 1999 | Children & Adult | EUR-B | Poland | Very high | High | 549 | 78 | 7.8 | 52.46 N | 22.51 E | 1025 | 214 |
| Theodoridis [185] | 2001 | 1999 | Children | EUR-A | Greece | Very high | High | 445 | 67 | 15.9 | 40.64 N | 22.93 E | 511 | 64 |
| Oshevskaia [186] | 2003 | 2001 | Children & Adult | EUR-C | Russia | Very high | Upper middle | 621 | 74 | 5.1 | 54.2 N | 37.61 E | 2586 | 644 |
|  |  |  |  |  |  |  |  |  |  |  |  |  |  |  |
| Habluetzel [187] | 2003 | 1998-1999 | Adult | EUR-A | Italy | Very high | High | 963 | 76 | 12.8 | 43.3 N | 13.72 E | 428 | 7 |
| Juncker-Voss [188] | 2004 | 2002 | Adult | EUR-A | Austria | Very high | High | 623 | 73 | 9.9 | 48.2 N | 16.37 E | 60 | 13 |
| Deutz [189] | 2005 | 1999 | Adult | EUR-A | Austria | Very high | High | 858 | 79 | 8.3 | 47.06 N | 15.45 E | 50 | 1 |
| Ballcka_Ramlsz [190] | 2005 | 2001-2003 | Children | EUR-B | Poland | Very high | High | 542 | 81 | 8.6 | 53.4 N | 14.5 E | 134 | 25 |
| Gonzalez-Quintela [191] | 2006 | 2003 | Children & Adult | EUR-A | Spain | Very high | High | 49 | 67 | 19 | 42.68 N | 8.48 W | 463 | 134 |
| Yariktas *[192] | 2007 | 2005 | Children & Adult | EUR-B | Turkey | High | Upper middle | 747 | 58 | 14.1 | 38.96 N | 35.24 E | 61 | 7 |
| Akyol *[193] | 2007 | 2003 | Children & Adult | EUR-B | Turkey | High | Upper middle | 747 | 58 | 14.1 | 38.96 N | 35.24 E | 50 | 4 |
| Dogan [194] | 2007 | 2005 | Children | EUR-B | Turkey | High | Upper middle | 747 | 58 | 14.1 | 38.96 N | 35.24 E | 571 | 74 |
| Nicoletti *, ⁂[195] | 2008 | 2006 | Children & Adult | EUR-A | Italy | Very high | High | 258 | 75 | 15 | 41.87 N | 12.56 E | 201 | 13 |
| Zarnowska [196] | 2008 | 2002-2005 | Children | EUR-B | Poland | Very high | High | 549 | 78 | 7.8 | 52.46 N | 22.51 E | 343 | 100 |
| Jarosz [197] | 2009 | 2007 | Children | EUR-B | Poland | Very high | High | 549 | 78 | 7.8 | 52.46 N | 17.78 E | 242 | 35 |
| Sviben [198] | 2009 | 2004-2007 | Children | EUR-A | Croatia | Very high | High | 930 | 78 | 11 | 45.81 N | 15.98 E | 142 | 46 |
| Stensvold [199] | 2009 | 2001 | Children & Adult | EUR-A | Denmark | Very high | High | 605 | 82 | 9 | 56.26 N | 9.5 E | 3247 | 87 |
| Torgerson [200] | 2009 | 2005 | Children & Adult | EUR-C | Kazakhstan | Very high | Upper middle | 49 | 58 | 11 | 45.48 N | 80.51 E | 3126 | 349 |
| Bellanger *[201] | 2010 | 1999 | Children & Adult | EUR-A | France | Very high | High | 525 | 79 | 14 | 46.22 N | 2.21 E | 113 | 31 |
| Demirci [202] | 2010 | 2005 | Children & Adult | EUR-B | Turkey | High | Upper middle | 537 | 63 | 11.9 | 37.45 N | 30.33 E | 619 | 97 |
| Magnaval ⁂[203] | 2011 | 2007 | Adult | EUR-C | Russia | Very high | Upper middle | 321 | 66 | 4 | 63.8 N | 121.6 E | 90 | 4 |
| Cojocariu [204] | 2012 | 2009-2012 | Adult | EUR-B | Romania | Very high | Upper middle | 559 | 75 | 9.9 | 47.15 N | 27.6 E | 457 | 236 |
| Cobzaru * [205] | 2012 | 2010 | Children | EUR-B | Romania | Very High | Upper middle | 559 | 75 | 9.9 | 47.15 N | 27.6 E | 88 | 4 |
| Celik* [206] | 2013 | 2012 | Adult | EUR-B | Turkey | High | Upper middle | 534 | 57 | 12.4 | 38.6 N | 39.2 E | 50 | 0 |
| Poeppl [207] | 2013 | 2011 | Adult | EUR-A | Austria | Very high | High income | 623 | 73 | 9.9 | 48.2 N | 16.37 E | 1046 | 66 |
| Kaneva *, [208] | 2015 | 2013 | Children & Adult | EUR-B | Bulgaria | Very high | Upper middle | 581 | 70 | 10.2 | 42.73 N | 25.48 E | 50 | 2 |
| Van Den Broucke [209] | 2015 | 2000-2013 | Adult | EUR-A | Belgium | Very high | High | 778 | 78 | 10.1 | 51.21 N | 4.4 E | 3436 | 190 |
| Selek *[210] | 2015 | 2013 | Adult | EUR-B | Turkey | High | Upper middle | 747 | 58 | 14.1 | 38.96 N | 35.24 E | 109 | 0 |
| Sozen ⁂[211] | 2015 | 2014 | Adult | EUR-B | Turkey | High | Upper middle | 1109 | 61 | 15.3 | 37.21 N | 28.36 E | 376 | 30 |
| Antolova [212] | 2015 | 2011 | Adult | EUR-B | Slovakia | High | High | 678 | 71 | 11 | 48.66 N | 19.69 E | 823 | 99 |
| Magnaval ⁂[213] | 2016 | 2012 | Adult | EUR-C | Russia | Very high | Upper middle | 175 | 69 | -15.2 | 67.55 N | 133.39 E | 77 | 0 |
| Gabrielli [214] | 2016 | 2015 | Children & Adult | EUR-B | Serbia | High | Upper middle | 246 | 67 | 14 | 44.48 N | 20.27 E | 338 | 76 |
| Lassen [215] | 2016 | 2004-2011 | Children & Adult | EUR-C | Estonia | Very high | High | 492 | 82 | 7 | 57.77 N | 22.5 E | 1247 | 163 |
| Mughini-Gras [216] | 2016 | 1995-2007 | Children & Adult | EUR-A | Netherlands | Very high | High | 549 | 82 | 10 | 52.13 N | 5.29 E | 4842 | 419 |
| Kroten [217] | 2016 | 2013 | Children | EUR-B | Poland | Very high | High | 615 | 80 | 8 | 53.13 N | 23.16 E | 190 | 10 |
| Martelli [218] | 2017 | 2012-2014 | Adult | EUR-A | Italy | Very high | High | 258 | 75 | 15 | 41.87 N | 12.56 E | 113 | 11 |
| Papavasilopoulos [219] | 2018 | 2014 | Children & Adult | EUR-A | Greece | Very high | High | 380 | 60 | 19 | 37.59 N | 23.43 E | 250 | 40 |
| Overbosch [220] | 2018 | 2008 | Adult | EUR-A | Netherlands | Very high | High | 805 | 83 | 9.2 | 52.3 N | 4.9 E | 604 | 1 |
| Eastern  Mediterranean  region |  |  |  |  |  |  |  |  |  |  |  |  |  |  |
| Abo-Shehada [221] | 1992 | 1986-1987 | Children & Adult | EMR-B | Jordan | High | Upper middle | 428 | 65 | 17.9 | 32.55 N | 35.85 E | 699 | 76 |
| Chantal [222] | 1996 | 1994 | Adult | EMR-D | Djibouti | Low | Lower middle | 121 | 62 | 30.1 | 11.8 N | 42.5 E | 108 | 0 |
| Sajjadi [223] | 2000 | 1998 | Children | EMR-B | Iran | High | Upper middle | 316 | 38 | 16.8 | 29.61 N | 52.53 E | 519 | 133 |
| Akhlaghi [224] | 2006 | 2003-2004 | Children | EMR-B | Iran | High | Upper middle | 469 | 40 | 13.7 | 34.26 N | 46.8 E | 260 | 22 |
| Kanafani [225] | 2006 | 2004 | Children & Adult | EMR-B | Lebanon | High | Upper middle | 845 | 66 | 20.5 | 33.88 N | 35.49 E | 150 | 28 |
| Fallah [226] | 2007 | 2003 | Children | EMR-B | Iran | High | Upper middle | 360 | 47 | 12 | 34.79 N | 48.51 E | 544 | 29 |
| Nourian [227] | 2008 | 2007 | Children | EMR-B | Iran | High | Upper middle | 419 | 51 | 11.5 | 36.67 N | 48.47 E | 810 | 22 |
| Sharif [228] | 2010 | 2005-2006 | Children | EMR-B | Iran | High | Upper middle | 690 | 76 | 16.7 | 36.56 N | 53.06 E | 1210 | 302 |
| Alavi [229] | 2011 | 2007-2008 | Children | EMR-B | Iran | High | Upper middle | 215 | 39 | 24.9 | 31.31 N | 48.68 E | 203 | 4 |
| El-Shazly [230] | 2013 | 2008 | Children & Adult | EMR-D | Egypt | Medium | Lower middle | 183 | 69 | 21 | 26.82 N | 30.8 E | 455 | 35 |
| El-Tantawy *[231] | 2013 | 2012-2013 | Children | EMR-D | Egypt | Medium | Lower middle | 56 | 50 | 20.5 | 31.04 N | 31.37 E | 60 | 28 |
| Hosseini-Safa [232] | 2015 | 2013-2014 | Children | EMR-B | Iran | High | Upper middle | 125 | 37 | 15.6 | 32.65 N | 51.67 E | 427 | 6 |
| Awadallah [233] | 2015 | 2013 | Children & Adult | EMR-D | Egypt | Medium | Lower middle | 183 | 69 | 21 | 26.82 N | 30.8 E | 150 | 36 |
| Sarkari *[234] | 2015 | 2010-2012 | Children & Adult | EMR-D | Iran | High | Upper middle | 316 | 61 | 16.8 | 29.5 N | 52.5 E | 100 | 3 |
| Allahdin *[235] | 2015 | 2014 | Children & Adult | EMR-B | Iran | High | Upper middle | 215 | 39 | 24.9 | 31.31 N | 48.68 E | 144 | 2 |
| Mosayebi*[236] | 2016 | 2013-2014 | Children | EMR-B | Iran | High | Upper middle | 316 | 43 | 11.8 | 34.09 N | 49.7 E | 70 | 0 |
| Momeni [237] | 2016 | 2014-2015 | Children & Adult | EMR-B | Iran | High | Upper middle | 390 | 58 | 11.6 | 37.32 N | 45.05 E | 397 | 12 |
| Galal [238] | 2016 | 2014-2015 | Children | EMR-D | Egypt | Medium | Lower middle | 183 | 69 | 21 | 26.82 N | 30.8 E | 81 | 49 |
| Berenji *[239] | 2016 | 2013 | Adult | EMR-B | Iran | High | Upper middle | 251 | 50 | 13.5 | 32.6 N | 59.6 E | 93 | 1 |
| Abbasi *[240] | 2017 | 2014-2015 | Children | EMR-B | Iran | High | Upper middle | 315 | 51 | 12 | 36.68 N | 48.5 E | 238 | 2 |
| Shahraki [241] | 2017 | 2016 | Children | EMR-B | Iran | High | Upper middle | 105 | 27 | 18.2 | 29.51 N | 60.85 E | 364 | 14 |
| Beiromvand [242] | 2017 | 2014-2015 | Children & Adult | EMR-B | Iran | High | Upper middle | 319 | 36 | 23 | 31.31 N | 48.67 E | 410 | 8 |
| Khozime *[243] | 2017 | 2016-2017 | Children & Adult | EMR-B | Iran | High | Upper middle | 251 | 50 | 13.5 | 36.2 N | 59.6 E | 50 | 1 |
| Baghani [244] | 2018 | 2014-2015 | Children & Adult | EMR-B | Iran | High | Upper middle | 220 | 39 | 16.4 | 35.69 N | 51.42 E | 374 | 21 |
| Mahmoudvand [245] | 2018 | 2016-2017 | Children | EMR-B | Iran | High | Upper middle | 488 | 41 | 16.9 | 33.36 N | 49.33 E | 316 | 14 |
| Aghamolaie [246] | 2018 | 2016-2017 | Children & Adult | EMR-B | Iran | High | Upper middle | 690 | 76 | 16.7 | 36.56 N | 53.06 E | 630 | 148 |
| Shokouhi [247] | 2018 | 2016 | Children | EMR-B | Iran | High | Upper middle | 419 | 40 | 14.6 | 33.63 N | 46.41 E | 383 | 84 |
| Sarkari [248] | 2018 | 2016 | Children | EMR-B | Iran | High | Upper middle | 300 | 38 | 22 | 29.17 N | 51.42 E | 617 | 39 |
| Raissi *[249] | 2018 | 2017-2018 | Adult | EMR-B | Iran | High | Upper middle | 419 | 40 | 14.6 | 33.63 N | 46.41 E | 270 | 49 |
| Khoshsima-shahraki [250] | 2019 | 2015-2016 | Children | EMR-B | Iran | High | Upper middle | 113 | 40 | 26.5 | 26.3 N | 60 E | 963 | 17 |

**Abbreviations:** HDI, human development index; temp., temperature

* Case-control studies

⁂ Studies that infection was diagnosed by Western blot

**References**

1. Magnaval J-F, Michault A, Calon N, Charlet J-P. Epidemiology of human toxocariasis in La Reunion. Trans R Soc Trop Med Hyg. 1994; 88(5):531–3.

2. Kenny J, MacCabe R, Smith H, Holland C. Serological evidence for the presence of toxocariasis in the Turkana district of Kenya. Trans R Soc Trop Med Hyg. 1995; 89(4):377–8.

3. Ajayi O, Duhlinska D, Agwale SM, Njoku M. Frequency of human toxocariasis in Jos, Plateau state, Nigeria. Mem Inst Oswaldo Cruz. 2000; 95(2):147–9.

4. Nicoletti A, Bartoloni A, Sofia V, Mantella A, Nsengiyumva G, Frescaline G, et al. Epilepsy and toxocariasis: a case‐control study in Burundi. Epilepsia. 2007; 48(5):894–9.

5. Yakubu R, Audu P, Ndams I, Nock I. Seroprevalence of human *Toxocara canis* infection in vom, plateau state, Nigeria. Nigerian J Sci Res. 2009; 8:11–4.

6. Liao C-W, Sukati H, D'lamini P, Chou C-M, Liu Y-H, Huang Y-C, et al. Seroprevalence of *Toxocara canis* infection among children in Swaziland, southern Africa. Ann Trop Med Parasitol. 2010; 104(1):73–80.

7. Nkouawa A, Sako Y, Moyou-Somo R, Ito A. Serological and molecular tools to detect neurologic parasitic zoonoses in rural Cameroon. Southeast Asian J Trop Med Public Health. 2011; 42(6):1365–74.

8. Ngugi AK, Bottomley C, Kleinschmidt I, Wagner RG, Kakooza-Mwesige A, Ae-Ngibise K, et al. Prevalence of active convulsive epilepsy in sub-Saharan Africa and associated risk factors: cross-sectional and case-control studies. Lancet Neurol. 2013; 12(3):253–63.

9. Wagner RG, Ngugi AK, Twine R, Bottomley C, Kamuyu G, Gómez-Olivé FX, et al. Prevalence and risk factors for active convulsive epilepsy in rural northeast South Africa. Epilepsy Res. 2014; 108(4):782–91.

10. Noormahomed EV, Nhacupe N, Mascaro-Lazcano C, Mauaie MN, Buene T, Funzamo CA, et al. A cross-sectional serological study of cysticercosis, schistosomiasis, toxocariasis and echinococcosis in HIV-1 infected people in Beira, Mozambique. PLoS Negl Trop Dis. 2014; 8(9):e3121.

11. Kamuyu G, Bottomley C, Mageto J, Lowe B, Wilkins PP, Noh JC, et al. Exposure to multiple parasites is associated with the prevalence of active convulsive epilepsy in sub-Saharan Africa. PLoS Negl Trop Dis. 2014; 8(5):e2908.

12. Gyang PV, Akinwale OP, Lee Y-L, Chuang T-W, Orok AB, Ajibaye O, et al. Seroprevalence, disease awareness, and risk factors for *Toxocara canis* infection among primary schoolchildren in Makoko, an urban slum community in Nigeria. Acta Trop. 2015; 146:135–40.

13. Kyei G, Ayi I, Boampong J, Turkson P. Sero-epidemiology of *Toxocara canis* infection in children attending four selected health facilities in the central region of Ghana. Ghana Med J. 2015; 49(2):77–83.

14. Okewole E. The prevalence, pathogenesis and control of canine and human toxocariosis in Ibadan, Nigeria. Sokoto J Vet Sci. 2016; 14(2):34–42.

15. Lötsch F, Obermüller M, Mischlinger J, Mombo-Ngoma G, Groger M, Adegnika AA, et al. Seroprevalence of *Toxocara* spp. in a rural population in Central African Gabon. Parasitol Int. 2016; 65(6):632–4.

16. Sowemimo OA, Lee Y-L, Asaolu SO, Chuang T-W, Akinwale OP, Badejoko BO, et al. Seroepidemiological study and associated risk factors of *Toxocara canis* infection among preschool children in Osun state, Nigeria. Acta Trop. 2017; 173:85–9.

17. Kakooza‐Mwesige A, Ndyomugyenyi D, Pariyo G, Peterson SS, Waiswa PM, Galiwango E, et al. Adverse perinatal events, treatment gap, and positive family history linked to the high burden of active convulsive epilepsy in Uganda: A population‐based study. Epilepsia Open. 2017; 2(2):188–98.

18. Chomel BB, Kasten R, Adams C, Lambillotte D, Theis J, Goldsmith R, et al. Serosurvey of some major zoonotic infections in children and teenagers in Bali, Indonesia. Southeast Asian J Trop Med Public Health. 1993; 24(2):321–6.

19. Uga S, Ono K, Kataoka N, Hasan H. Seroepidemiology of five major zoonotic parasite infections in inhabitants of Sidoarjo, East Java, Indonesia. Southeast Asian J Trop Med Public Health. 1996; 27(3):556–61.

20. Rai SK, Uga S, Ono K, Nakanishi M, Shrestha HG, Matsumura T. Seroepidemiological study of *Toxocara* infection in Nepal. Southeast Asian J Trop Med Public Health. 1996; 27(2):286–90.

21. Malla N, Aggarwal A, Mahajan R. A serological study of human toxocariasis in north India. Natl Med J India. 2002; 15(3):145–7.

22. Iddawela DR, Kumarasiri P, Wijesundera MdS. A seroepidemiological study of toxocariasis and risk factors for infection in children in Sri Lanka. Southeast Asian J Trop Med Public Health. 2003; 34(1):7–15.

23. Hayashi E, Tuda J, Imada M, Akao N, Fujita K. The high prevalence of asymptomatic *Toxocara* infection among schoolchildren in Manado, Indonesia. Southeast Asian J Trop Med Public Health. 2005; 36(6):1399–406.

24. Dar Z, Tanveer S, Yattoo G, Sofi B, Wani S, Dar P. Seroprevalence of toxocariasis in children in Kashmir, J&K State, India. Iran J Parasitol. 2008; 3(4):45–50.

25. Dar Z, Tanveer S, Yattoo G, Sofi B, Dar P, Wani S. Presence of anti-*Toxocara* antibodies in children population of district Anantnag and Pulwama of Kashmir valley. Indian J Med Microbiol. 2008; 26(4):400–2.

26. Fernando D, Wickramasinghe P, Kapilananda G, Dewasurendra RL, Amarasooriya M, Dayaratne A. *Toxocara* seropositivity in Sri Lankan children with asthma. Pediatr Int. 2009; 51(2):241–5.

27. Singh B, Sharma R, Gill J. *Toxocara canis,* *Trichinella spiralis* and *Taenia solium* helminthozoonoses: seroprevalence among selected populations in north India. J Parasit Dis. 2015; 39(3):487–90.

28. Doi R, Itoh M, Chakhatrakan S, Uga S. Epidemiological investigation of parasitic infection of schoolchildren from six elementary schools in Sakon Nakhon Province, Thailand. Kobe J Med Sci. 2016; 62(5):120–8.

29. Modi M, Singh R, Goyal MK, Gairolla J, Singh G, Rishi V, et al. Prevalence of epilepsy and its association with exposure to *Toxocara canis:* A community based, case–control study from rural Northern India. Ann Indian Acad Neurol. 2018; 21(4):263–9.

30. Matsumura K, Endo R. Seroepidemiological study on toxocaral infection in man by enzyme-linked immunosorbent assay. Epidemiol Infect. 1983; 90(1):61–5.

31. Nicholas WL, Stewart AC, Walker JC. Toxocariasis: a serological survey of blood donors in the Australian Capital Territory together with observations on the risks of infection. Trans R Soc Trop Med Hyg. 1986; 80(2):217–21.

32. Hakim SL, Mak J, Lam P, Nazma S, Normaznah Y. Seroprevalence of *Toxocara canis* antibodies among Orang Asli (aborigines) in Peninsular Malaysia. Southeast Asian J Trop Med Public Health. 1992; 23:493–6.

33. Hakim S, Mak J, Lam P. ELISA seropositivity for *Toxocara canis* antibodies in Malaysia, 1989-1991. Med J Malaysia. 1993; 48(3):303–7.

34. Auer H, Radda A, Escalona TG, Aspöck H. Seroepidemiological studies in Oriental Mindoro (Philippines)–prevalence of parasitic zoonoses. Z Tropenmed Parasitol. 1995; 17:153–8.

35. Luo Z-j, Wang G-x, Yang C-l, Luo C-h, Cheng S-w, Liao L. Detection of circulating antigens and antibodies in *Toxocara canis* infection among children in Chengdu, China. J Parasitol. 1999; 85(2):252–6.

36. Chan PW, Anuar AK, Fong MY, Debruyne JA, Ibrahim J. *Toxocara* seroprevalence and childhood asthma among Malaysian children. Pediatr Int. 2001; 43(4):350–3.

37. Park H-Y, Lee S-U, Huh S, Kong Y, Magnaval J-F. A seroepidemiological survey for toxocariasis in apparently healthy residents in Gangwon-do, Korea. Korean J Parasitol. 2002; 40(3):113–7.

38. Fan CK, Hung CC, Du WY, Liao CW, Su KE. Seroepidemiology of *Toxocara canis* infection among mountain aboriginal schoolchildren living in contaminated districts in eastern Taiwan. Trop Med Int Health. 2004; 9(12):1312–8.

39. Fan C-K, Lan H-S, Hung C-C, Chung W-C, Liao C-W, Du W-Y, et al. Seroepidemiology of *Toxocara canis* infection among mountain aboriginal adults in Taiwan. Am J Trop Med Hyg. 2004; 71(2):216–21.

40. Fan C-K, Liao C-W, Kao T-C, Li M-H, Du W-Y, Su K-E. Sero-epidemiology of *Toxocara canis* infection among aboriginal schoolchildren in the mountainous areas of north–eastern Taiwan. Ann Trop Med Parasitol. 2005; 99(6):593–600.

41. Zarkovic A, MacMurray C, Deva N, Ghosh S, Whitley D, Guest S. Seropositivity rates for Bartonella henselae, *Toxocara canis* and *Toxoplasma gondii* in New Zealand blood donors. Clin Exp Ophthalmol. 2007; 35(2):131–4.

42. Romano N, Azah MN, Rahmah N, Lim Y, Rohela M. Seroprevalence of toxocariasis among Orang Asli (Indigenous people) in Malaysia using two immunoassays. Trop Biomed. 2010; 27(3):585–94.

43. Kim HS, Jin Y, Choi M-H, Kim J-H, Lee YH, Yoon CH, et al. Significance of serum antibody test for toxocariasis in healthy healthcare examinees with eosinophilia in Seoul and Gyeongsangnam-do, Korea. J Korean Med Sci. 2014; 29(12):1618–25.

44. Fu C-J, Chuang T-W, Lin H-S, Wu C-H, Liu Y-C, Langinlur MK, et al. Seroepidemiology of *Toxocara canis* infection among primary schoolchildren in the capital area of the Republic of the Marshall Islands. BMC Infect Dis. 2014; 14(1):261.

45. Cong W, Zhang X-X, Zhou N, Yu C-Z, Chen J, Wang X-Y, et al. *Toxocara* seroprevalence among clinically healthy individuals, pregnant women and psychiatric patients and associated risk factors in Shandong Province, Eastern China. PLoS Negl Trop Dis. 2014; 8(8):e3082.

46. Won EJ, Kim J, Shin M-G, Shin JH, Suh SP, Ryang DW. Seroepidemiology of toxocariasis and its clinical implications in Gwangju and Jeonnam-Province, Korea. Ann Lab Med. 2015; 35(4):449–53.

47. Jung S-Y, Ahn M-J, Oh J-Y, Nam H-S, Hong S-T, Yun Y-H, et al. Infection status of endoparasites in foreigner workers living in Cheonan City, Chungnam Province, Korea. Korean J Parasitol. 2015; 53(2):243–6.

48. FU C-J, Cheng-Yan K, Yueh-Lun L, Chien-Wei L, Po-Ching C, Ting-Wu CHUANG Y-CW, et al. Seroprevalence and associated risk factors of toxocariasis among college students in Taipei City, Taiwan. Iran J Parasitol. 2015; 10(3):482–9.

49. Lee J-Y, Yang MH, Hwang J-H, Kang M, Paeng J-W, Yune S, et al. The prevalence of toxocariasis and diagnostic value of serologic tests in asymptomatic Korean adults. Allergy Asthma Immunol Res. 2015; 7(5):467–75.

50. Cong W, Meng Q-F, You H-L, Zhou N, Dong X-Y, Dong W, et al. Seroprevalence and risk factors of *Toxocara* infection among children in Shandong and Jilin provinces, China. Acta Trop. 2015; 152:215–9.

51. Nguyen T, Cheong FW, Liew JWK, Lau YL. Seroprevalence of fascioliasis, toxocariasis, strongyloidiasis and cysticercosis in blood samples diagnosed in Medic Medical Center Laboratory, Ho Chi Minh City, Vietnam in 2012. Parasit Vectors. 2016; 9(1):486.

52. Yang G-L, Zhang X-X, Shi C-W, Yang W-T, Jiang Y-L, Wei Z-T, et al. Seroprevalence and associated risk factors of *Toxocara* infection in Korean, Manchu, Mongol, and Han ethnic groups in northern China. Epidemiol Infect. 2016; 144(14):3101–7.

53. Lynch NR, Eddy K, Hodgen AN, Lopez RI, Turner KJ. Seroprevalence of *Toxocara canis* infection in tropical Venezuela. Trans R Soc Trop Med Hyg. 1988; 82(2):275–81.

54. Chieffi PP, Ueda M, Camargo ED, Souza AMCd, Guedes ML, Gerbi LJ, et al. Visceral larva migrans: a seroepidemiological survey in five municipalities of São Paulo State, Brazil. Rev Inst Med Trop Sao Paulo. 1990; 32(3):204–10.

55. Agudelo C, Villareal E, Cáceres E, López C, Eljach J, Ramírez N, et al. Human and dogs *Toxocara canis* infection in a poor neighborhood in Bogota. Mem Inst Oswaldo Cruz. 1990; 85(1):75–8.

56. Virginia P, Nagakura K, Ferreira O, Tateno S. Serologic evidence of toxocariasis in northeast Brazil. Jpn J Med Sci Biol. 1991; 44(1):1–6.

57. Lynch NR, Hagel I, Vargas V, Rotundo A, Varela MC, Di Prisco MC, et al. Comparable seropositivity for ascariasis and toxocariasis in tropical slum children. Parasitol Res. 1993; 79(7):547–50.

58. Cancrini G, Bartoloni A, Zaffaroni E, Guglielmetti P, Gamboa N, Nicoletti A, et al. Seroprevalence of *Toxocara canis*-IgG antibodies in two rural Bolivian communities. Parassitologia. 1998; 40(4):473–5.

59. Navarrete N, Rojas E. Toxocarosis seroprevalence in blood donors. Arch Med Vet. 1998; 30(1):153–6.

60. Moreira-Silva SF, Leao ME, Mendonca HF, Pereira FE. Prevalence of anti-*Toxocara* antibodies in a random sample of inpatients at a children's hospital in Vitória, Espírito Santo, Brazil. Rev Inst Med Trop Sao Paulo. 1998; 40(4):263–4.

61. Lescano SAZ, Chieffi PP, Peres BA, de Mello EO, Velarde CN, Salinas AA, et al. Soil contamination and human infection by *Toxocara* sp. in the urban area of Lima, Peru. Mem Inst Oswaldo Cruz. 1998; 93(6):733–4.

62. Taranto NJ, Passamonte L, Marinconz R, De Marzi MC, Cajal SP, Malchiodi EL. Zoonotic parasitoses transmitted by dogs in the Chaco Salteño. Medicina (B Aires). 2000; 60(2):217–20.

63. Radman NE, Archelli SM, Fonrouge RD, Guardis MdV, Linzitto OR. Human toxocarosis. Its seroprevalence in the city of La Plata. Mem Inst Oswaldo Cruz. 2000; 95(3):281–5.

64. Minvielle M, Taus M, Raffo A, Ciarmela M, Basualdo J. Seroprevalence of toxocariasis in blood donors of Gualeguaychú, Argentina. Trans R Soc Trop Med Hyg. 2000; 94(4):373–5.

65. Alonso JM, Bojanich MV, Chamorro M, Gorodner JO. *Toxocara* seroprevalence in children from a subtropical city in Argentina. Rev Inst Med Trop Sao Paulo. 2000; 42(4):235–7.

66. Nicoletti A, Bartoloni A, Reggio A, Bartalesi F, Roselli M, Sofia V, et al. Epilepsy, cysticercosis, and toxocariasis: a population-based case-control study in rural Bolivia. Neurology. 2002; 58(8):1256–61.

67. Anaruma Filho F, Chieffi PP, Correa CRS, Camargo ED, Silveira EP, ARANHA JJB, et al. Human toxocariasis: a seroepidemiological survey in the municipality of Campinas (SP), Brazil. Rev Inst Med Trop Sao Paulo. 2002; 44(6):303–7.

68. Baboolal S, Rawlins SC. Seroprevalence of toxocariasis in schoolchildren in Trinidad. Trans R Soc Trop Med Hyg. 2002; 96(2):139–43.

69. Campos Júnior D, Elefant GR, Silva EOdM, Gandolfi L, Jacob CMA, Tofeti A, et al. Freqüência de soropositividade para antígenos de *Toxocara canis* em crianças de classes sociais diferentes. Rev Soc Bras Med Trop. 2003; 36(4):509–13.

70. Taranto N, Cajal S, De Marzi M, Fernandez M, Frank F, Bru A, et al. Clinical status and parasitic infection in a Wichi Aboriginal community in Salta, Argentina. Trans R Soc Trop Med Hyg. 2003; 97(5):554–8.

71. Alderete J, Jacob C, Pastorino AC, Elefant GR, Castro AP, Fomin AB, et al. Prevalence of *Toxocara* infection in schoolchildren from the Butantã region, São Paulo, Brazil. Mem Inst Oswaldo Cruz. 2003; 98(5):593–7.

72. Coelho LM, Silva MV, Dini CY, Giacon Neto AA, Novo NF, Silveira EP. Human toxocariasis: a seroepidemiological survey in schoolchildren of Sorocaba, Brazil. Mem Inst Oswaldo Cruz. 2004; 99(6):533–57.

73. Garcia-Pedrique M, Diaz-Suarez O, Estevez J, Cheng-Ng R, Araujo-Fernández M, Castellano J, et al. Prevalence of infection by *Toxocara* in schoolchildren in the community of El Mojan, Zulia state, Venezuela. Invest Clin. 2004; 45(4):347–54.

74. Aguiar-Santos AM, Andrade LD, Medeiros Z, Chieffi PP, Lescano SZ, Perez EP. Human toxocariasis: frequency of anti-*Toxocara* antibodies in children and adolescents from an outpatient clinic for lymphatic filariasis in Recife, Northeast Brazil. Rev Inst Med Trop Sao Paulo. 2004; 46(2):81–5.

75. Alonso J, López M, Bojanich M, Marull J. *Toxocara canis* infección in adult healthy population from a Subtropical area in Argentina. Parasitol Latinoam. 2004; 5:61–4.

76. Lopez LM, Martin G, Chamorro CM, Mario JA. Toxocariasis in children from a subtropical region. Medicina. 2005; 65(3):226–30.

77. Figueiredo SD, Taddei JA, Menezes JJ, Novo NF, Silva EO, Cristóvão HL, et al. Clinical-epidemiological study of toxocariasis in a pediatric population. J Pediatr (Rio J). 2005; 81(2):126–32.

78. Coelho RAL, CARVALHO JR LB, Perez EP, Araki K, Takeuchi T, Ito A, et al. Prevalence of toxocariasis in northeastern Brazil based on serology using recombinant *Toxocara canis* antigen. Am J Trop Med Hyg. 2005; 72(1):103–7.

79. Muradian V, Gennari SM, Glickman LT, Pinheiro SR. Epidemiological aspects of visceral larva migrans in children living at Sao Remo Community, Sao Paulo (SP), Brazil. Vet Parasitol. 2005; 134(1-2):93–7.

80. Teixeira CR, Chieffi PP, Lescano SA, Silva EOdM, Fux B, Cury MC. Frequency and risk factors for toxocariasis in children from a pediatric outpatient center in southeastern Brazil. Rev Inst Med Trop Sao Paulo. 2006; 48(5):251–5.

81. Chiodo P, Basualdo J, Ciarmela L, Pezzani B, Apezteguía M, Minvielle M. Related factors to human toxocariasis in a rural community of Argentina. Mem Inst Oswaldo Cruz. 2006; 101(4):397–400.

82. Fillaux J, Santillan G, Magnaval J-F, Jensen O, Larrieu E, Sobrino-Becaria CD. Epidemiology of toxocariasis in a steppe environment: the Patagonia study. Am J Trop Med Hyg. 2007; 76(6):1144–7.

83. Ferreira MU, Rubinsky-Elefant G, de Castro TG, Hoffmann ÉHE, da Silva-Nunes M, Cardoso MA, et al. Bottle feeding and exposure to *Toxocara* as risk factors for wheezing illness among under-five Amazonian children: a population-based cross-sectional study. J Trop Pediatr. 2007; 53(2):119–24.

84. Paludo ML, Falavigna DL, Elefant GR, Gomes ML, Baggio ML, Amadei LB, et al. Frequency of *Toxocara* infection in children attended by the health public service of Maringá, south Brazil. Rev Inst Med Trop Sao Paulo. 2007; 49(6):343–8.

85. Damian MM, Martins M, Sardinha JF, Souza LOd, Chaves A, Tavares AdM. Freqüência de anticorpo anti-*Toxocara canis* em comunidade do Rio Uatumã, no Estado do Amazonas. Rev Soc Bras Med Trop. 2007; 40(6):661–4.

86. Prestes-Carneiro L, Santarém V, Zago S, Miguel N, Zambelli SdF, Villas R, et al. Sero-epidemiology of toxocariasis in a rural settlement in São Paulo state, Brazil. Ann Trop Med Parasitol. 2008; 102(4):347–56.

87. Rubinsky-Elefant G, da Silva-Nunes M, Malafronte RS, Muniz PT, Ferreira MU. Human toxocariasis in rural Brazilian Amazonia: seroprevalence, risk factors, and spatial distribution. Am J Trop Med Hyg. 2008; 79(1):93–8.

88. Espinoza YA, Huapaya PH, Roldán WH, Jiménez S, Arce Z, Lopez E. Clinical and serological evidence of *Toxocara* infection in school children from Morrope district, Lambayeque, Peru. Rev Inst Med Trop Sao Paulo. 2008; 50(2):101–5.

89. Roldán WH, Espinoza YA, Atúncar A, Ortega E, Martinez A, Saravia M. Frequency of eosinophilia and risk factors and their association with *Toxocara* infection in schoolchildren during a health survey in the north of Lima, Peru. Rev Inst Med Trop Sao Paulo. 2008; 50(5):273–8.

90. Prestes-Carneiro L, Souza D, Moreno G, Troiani C, Santarém V, Zago S, et al. Toxocariasis/cysticercosis seroprevalence in a long-term rural settlement, São Paulo, Brazil. Parasitology. 2009; 136(6):681–9.

91. Roldán WH, Espinoza YA, Huapaya PE, Huiza AF, Sevilla CR, Jiménez S. Frequency of human toxocariasis in a rural population from Cajamarca, Peru determined by DOT-ELISA test. Rev Inst Med Trop Sao Paulo. 2009; 51(2):67–71.

92. Correa CR, Bismarck CM. Toxocariasis: incidence, prevalence and the time serum remains positive in school children from Campinas, SP, Brazil. J Trop Pediatr. 2009; 56(3):215–6.

93. Colli CM, Rubinsky-Elefant G, Paludo ML, Falavigna DL, Guilherme EV, Mattia S, et al. Serological, clinical and epidemiological evaluation of toxocariasis in urban areas of south Brazil. Rev Inst Med Trop Sao Paulo. 2010; 52(2):69–74.

94. Diaz-Suarez O, Garcia ME, Melendez F, Estévez J. Seroepidemiology of toxocariasis in a Yucpa Amerindian community from the Perija mountains, western Venezuela. Kasmera. 2010; 38(2):138–46.

95. Espinoza YA, Huapaya PE, Roldán WH, Jiménez S, Abanto EP, Rojas CA, et al. Seroprevalence of human toxocariasis in Andean communities from the Northeast of Lima, Peru. Rev Inst Med Trop Sao Paulo. 2010; 52(1):31–6.

96. López LM, Bojanich M, Jacobacci J, Sercic C, Michelini A, Alonso J. *Toxocara canis* and bronchial asthma. Medicina. 2010; 70(1):75–8.

97. Roldán WH, Cavero YA, Espinoza YA, Jiménez S, Gutiérrez CA. Human toxocariasis: a seroepidemiological survey in the Amazonian city of Yurimaguas, Peru. Rev Inst Med Trop Sao Paulo. 2010; 52(1):37–42.

98. Santarém VA, Leli FNC, Rubinsky-Elefant G, Giuffrida R. Protective and risk factors for toxocariasis in children from two different social classes of Brazil. Rev Inst Med Trop Sao Paulo. 2011; 53(2):66–72.

99. Souza RF, Dattoli VCC, Mendonça LR, Jesus JRd, Baqueiro T, Santana CdC, et al. Prevalence and risk factors of human infection by *Toxocara canis* in Salvador, State of Bahia, Brazil. Rev Soc Bras Med Trop. 2011; 44(4):516–9.

100. Dattoli VCC, Freire S, Mendonça LR, Santos P, Meyer R, Alcantara‐Neves NM. *Toxocara canis* infection is associated with eosinophilia and total IgE in blood donors from a large Brazilian centre. Trop Med Int Health. 2011; 16(4):514–7.

101. Fragoso RP, Monteiro MBM, Lemos EM, Pereira FEL. Anti-*Toxocara* antibodies detected in children attending elementary school in Vitoria, State of Espírito Santo, Brazil: prevalence and associated factors. Rev Soc Bras Med Trop. 2011; 44(4):461–6.

102. Mattia S, Colli C, Adami C, Guilherme G, Nishi L, Rubinsky-Elefant G, et al. Seroprevalence of *Toxocara* infection in children and environmental contamination of urban areas in Paraná State, Brazil. J Helminthol. 2012; 86(4):440–5.

103. Manini MP, Marchioro AA, Colli CM, Nishi L, Falavigna-Guilherme AL. Association between contamination of public squares and seropositivity for *Toxocara* spp. in children. Vet Parasitol. 2012; 188(1-2):48–52.

104. Mendonça LR, Veiga RV, Dattoli VCC, Figueiredo CA, Fiaccone R, Santos J, et al. *Toxocara* seropositivity, atopy and wheezing in children living in poor neighbourhoods in urban Latin American. PLoS Negl Trop Dis. 2012; 6(11):e1886.

105. Guilherme EV, Marchioro AA, Araujo SM, Falavigna DLM, Adami C, Falavigna-Guilherme G, et al. Toxocariasis in children attending a public health service pneumology unit in Parana State, Brazil. Rev Inst Med Trop Sao Paulo. 2013; 55(3):189–92.

106. Negri EC, Santarém VA, Rubinsky-Elefant G, Giuffrida R. Anti-*Toxocara* spp. antibodies in an adult healthy population: serosurvey and risk factors in Southeast Brazil. Asian Pac J Trop Biomed. 2013; 3(3):211–6.

107. Prestes-Carneiro LE, Rubinsky-Elefant G, Ferreira AW, Araujo PR, Troiani C, Zago SC, et al. Seroprevalence of toxoplasmosis, toxocariasis and cysticercosis in a rural settlement, Sao Paulo State, Brazil. Pathog Glob Health. 2013; 107(2):88–95.

108. Schoenardie ER, Scaini CJ, Brod CS, Pepe MS, Villela MM, McBride AJ, et al. Seroprevalence of *Toxocara* infection in children from southern Brazil. J Parasitol. 2013; 99(3):537–9.

109. Cassenote AJ, de Abreu Lima AR, Neto JMP, Rubinsky-Elefant G. Seroprevalence and modifiable risk factors for *Toxocara* spp. in Brazilian schoolchildren. PLoS Negl Trop Dis. 2014; 8(5):e2830.

110. Martín UO, Demonte MA, Contini L, Giraldez E, Mendicino D, Del Barco M. Toxocariosis in different vulnerable groups of children in Argentina. Saludi Ciencia. 2014; 20(6):492–7.

111. Marchioro A, Colli C, Ferreira E, Viol B, Araújo S, Falavigna-Guilherme A. Risk factors associated with toxoplasmosis and toxocariasis in populations of children from nine cities in southern Brazil. J Helminthol. 2015; 89(4):428–32.

112. Oliart-Guzmán H, Delfino BM, Martins AC, Mantovani SA, Braña AM, Pereira TM, et al. Epidemiology and control of child toxocariasis in the Western Brazilian Amazon–a population-based study. Am J Trop Med Hyg. 2014; 90(4):670–81.

113. Archelli S, Santillan GI, Fonrouge R, Céspedes G, Burgos L, Radman N. Toxocariasis: seroprevalence in abandoned-institutionalized children and infants. Rev Argentina Microbiol. 2014; 46(1):3–6.

114. Araujo Z, Brandes S, Pinelli E, Bochichio MA, Palacios A, Wide A, et al. Seropositivity for ascariosis and toxocariosis and cytokine expression among the indigenous people in the Venezuelan Delta region. Rev Inst Med Trop Sao Paulo. 2015; 57(1):47–55.

115. Martínez M, García H, Figuera L, González V, Lamas F, López K, et al. Seroprevalence and risk factors of toxocariasis in preschool children in Aragua state, Venezuela. Trans R Soc Trop Med Hyg. 2015; 109(9):579–88.

116. Santos PC, Lehmann LM, Lorenzi C, Hirsch C, Telmo PL, Mattos GT, et al. The seropositivity of *Toxocara* spp. antibodies in pregnant women attented at the university hospital in Southern Brazil and the factors associated with infection. PLoS One. 2015; 10(7):e0131058.

117. Cadore PS, Zhang L, Lemos LdL, Lorenzi C, Telmo PdL, dos Santos PC, et al. Toxocariasis and childhood asthma: A case-control study. J Asthma. 2016; 53(6):601–6.

118. Cermeño J, Houda S, Salvador N, Salaverria C. Seroprevalencia y factores de riesgos asociados con la infección por *Toxocara* *canis* en la población de la laguna, estado Anzoátegui, Venezuela. Saber. 2016; 28(1):62–72.

119. Pereira LC, Elefant GR, Nóbrega YM, Vital T, Nitz N, Gandolfi L, et al. *Toxocara* spp. seroprevalence in pregnant women in Brasília, Brazil. Rev Soc Bras Med Trop. 2016; 49(5):641–3.

120. Vargas C, Torres P, Jercic MI, Lobos M, Oyarce A, Miranda JC, et al. Frequency of anti-*Toxocara* spp. antibodies in individuals attended by the Centro de Salud Familiar and environmental contamination with Toxocara canis eggs in dog feces, in the coastal Niebla town, Chile. Rev Inst Med Trop Sao Paulo. 2016; 58:62.

121. Silva MB, Amor AL, Santos LN, Galvão AA, Vera AVO, Silva ES, et al. Risk factors for *Toxocara* spp. seroprevalence and its association with atopy and asthma phenotypes in school-age children in a small town and semi-rural areas of Northeast Brazil. Acta Trop. 2017; 174:158–64.

122. Santos PC, Telmo PL, Lehmann LM, Lorenzi C, Hirsch C, Mattos GT, et al. Frequency of *Toxocara* spp. antibodies in umbilical cords of newborns attended atthe University Hospital in Southern Brazil and factors associated with infection. Acta Trop. 2017; 170:43–7.

123. Martínez M, Montero J, Pineda A, Mijares V, Lares M, Catalano E, et al. Epidemiological, clinical and laboratory features of toxocariasis in school children from Aragua State, Venezuela. Trans R Soc Trop Med Hyg. 2018; 112(6):255–63.

124. Araújo AC, Villela MM, Sena-Lopes Â, Farias NAdR, Faria LMJd, Avila LFdC, et al. Seroprevalence of *Toxoplasma gondii* and *Toxocara canis* in a human rural population of Southern Rio Grande do Sul. Rev Inst Med Trop Sao Paulo. 2018; 60:e28.

125. Berrocal J. Prevalence of *Toxocara canis* in babies and in adults as determined by the ELISA test. Trans Am Ophthalmol Soc. 1980; 78:376–413.

126. Desowitz RS, Rudoy R, Barnwell JW. Antibodies to canine helminth parasites in asthmatic and nonasthmatic children. Int Arch Allergy Immunol. 1981; 65(4):361–6.

127. Ellis Jr GS, Pakalnis VA, Worley G, Green JA, Frothingham TE, Sturner RA, et al. *Toxocara canis* infestation: clinical and epidemiological associations with seropositivity in kindergarten children. Ophthalmology. 1986; 93(8):1032–7.

128. Thompson DE, Bundy DA, Cooper ES, Schantz PM. Epidemiological characteristics of *Toxocara canis* zoonotic infection of children in a Caribbean community. Bull World Health Organ. 1986; 64(2):283–90.

129. Herrmann N, Glicman LT, Schantz PM, Weston MG, Domanski LM. Seroprevalence of zoonotic toxocariasis in the United States: 1971–1973. Am J Epidemiol. 1985; 122(5):890–6.

130. Tanner CE, Staudt M, Adamowski R, Lussier M, Bertrand S, Prichard RK. Seroepidemiological study for five different zoonotic parasites in northern Quebec. Can J Public Health. 1987; 78(4):262–6.

131. Bass JL, Mehta KA, Glickman LT, Blocker R, Eppes BM. Asymptomatic toxocariasis in children: a prospective study and treatment trial. Clin Pediatr 1987; 26(9):441–6.

132. Marmor M, Glickman L, Shofer F, Faich LA, Rosenberg C, Cornblatt B, et al. *Toxocara canis* infection of children: epidemiologic and neuropsychologic findings. Am J Public Health. 1987; 77(5):554–9.

133. Embil JA, Tanner CE, Pereira LH, Staudt M, Morrison EG, Gualazzi DA. Seroepidemiologic survey of *Toxocara canis* infection in urban and rural children. Public Health. 1988; 102(2):129–33.

134. Montalvo A, Espino A, Escalante G, Finlay C. Study of the seroprevalence of toxocariasis in an infantile population in the City of Havana. Rev Cubana Med Trop. 1994; 46(3):156–8.

135. Lévesque B, Messier V, Bonnier-Viger Y, Couillard M, Côté S, Ward BJ, et al. Seroprevalence of zoonoses in a Cree community (Canada). Diagn Microbiol Infect Dis. 2007; 59(3):283–6.

136. Jones JL, Kruszon-Moran D, Won K, Wilson M, Schantz PM. *Toxoplasma gondii* and *Toxocara* spp. co-infection. Am J Trop Med Hyg. 2008; 78(1):35–9.

137. Tinoco-Gracia L, Barreras-Serrano A, López-Valencia G, Tamayo-Sosa AR, Quiroz-Romero H, Melgarejo T. Seroprevalence of larva migrans of *Toxocara canis* and evaluation of associated risk factors among children in a Mexico-United States border region. Int J Appl Res Vet Med. 2008; 6(2):130–6.

138. Muñoz-Guzmán M, del Río-Navarro B, Valdivia-Anda G, Alba-Hurtado F. The increase in seroprevalence to *Toxocara canis* in asthmatic children is related to cross-reaction with Ascaris suum antigens. Allergol Immunopathol (Madr). 2010; 38(3):115–21.

139. Campagna S, Lévesque B, Anassour-Laouan-Sidi E, Côté S, Serhir B, Ward BJ, et al. Seroprevalence of 10 zoonotic infections in 2 Canadian Cree communities. Diagn Microbiol Infect Dis. 2011; 70(2):191–9.

140. Messier V, Levesque B, Proulx JF, Rochette L, Serhir B, Couillard M, et al. Seroprevalence of seven zoonotic infections in Nunavik, Quebec (Canada). Zoonoses Public Health. 2012; 59(2):107–17.

141. Sampasa-Kanyinga H, Lévesque B, Anassour-Laouan-Sidi E, Côté S, Serhir B, Ward BJ, et al. Zoonotic infections in native communities of James Bay, Canada. Vector Borne Zoonotic Dis. 2012; 12(6):473–81.

142. Sariego I, Kanobana K, Junco R, Vereecken K, Núñez F, Polman K, et al. Frequency of antibodies to *Toxocara* in Cuban schoolchildren. Trop Med Int Health. 2012; 17(6):711–4.

143. Alvarado-Esquivel C. Seroepidemiology of toxocariasis in a rural Tepehuanos population from Durango, Mexico. J Helminthol. 2014; 88(2):173–6.

144. Romero Núñez C, Mendoza Martínez GD, Yañez Arteaga S, Ponce Macotela M, Bustamante Montes P, Ramírez Durán N. Prevalence and risk factors associated with *Toxocara canis* infection in children. Sci World J. 2013; 2013:572089.

145. Schurer JM, Ndao M, Skinner S, Irvine J, Elmore SA, Epp T, et al. Parasitic zoonoses: one health surveillance in northern Saskatchewan. PLoS Negl Trop Dis. 2013; 7(3):e2141.

146. Kanobana K, Vereecken K, Junco Diaz R, Sariego I, Rojas L, Bonet Gorbea M, et al. *Toxocara* seropositivity, atopy and asthma: a study in C uban schoolchildren. Trop Med Int Health. 2013; 18(4):403–6.

147. Alvarado-Esquivel C, Hernández-Tinoco J, Sánchez-Anguiano LF. *Toxocara* infection in gardeners: a case control seroprevalence study. Asian Pac J Trop Med. 2014; 7(1):79–81.

148. Heredia R, Romero C, Mendoza GE, Ponce M, Portal A, González L, et al. Ocurrence of *Toxocara canis* in students of veterinary and graphic design in a mexican university. Acta Sci Vet. 2014; 42(1):1219.

149. Schurer JM, Ndao M, Quewezance H, Elmore SA, Jenkins EJ. People, pets, and parasites: one health surveillance in southeastern Saskatchewan. Am J Trop Med Hyg. 2014; 90(6):1184–90.

150. Goyette S, Cao Z, Libman M, Ndao M, Ward BJ. Seroprevalence of parasitic zoonoses and their relationship with social factors among the Canadian Inuit in Arctic regions. Diagn Microbiol Infect Dis. 2014; 78(4):404–10.

151. Cortés NN, Núñez CR, Guiliana BGL, García PAH, Cárdenas RH. Presence of anti-*Toxocara* canis antibodies and risk factors in children from the Amecameca and Chalco regions of México. BMC Pediatr. 2015; 15(1):65.

152. Ortega-Pacheco A, Torres-Acosta JF, Alzina-López A, Gutiérrez-Blanco E, Bolio-González ME, Aguilar-Caballero AJ, et al. Parasitic zoonoses in humans and their dogs from a rural community of tropical Mexico. J Trop Med. 2015; 2015:481086.

153. Cook J, Hardie R, Bailey K, Tapper M, Vickers I, Calder D, et al. Seroprevalence of human toxocariasis, Jamaica. Trop Biomed. 2016; 33(1):88–94.

154. Farmer A, Beltran T, Choi YS. Prevalence of *Toxocara* species infection in the US: Results from the National Health and Nutrition Examination Survey, 2011-2014. PLoS Negl Trop Dis. 2017; 11(7):e0005818.

155. Guo F, Forde MS, Werre SR, Krecek RC, Zhu G. Seroprevalence of five parasitic pathogens in pregnant women in ten Caribbean countries. Parasitol Res. 2017; 116(1):347–58.

156. Berrett AN, Erickson LD, Gale SD, Stone A, Brown BL, Hedges DW. *Toxocara* seroprevalence and associated risk factors in the United States. Am J Trop Med Hyg. 2017; 97(6):1846–50.

157. Josephs D, Bhinder P, Thompson A. The prevalence of *Toxocara* infection in a child population. Public Health. 1981; 95(5):273–5.

158. Stürchler D, Peter R. Parasitic disease in schoolchildren in a village in Swiss Jura. Soz Praventivmed. 1981; 26(5):317–9.

159. Ree G, Voller A, Rowland H. Toxocariasis in the British Isles 1982-3. Br Med J. 1984; 288(6417):628–9.

160. Stürchler D, Bruppacher R, Speiser F. Epidemiological aspects of toxocariasis in Switzerland. Schweiz Med Wochenschr. 1986; 116(33):1088–93.

161. Caucanas J, Magnaval J, Pascal J. Prevalence of toxocaral disease. Lancet. 1988; 331(8593):1049.

162. Kenny V, Allwright S. Seroprevalence of toxocariasis in a hospital based sample in Ireland. Ir J Med Sci. 1987; 156(12):361–3.

163. Portus M, Riera C, Prats G. A serological survey of toxocariasis in patients and healthy donors in Barcelona (Spain). Eur J Epidemiol. 1989; 5(2):224–7.

164. García LC, Alvarez AM, Martín FS. Epidemiological studies on toxocariasis and visceral larva migrans in a zone of western Spain. Anna Trop Med Parasitol. 1989; 83(6):615–20.

165. Ljungström I, van Knapen F. An epidemiological and serological study of *Toxocara* infection in Sweden. Scand J Infect Dis. 1989; 21(1):87–93.

166. Van JG, Buijs J, Van PD. Seroprevalence of *Toxocara* infection in young children in the city of The Hague. Trop Geogr Med. 1989; 41(4):294–6.

167. Genchi C, Di BS, Gatti S, Sangalli G, Scaglia M. Epidemiology of human toxocariasis in northern Italy. Parassitologia. 1990; 32(3):313–9.

168. Chobanov R, Gulieva R, Niftullaev M. A serological examination for toxocariasis in the population of greater Baku. Med Parazitol (Mosk). 1990; (3):35–8.

169. Stefancikova A, Havasiova K, Dubinský P. Serodiagnosis of larval toxocariasis in Slovakia. Bratisl Lek Listy. 1993; 94(2):99–102.

170. Havasiova K, Dubinský P, Stefancikova A. A seroepidemiological study of human *Toxocara* infection in the Slovak Republic. J Helminthol. 1993; 67(4):291–6.

171. Gueglio B, De Gentile L, Nguyen J, Achard J, Chabasse D, Marjolet M. Epidemiologic approach to human toxocariasis in western France. Parasitol Res. 1994; 80(6):531–6.

172. Buijs J, Borsboom G, van Gemund JJ, Hazebroek A, van Dongen PA, van Knapen F, et al. *Toxocara* seroprevalence in 5-year-old elementary schoolchildren: relation with allergic asthma. Am J Epidemiol. 1994; 140(9):839–47.

173. Holland C, O'lorcain P, Taylor M, Kelly A. Sero-epidemiology of toxocariasis in school children. Parasitology. 1995; 110(5):535–45.

174. Guerra A, Navarro C, de Guevara CL. Seroprevalence of toxocariasis in children and a case of VLM. Eur J Epidemiol. 1995; 11(6):701–2.

175. Gundlach J, Sadzikowski A, Tomczuk K. Occurence of antibodies against *Toxocara canis* in the sera of humans. Med Weter. 1996; 52:516–7.

176. Wolfrom E, Chêne G, Boisseau H, Beylot C, Géniaux M, Taïeb A. Chronic urticaria and *Toxocara canis*. Lancet. 1995; 345(8943):196.

177. Cilla G, Pérez-Trallero E, Gutiérrez C, Part C, Gomáriz M. Seroprevalence of *Toxocara* infection in middle-class and disadvantaged children in northern Spain (Gipuzkoa, Basque Country). Eur J Epidemiol. 1996; 12(5):541–3.

178. Fenoy S, Cuellar C, Guillen J. Seroprevalence of toxocariasis in children and adults in Madrid and Tenerife, Spain. J Helminthol. 1996; 70(2):109–13.

179. Buijs J, Borsboom G, Renting M, Hilgersom W, van Wieringen JC, Jansen G, et al. Relationship between allergic manifestations and *Toxocara* seropositivity: a cross-sectional study among elementary school children. Eur Respir J. 1997; 10(7):1467–75.

180. Jimenez J, Valladares B, Fernandez-Palacios J, De Armas F, Del Castillo A. A serologic study of human toxocariasis in the Canary Islands (Spain): environmental influences. Am J Trop Med Hyg. 1997; 56(1):113–5.

181. Vassalou H, Kordatos E, Platis N, Vakalis N. Seroprevalence study of toxocariasis in a district of West Attiki. Delt Hell Mikrobiol Hygieinol Hetair. 1998; 43:258–62.

182. Humbert P, Niezborala M, Salembier R, Aubin F, Piarroux R, Buchet S, et al. Skin manifestations associated with toxocariasis: a case-control study. Dermatology. 2000; 201(3):230–4.

183. Giacometti A, Cirioni O, Fortuna M, Osimani P, Antonicelli L, Del Prete M, et al. Environmental and serological evidence for the presence of toxocariasis in the urban area of Ancona, Italy. Eur J Epidemiol. 2000; 16(11):1023–6.

184. Hermanowska-Szpakowicz T, Kondrusik M, Swierzbińska R, Zajkowska J, Pancewicz S. Incidence of antibody detection against *Toxocara canis* and clinical symptoms in inhabitants of North-Eastern Poland. Pol Merkur Lekarski. 2001; 10(57):168–70.

185. Theodoridis I, Frydas S, Papazahariadou M, Hatzistilianou M, Adamama-Moraitou K, Di Gioacchino M, et al. Toxocarosis as Zoonosis. A Review of Literature and the Prevalence of *Toxocara canis* Antibodies in 511 Serum Sample. Int J Immunopathol Pharmacol. 2001; 14(1):17–23.

186. Oshevskaia Z, Derzhavina T, Terina G, Mikholap L, Liapina N, Anisimkina V, et al. Toxocariasis in the Tula region. Med Parazitol (Mosk). 2003; (1):30–3.

187. Habluetzel A, Traldi G, Ruggieri S, Attili A, Scuppa P, Marchetti R, et al. An estimation of *Toxocara canis* prevalence in dogs, environmental egg contamination and risk of human infection in the Marche region of Italy. Vet Parasitol. 2003; 113(3-4):243–52.

188. Juncker-Voss M, Prosl H, Lussy H, Enzenberg U, Auer H, Lassnig H, et al. Screening for antibodies against zoonotic agents among employees of the Zoological Garden of Vienna, Schönbrunn, Austria. Berl Munch Tierarztl Wochenschr. 2004; 117(9-10):404–9.

189. Deutz A, Fuchs K, Auer H, Kerbl U, Aspöck H, Köfer J. *Toxocara*-infestations in Austria: a study on the risk of infection of farmers, slaughterhouse staff, hunters and veterinarians. Parasitol Res. 2005; 97(5):390–4.

190. Balicka-Ramisz A, Horodnicka-Jozwa A, Ramisz A, Laurans L, Wnuk W, Pilarczyk B. Toxocariasis in dogs and occurrence of antibodies against *Toxocara canis* in children. Med Wete. 2005; 61(7):786–8.

191. Gonzalez-Quintela A, Gude F, Campos J, Garea M, Romero P, Rey J, et al. *Toxocara* infection seroprevalence and its relationship with atopic features in a general adult population. Int Arch Allergy Immunol. 2006; 139(4):317–24.

192. Yariktas M, Demirci M, Aynali G, Kaya S, Doner F. Relationship between *Toxocara* seropositivity and allergic rhinitis. Am J Rhinol. 2007; 21(2):248–50.

193. Akyol A, Bicerol B, Ertug S, Ertabaklar H, Kiylioglu N. Epilepsy and seropositivity rates of *Toxocara canis* and *Toxoplasma* *gondii*. Seizure. 2007; 16(3):233–7.

194. Dogan N, Dinleyici EÇ, Bor Ö, Toz SÖ, Özbel Y. Seroepidemiological survey for *Toxocara canis* infection in the northwestern part of Turkey. Turkiye Parazitol Derg. 2007; 31(4):288–91.

195. Nicoletti A, Sofia V, Mantella A, Vitale G, Contrafatto D, Sorbello V, et al. Epilepsy and toxocariasis: a case–control study in Italy. Epilepsia. 2008; 49(4):594–9.

196. Zarnowska H, Borecka A, Gawor J, Marczyńska M, Dobosz S, Basiak W. A serological and epidemiological evaluation of risk factors for toxocariasis in children in central Poland. J Helminthol. 2008; 82(2):123–7.

197. Jarosz W, Mizgajska-Wiktor H, Kirwan P, Konarski J, Rychlicki W, Wawrzyniak G. Developmental age, physical fitness and *Toxocara* seroprevalence amongst lower-secondary students living in rural areas contaminated with *Toxocara* eggs. Parasitology. 2010; 137(1):53–63.

198. Sviben M, Čavlek T, Missoni E, Galinović G. Seroprevalence of *Toxocara canis* infection among asymptomatic children with eosinophilia in Croatia. J Helminthol. 2009; 83(4):369–71.

199. Stensvold CR, Skov J, Møller LN, Jensen PM, Kapel CM, Petersen E, et al. Seroprevalence of human toxocariasis in Denmark. Clin Vaccine Immunol. 2009; 16(9):1372–3.

200. Torgerson PR, Rosenheim K, Tanner I, Ziadinov I, Grimm F, Brunner M, et al. Echinococcosis, toxocarosis and toxoplasmosis screening in a rural community in eastern Kazakhstan. Trop Med Int Health. 2009; 14(3):341–8.

201. Bellanger AP, Humbert P, Gavignet B, Deschaseaux A, Barisien C, Roussel S, et al. Comparative assessment of enzyme‐linked immunosorbent assay and Western blot for the diagnosis of toxocariasis in patients with skin disorders. Br J Dermatol. 2010; 162(1):80–2.

202. Demirci M, Kaya S, Çetin E, Arıdoğan B, Önal S, Korkmaz M. Seroepidemiological investigation of toxocariasis in the isparta region of Turkey. Iranian J Parasitol. 2010; 5(2):52–9.

203. Magnaval J-F, Tolou H, Gibert M, Innokentiev V, Laborde M, Melnichuk O, et al. Seroepidemiology of nine zoonoses in Viljujsk, republic of sakha (northeastern siberia, Russian federation). Vector Borne Zoonotic Dis. 2011; 11(2):157–60.

204. Cojocariu I, Bahnea R, Luca C, Leca D, Luca M. Adult toxocariasis. Rev Med Chir Soc Med Nat Iasi. 2012; 116(2):432–5.

205. Cobzaru R-G, Rîpă C, Leon MM, Luca MC, Ivan A, Luca M. Correlation between asthma and *Toxocara canis* infection. Rev Med Chir Soc Med Nat Iasi. 2012; 116(3):727–30.

206. Çelik T, Kaplan Y, Ataş E, Öztuna D, Berilgen S. *Toxocara* seroprevalence in patients with idiopathic Parkinson's disease: chance association or coincidence? Biomed Res Int. 2013; 2013:685196.

207. Poeppl W, Herkner H, Tobudic S, Faas A, Mooseder G, Burgmann H, et al. Exposure to *Echinococcus multilocularis*, *Toxocara canis*, and *Toxocara cati* in Austria: a nationwide cross-sectional seroprevalence study. Vector Borne Zoonotic Dis. 2013; 13(11):798–803.

208. Kaneva E, Rainova I, Harizanov R, Nikolov G, Kaftandjiev I, Mineva I. Study of *Toxocara* seroprevalence among patients with allergy and healthy individuals in Bulgaria. Parasite Immunol. 2015; 37(10):505–9.

209. Van Den Broucke S, Kanobana K, Polman K, Soentjens P, Vekemans M, Theunissen C, et al. Toxocariasis diagnosed in international travelers at the Institute of Tropical Medicine, Antwerp, Belgium, from 2000 to 2013. PLoS Negl Trop Dis. 2015; 9(3):e0003559.

210. Burak-Selek M, Baylan O, Kutlu A, Özyurt M. *Toxocara canis* IgG seropositivity in patients with chronic urticaria. Iran J Allergy Asthma Immunol. 2015; 14(4):450–6.

211. Sozen H, Citil BE, Caylak S, Gokmen AA, Kaya S, Demirci M, et al. Seroepidemiological study of toxocariasis among volunteers animal husbandry workers and veterinary in Southern Anatolia in Turkey in 2014. Iran J Parasitol. 2015; 10(3):473–81.

212. Antolová D, Jarčuška P, Janičko M, Madarasová-Gecková A, Halánová M, Čisláková L, et al. Seroprevalence of human *Toxocara* infections in the Roma and non-Roma populations of Eastern Slovakia: a cross-sectional study. Epidemiol Infect. 2015; 143(10):2249–58.

213. Magnaval J-F, Leparc-Goffart I, Gibert M, Gurieva A, Outreville J, Dyachkovskaya P, et al. A serological survey about zoonoses in the verkhoyansk area, northeastern siberia (sakha republic, Russian federation). Vector Borne Zoonotic Dis. 2016; 16(2):103–9.

214. Gabrielli S, Tasić-Otašević S, Ignjatović A, Fraulo M, Trenkić-Božinović M, Momčilović S, et al. Seroprevalence and risk factors for *Toxocara canis* infection in Serbia during 2015. Foodborne Pathog Dis. 2017; 14(1):43–9.

215. Lassen B, Janson M, Viltrop A, Neare K, Hütt P, Golovljova I, et al. Serological evidence of exposure to globally relevant zoonotic parasites in the Estonian population. PLoS One. 2016; 11(10):e0164142.

216. Mughini-Gras L, Harms M, van Pelt W, Pinelli E, Kortbeek T. Seroepidemiology of human *Toxocara* and *Ascaris* infections in the Netherlands. Parasitol Res. 2016; 115(10):3779–94.

217. Kroten A, Toczylowski K, Kiziewicz B, Oldak E, Sulik A. Environmental contamination with *Toxocara* eggs and seroprevalence of toxocariasis in children of northeastern Poland. Parasitol Res. 2016; 115(1):205–9.

218. Martelli G, Di Girolamo C, Zammarchi L, Angheben A, Morandi M, Tais S, et al. Seroprevalence of five neglected parasitic diseases among immigrants accessing five infectious and tropical diseases units in Italy: a cross-sectional study. Clin Microbiol Infect. 2017; 23(5):335. e1–e5.

219. Papavasilopoulos V, Pitiriga V, Birbas K, Elefsiniotis J, Bonatsos G, Tsakris A. Soil contamination by *Toxocara canis* and human seroprevalence in the Attica region, Greece. Germs. 2018; 8(3):155–61.

220. Overbosch FW, van Gool T, Matser A, Sonder GJ. Low incidence of helminth infections (schistosomiasis, strongyloidiasis, filariasis, toxocariasis) among Dutch long-term travelers: A prospective study, 2008-2011. PLoS One. 2018; 13(5):e0197770.

221. Abo-Shehada M, Sharif L, El-Sukhon S, Abuharfeil N, Atmeh R. Seroprevalence of *Toxocara canis* antibodies in humans in northern Jordan. J Helminthol. 1992; 66(1):75–8.

222. Chantal J, Bessiere M, LeGuenno B, Magnaval J, Dorchies P. A sero-prevalence survey of carriers of the agents of zoonotic diseases on some workers of Djibouti slaughter-house. Bull Soc Pathol Exot. 1996; 89(5):353–7.

223. Sadjjadi S, Khosravi M, Mehrabani D, Oryan A. Seroprevalence of *Toxocara* infection in school children in Shiraz, Southern Iran. J Trop Pediatr. 2000; 46(6):327–30.

224. Akhlaghi L, Ourmazdi H, Sarafnia A, Vaziri S, Jadidian K, Leghaii Z. An investigation on the toxocariasis seroprevalence in children (2-12 years old) from Mahidasht area of Kermanshah province (2003-2004). Razi J Med Sci. 2006; 13(52):41–8.

225. Kanafani Z, Skoury A, Araj G, El-Khoury M, Sawaya R, Atweh S, et al. Seroprevalence of toxocariasis in Lebanon: a pilot study. Parasitology. 2006; 132(5):635–9.

226. Fallah M, Azimi A, Taherkhani H. Seroprevalence of toxocariasis in children aged 1-9 years in western Islamic Republic of Iran, 2003. East Mediterr Health J. 2007; 13(5):1073–7.

227. Nourian A, Amiri M, Ataeian A, Haniloo A, Mosavinasab S, Badali H. Seroepidemiological study for toxocariasis among children in Zanjan-northwest of Iran. Pak J Biol Sci. 2008; 11(14):1844–7.

228. Sharif M, Daryani A, Barzegar G, Nasrolahei M, Khalilian A. Seroprevalence of toxocariasis in schoolchildren in Northern Iran. Pak J Biol Sci. 2010; 13(4):180–4.

229. Alavi S, Hosseini S, Rahdar M, Salmanzadeh S, Nikkhuy A. Determination of seroprevalence rate of *Toxocara canis* in 6-15 years aged rural and urban school children in Ahvaz, Iran. Jundishapur Sci Med J. 2011; 10(3):240–8.

230. El-Shazly A, Abdel Baset S, Kamal A, Mohammed KA, Sakrs T, Hammad S. Seroprevalence of human toxocariasis (visceral larva migrans). J Egypt Soc Parasitol. 2013; 39(3):731–44.

231. El-Tantawy NL, El-Nahas HA, Salem D, Salem N, Hasaneen BM. Seroprevalence of *Toxoplasma gondii* and *Toxocara* spp. in children with cryptogenic epilepsy. Am J Infect Dis Microbiol. 2013; 1(5):92–5.

232. Hosseini-Safa A, Mousavi SM, Badorani MBB, Samani MG, Mostafaei S, Darani HY. Seroepidemiology of toxocariasis in children (5–15 yr old) referred to the pediatric clinic of Imam Hossein Hospital, Isfahan, Iran. Iran J Parasitol. 2015; 10(4):632–7.

233. Awadallah MA, Salem LM. Zoonotic enteric parasites transmitted from dogs in Egypt with special concern to Toxocara canis infection. Vet World. 2015; 8(8):946–957.

234. Sarkari B, Lari M, Shafiei R, Sadjjadi SM. A comparative seroprevalence study of toxocariasis in hypereosinophilic and apparently healthy individuals. Arch Pediatr Infect Dis. 2014; 3(4):e17911.

235. Allahdin S, Khademvatan S, Rafiei A, Momen A, Rafiei R. Frequency of *Toxoplasma* and *Toxocara* sp. antibodies in epileptic patients, in south western Iran. Iran J Child Neurol. 2015; 9(4):32–40.

236. Mosayebi M, Moini L, Hajihossein R, Didehdar M, Eslamirad Z. Detection of specific antibody reactivity to *Toxocara* larval excretory-secretory antigens in asthmatic patients (5-15 years). Open Microbiol J. 2016; 10:162-7.

237. Momeni T, Mahami-Oskouei M, Fallah E, Safaiyan A, Mahami-Oskouei L. Latent and asymptomatic *Toxocara* infection among young population in Northwest Iran: The necessity of informing people as a potential health risk. Scientifica. 2016; 2016:3562056.

238. Galal LA, Mahmoud AE, Attia RA, Eltayeb A, Mahran DG. Socio-demographic, clinical and laboratory predictors for the diagnosis of visceral larva migrans in children-upper Egypt. Int J Trop Dis Health. 2016; 19:1–13.

239. Berenji F, Pouryousef A, Abdolmajid F, Mahmoudi M, Salehi M, Khoshnegah J. Seroepidemiological study of toxocariasis in the owners of domestic cats and dogs in Mashhad, Northeastern Iran. Iran J Parasitol. 2016; 11(2):265–268.

240. Abbasi S, Ahmadi Afshar A, Haniloo A. Prevalence of anti-*Toxocara* antibodies in children with allergic manifestations referring to allergy clinics in Zanjan. Sci J Kurdistan Univ Med Sci. 2017; 22(2):71–9.

241. Shahraki Khoshsima M, Dabirzadeh M, Afshari M, Maroufi Y. Epidemiological study of *Toxocara canis* in children under 14-years-old and dogs in Zabol and Chabahar Districts, southeast of Iran. Iran J Parasitol. 2017; 12(1):101–107.

242. Beiromvand M, Rafiei A, Mirzavand S, Rahdar M, Haddad FM. Screening of cystic echinococcosis and toxocariasis in rural inhabitants of Khuzestan Province, southwest Iran. Trop Biomed. 2018; 35(1):32–40.

243. Khozime A, Mirsadraee M, Borji H. *Toxocara* sero-prevalence and its relationship with allergic asthma in asthmatic patients in north-eastern Iran. J Helminthol. 2018:1-4. [Epub ahead of print].

244. Baghani Z, Khazan H, Sohrabi MR, Rostami A. Seroprevalence of *Toxocara* infection among healthy individuals referred to the medical center laboratories in Tehran City, Capital of Iran. Novelty Biomed. 2018; 6(2):68–73.

245. Mahmoudvand H, Taee N, Ebrahimzadeh F, Mirhosseini MS, Faraji M. Seroprevalence and Risk Factors of *Toxocara canis* Infection in Children (2-15 Years Old) Referred to Health Centers of Lorestan Province, Iran. J Pediatr Infect Dis. 2018; 13(1):20–4.

246. Aghamolaie S, Seyyedtabaei SJ, Behniafar H, Foroutan M, Saber V, Hanifehpur H, et al. Seroepidemiology, modifiable risk factors and clinical symptoms of *Toxocara* spp. infection in northern Iran. Trans R Soc Trop Med Hyg. 2018; 113(3):116–22.

247. Shokouhi S, Abdi J. Seroprevalence of *Toxocara* in Children from Urban and Rural Areas of Ilam Province, West Iran. Osong Public Health Res Perspect. 2018; 9(3):101–4.

248. Sarkari B, Alirezaei R, Layegh Gigloo A, Rezaei Z, Mikaeili F, Bahreini MS, et al. Seroprevalence and risk factors for *Toxocara* infection among children in a rural community in Fars province, southern Iran. Parasite Immunol. 2018; 40(11):e12582.

249. Raissi V, Sohrabi Z, Getso M, Raiesi O, Hafshejani SH, Shabandoust H, et al. Risk factors and prevalence of toxocariasis in pregnant women and diabetic patients compared to healthy adults in Ilam province, western Iran. Excli J. 2018; 17:983–8.

250. Khoshsima-Shahraki M, Dabirzadeh M, Azizi H, Khedri J, Djahed B, Asghar Neshat A. Seroepidemiology of *Toxocara canis* in children under 14 years referring to laboratories of Sistan and Baluchestan province in Southeast of Iran. Iran J Parasitol. 2019; 14(1):89–94.
