# Supplementary material for: Seroprevalence estimates for toxocariasis in people worldwide: A systematic review and meta-analysis
Source: PLoS Negl Trop Dis. 2019 Dec 19;13(12):e0007809. doi: 10.1371/journal.pntd.0007809 (PMC6922318; doi:10.1371/journal.pntd.0007809)
Supplement: S2 Table — (DOCX) [file pntd.0007809.s003.docx]

**S2 Table.** Global, regional and national pooled *T*-seroprevalence among healthy population (results from 250 studies performed in 71 countries).

| WHO-regions/ country | Number of datasets | Number of seropositive samples/total number of samples tested | Pooled seroprevalence (%) (meta-analysis)  [95% CI] | Heterogeneity | |
| --- | --- | --- | --- | --- | --- |
|  |  |  |  | χ^2^ | I^2^ (%) |
| Global | **253** | **62,927/265,327** | **19.0 (16.6**–**21.4)** | **57983.1** | **99.6** |
| African region | **19** | **2,339/6,360** | **37.7 (25.7**–**50.6)** | **1920.3** | **99.1** |
| Nigeria | 5 | 558/1,090 | 44.3 (18.7–71.8) | 336.8 | 98.8 |
| Sub-Saharan Africa (Ghana, Uganda, Tanzania, South Africa, Kenya) | 3 | 533/2,242 | 23.9 (21.6–26.3) | 3.3 | 39.9 |
| Uganda | 2 | 50/203 | 24.2 (18.5–30.4) | 3.3 | 70.0 |
| La Reunion | 1 | 359/387 | 92.8 (89.7–95.1) | NA | NA |
| Kenya | 1 | 17/228 | 7.5 (4.4–11.7) | NA | NA |
| Burundi | 1 | 97/191 | 50.8 (43.5–58.1) | NA | NA |
| Swaziland | 1 | 41/92 | 44.6 (34.2–55.3) | NA | NA |
| Cameroon | 1 | 61/168 | 36.3 (29.0–44.1) | NA | NA |
| South Africa | 1 | 77/260 | 29.6 (24.1–35.6) | NA | NA |
| Mozambique | 1 | 44/601 | 7.3 (5.4–9.7) | NA | NA |
| Ghana | 1 | 303/566 | 53.5 (49.3–57.7) | NA | NA |
| Gabon | 1 | 199/332 | 59.9 (54.4–65.3) | NA | NA |
| South-East Asian Region | **12** | **1,193/3,092** | **34.1 (20.2**–**49.4)** | **784.0** | **98.6** |
| India | 5 | 184/823 | 19.6 (9.6–32.1) | 65.5 | 93.9 |
| Indonesia | 3 | 373/592 | 63.0 (59.1–66.9) | 0.0 | 0.0 |
| Sri Lanka | 2 | 451/1116 | 39.9 (37.1–42.8) | 0.0 | 0.0 |
| Nepal | 1 | 162/200 | 81.0 (74.9–86.2) | NA | NA |
| Thailand | 1 | 23/361 | 6.4 (4.1–9.4) | NA | NA |
| Western Pacific Region | **24** | **37,662/89,997** | **24.2 (16.0**–**33.5)** | **6030.8** | **99.6** |
| South Korea | 5 | 564/2,016 | 24.2 (5.2–51.4) | 644.5 | 99.4 |
| China | 4 | 991/6,685 | 14.9 (11.7–18.4) | 42.1 | 99.2 |
| Malaysia | 4 | 232/1,057 | 15.1 (5.0–29.3) | 83.6 | 96.4 |
| Taiwan | 4 | 558/1,142 | 45.6 (17.3–75.5) | 300.1 | 99.0 |
| Japan | 2 | 23/613 | 3.6 (2.2–5.3) | 300.1 | 99.7 |
| Philippines | 1 | 108/162 | 66.7 (58.8–73.9) | NA | NA |
| Marshall Islands | 1 | 144/166 | 86.7 (80.6–91.5) | NA | NA |
| Vietnam | 1 | 34,995/77,356 | 45.2 (44.9–45.6) | NA | NA |
| Australia | 1 | 46/660 | 7.0 (5.1–9.2) | NA | NA |
| New Zealand | 1 | 1/140 | 0.7 (0.0–3.9 | NA | NA |
| Region of the Americas | **104** | **13,524/87,173** | **22.8 (19.7**–**26.0)** | **11780.8** | **99.1** |
| South America | **72** | **6,933/23,169** | **27.8 (23.1**–**32.7)** | **4780.0** | **98.5** |
| Brazil | 40 | 4,026/13,970 | 27.6 (21.1–34.6) | 3148.9 | 98.8 |
| Argentina | 12 | 1,049/2,575 | 35.4 (26.3–45.0) | 253.3 | 95.7 |
| Venezuela | 8 | 311/1,706 | 18.0 (13.0–23.6) | 51.7 | 86.5 |
| Peru | 6 | 619/2,710 | 27.5 (14.8–42.3) | 313.9 | 98.4 |
| Bolivia | 2 | 101/449 | 21.5 (17.8–25.4) | 313.9 | 99.7 |
| Chile | 2 | 100/543 | 17.1 (14.0–20.4) | 313.9 | 99.7 |
| Colombia | 1 | 98/207 | 47.3 (40.4–54.4) | NA | NA |
| Trinidad and Tobago | 1 | 629/1,009 | 62.3 (59.3–65.3) | NA | NA |
| North America | **32** | **6,591/64,004** | **12.8 (10.0**–**15.8)** | **3002.3** | **99.0** |
| Canada | 9 | 551/7,585 | 6.2 (2.8–10.6) | 322.1 | 97.5 |
| Unites State | 8 | 4,569/50,263 | 9.2 (6.1–12.8) | 944.7 | 99.3 |
| Mexico | 8 | 183/1,329 | 14.6 (8.4–22.1) | 87.8 | 92.0 |
| Cuba | 3 | 784/2,125 | 26.0 (12.3–42.7) | 114.7 | 98.3 |
| Puerto Rico | 1 | 43/641 | 6.7 (4.9–8.9) | NA | NA |
| St. Lucia | 1 | 71/82 | 86.6 (77.3–93.1) | NA | NA |
| Caribbean Countries | 1 | 63/435 | 14.5 (11.3–18.1) | NA | NA |
| Jamaica | 1 | 327/1,544 | 21.2 (19.2–23.3) | NA | NA |
| Europe region | **64** | **7,024/67,610** | **10.5 (8.5**–**12.8)** | **4861.6** | **98.7** |
| Spain | 7 | 869/18,264 | 4.3 (3.2–12.9) | 435.2 | 98.6 |
| Turkey | 7 | 212/1,836 | 6.7 (2.9–11.9) | 69.7 | 91.4 |
| Poland | 6 | 422/2,280 | 15.9 (10.1–22.7) | 76.3 | 93.5 |
| Italy | 5 | 122/3,017 | 4.4 (2.5–6.8) | 17.9 | 77.6 |
| Netherlands | 5 | 585/7,604 | 5.8 (2.6–10.2) | 139.1 | 97.1 |
| France | 5 | 500/2,486 | 17.5 (11.0–25.1) | 47.6 | 91.6 |
| Slovakia | 3 | 833/5,173 | 14.5 (11.0–18.4) | 21.7 | 90.8 |
| Greece | 3 | 113/1,102 | 9.4 (2.8–19.3) | 44.1 | 95.5 |
| Russia | 3 | 648/2,753 | 6.8 (0.1–29.7) | 93.8 | 97.9 |
| Austria | 3 | 80/1,156 | 8.4 (1.7–18.9) | 14.5 | 86.2 |
| Ireland | 2 | 717/2,431 | 29.4 (27.6–31.2) | 14.5 | 93.1 |
| Switzerland | 2 | 44/899 | 4.8 (3.5–6.3) | 14.5 | 93.1 |
| Romania | 2 | 240/545 | 42.3 (38.2–46.5) | 14.5 | 93.1 |
| United Kingdom | 2 | 363/1,538 | 23.5 (21.4–25.6) | NA | NA |
| Sweden | 1 | 43/175 | 24.6 (18.4–31.6) | NA | NA |
| Azerbaijan | 1 | 320/4,765 | 6.7 (6.0–7.5) | NA | NA |
| Croatia | 1 | 46/142 | 32.4 (24.8–40.8) | NA | NA |
| Denmark | 1 | 87/3,247 | 2.7 (2.2–3.3) | NA | NA |
| Kazakhstan | 1 | 349/3,126 | 11.2 (10.1–12.3) | NA | NA |
| Bulgaria | 1 | 2/50 | 4.0 (0.5–13.7) | NA | NA |
| Belgium | 1 | 190/3,436 | 5.5 (4.8–6.3) | NA | NA |
| Serbia | 1 | 76/338 | 22.5 (18.1–27.3) | NA | NA |
| Estonia | 1 | 163/1,247 | 13.1 (11.2–15.1) | NA | NA |
| Middle East and north Africa | **30** | **1,185/11,095** | **8.2 (5.1**–**12.0)** | **1202.4** | **97.6** |
| Iran | 23 | 933/9,392 | 5.7 (2.9–9.4) | 959.8 | 97.7 |
| Egypt | 4 | 148/746 | 32.3 (9.9–60.1) | 137.8 | 97.6 |
| Jordan | 1 | 76/699 | 10.9 (8.7–13.4) | NA | NA |
| Lebanon | 1 | 28/150 | 18.7 (12.8–25.8) | NA | NA |
| Djibouti | 1 | 0/108 | 0.1 (0.0–3.4) | NA | NA |

**Abbreviations:** NA, not applicable

WHO regions are sorted according to *T*-seroprevalence rates

Countries are sorted according to number of studies included
