# Supplementary material for: Seroprevalence estimates for toxocariasis in people worldwide: A systematic review and meta-analysis
Source: PLoS Negl Trop Dis. 2019 Dec 19;13(12):e0007809. doi: 10.1371/journal.pntd.0007809 (PMC6922318; doi:10.1371/journal.pntd.0007809)
Supplement: S3 Table — (DOCX) [file pntd.0007809.s004.docx]

**S3 Table.** *T*-seroprevalence worldwide according to *a priori* defined stubgroups

| Parameters/subgroups | Number of datasets | Number of seropositive samples/total number of samples tested | Pooled seroprevalence (%)  (meta-analysis)  [95% CI] | Heterogeneity | |
| --- | --- | --- | --- | --- | --- |
|  |  |  |  | χ^2^ | I^2^ (%) |
| Type of participants |  |  |  |  |  |
| Children | 109 | 10,838/46,026 | 22.7 (19.1–26.5) | 9746.4 | 98.9 |
| Adult | 51 | 3,362/24,191 | 12.8 (8.4–17.9) | 5962.0 | 99.2 |
| Children and adult | 93 | 48,727/195,110 | 18.3 (14.5–22.5) | 40146.7 | 99.8 |
| Type of diagnosis method** |  |  |  |  |  |
| ELISA | 243 | 62,156/263,235 | 18.6 (16.3–21.1) | 56573.6 | 99.6 |
| Western blot | 10 | 771/2,092 | 27.8 (8.7–52.5) | 1225.3 | 99.3 |
| Type of study design |  |  |  |  |  |
| Cross-sectional | 219 | 61,844/259,233 | 20.2 (17.6–22.9) | 57104.7 | 99.6 |
| Case-control^*^ | 34 | 1,083/6,094 | 11.8 (8.0–16.2) | 770.7 | 99.6 |
| Sample size |  |  |  |  |  |
| <500 | 178 | 9,446/37,796 | 20.6 (17.5–23.9) | 10464.1 | 98.3 |
| 500-1000 | 38 | 4,999/25,553 | 16.6 (11.6–22.2) | 5037.1 | 99.3 |
| 1000-5000 | 32 | 9,147/66,884 | 14.5 (11.0–18.3) | 5807.5 | 99.5 |
| >5000 | 5 | 39,335/135,094 | 12.2 (0.9–33.8) | 30586.2 | 99.5 |
| Implementation Year |  |  |  |  |  |
| 1971-1999 | 84 | 12,179/89,086 | 18.6 (15.6–21.7) | 10889.4 | 99.2 |
| 2000-2004 | 39 | 3,964/21,505 | 23.6 (18.3–29.4) | 3339.1 | 98.9 |
| 2005-2009 | 53 | 3,671/19,951 | 19.4 (14.9–24.2) | 3534.0 | 98.5 |
| 2010-2014 | 65 | 42,463/130,007 | 17.6 (12.5–23.4) | 27505.7 | 99.8 |
| 2015-2017 | 12 | 650/4,778 | 12.9 (6.9–20.4) | 523.9 | 97.9 |

**Abbreviations:** E**LI**SA; enzyme-linked immunosorbent assay

* In case control studies, data were extracted only from healthy people from general population

** Only studies that evaluated both methods were included in this subgroup analysis
