# Supplementary figures and images for: Seroprevalence estimates for toxocariasis in people worldwide: A systematic review and meta-analysis
Source: PLoS Negl Trop Dis. 2019 Dec 19;13(12):e0007809. doi: 10.1371/journal.pntd.0007809 (PMC6922318; doi:10.1371/journal.pntd.0007809)

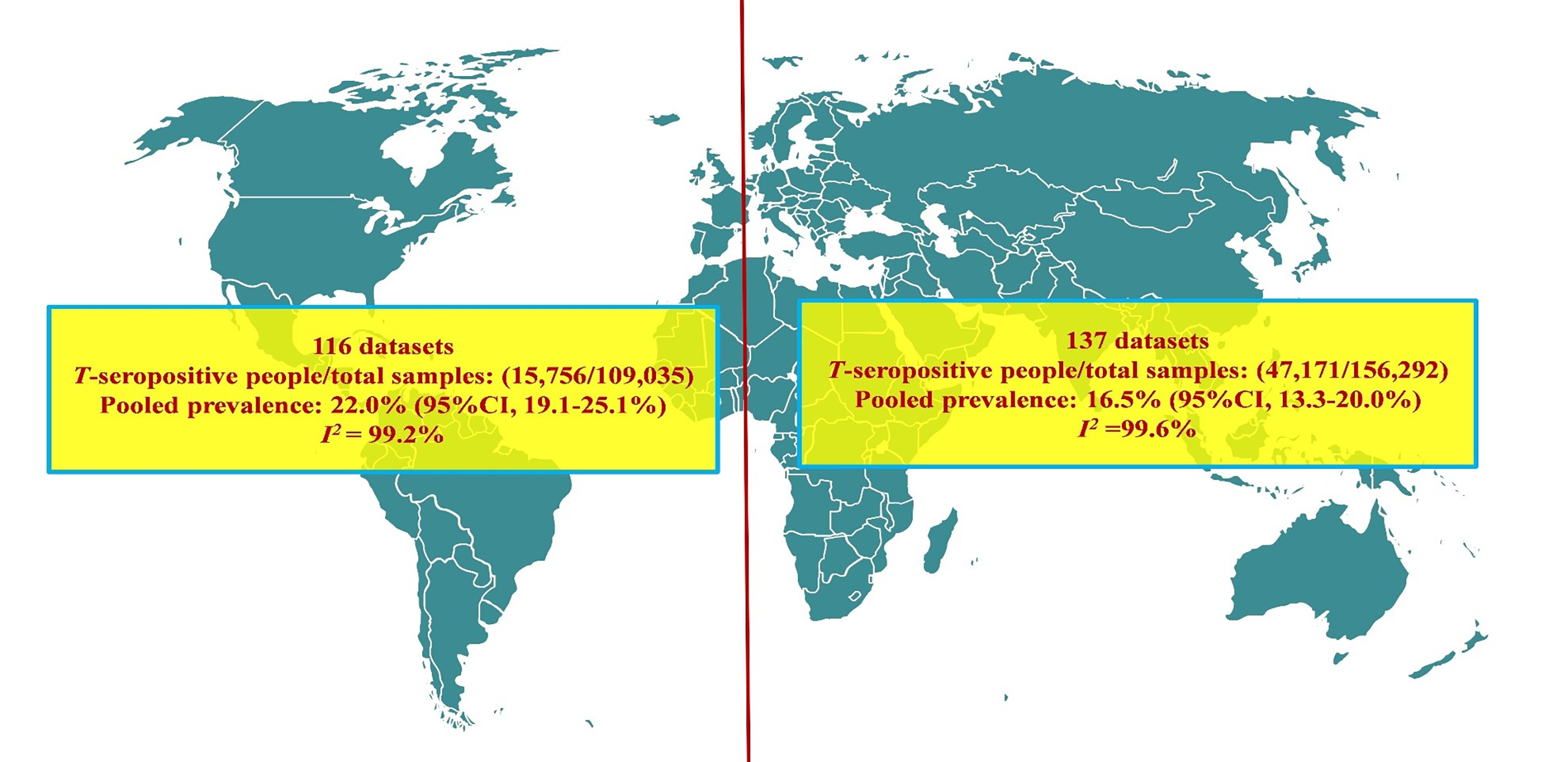

Supplement: S1 Fig — Abbreviations: CI, confidence interval; T-seropositive, number of seropositive people for toxocariasis (TIF) [file pntd.0007809.s005.tif]

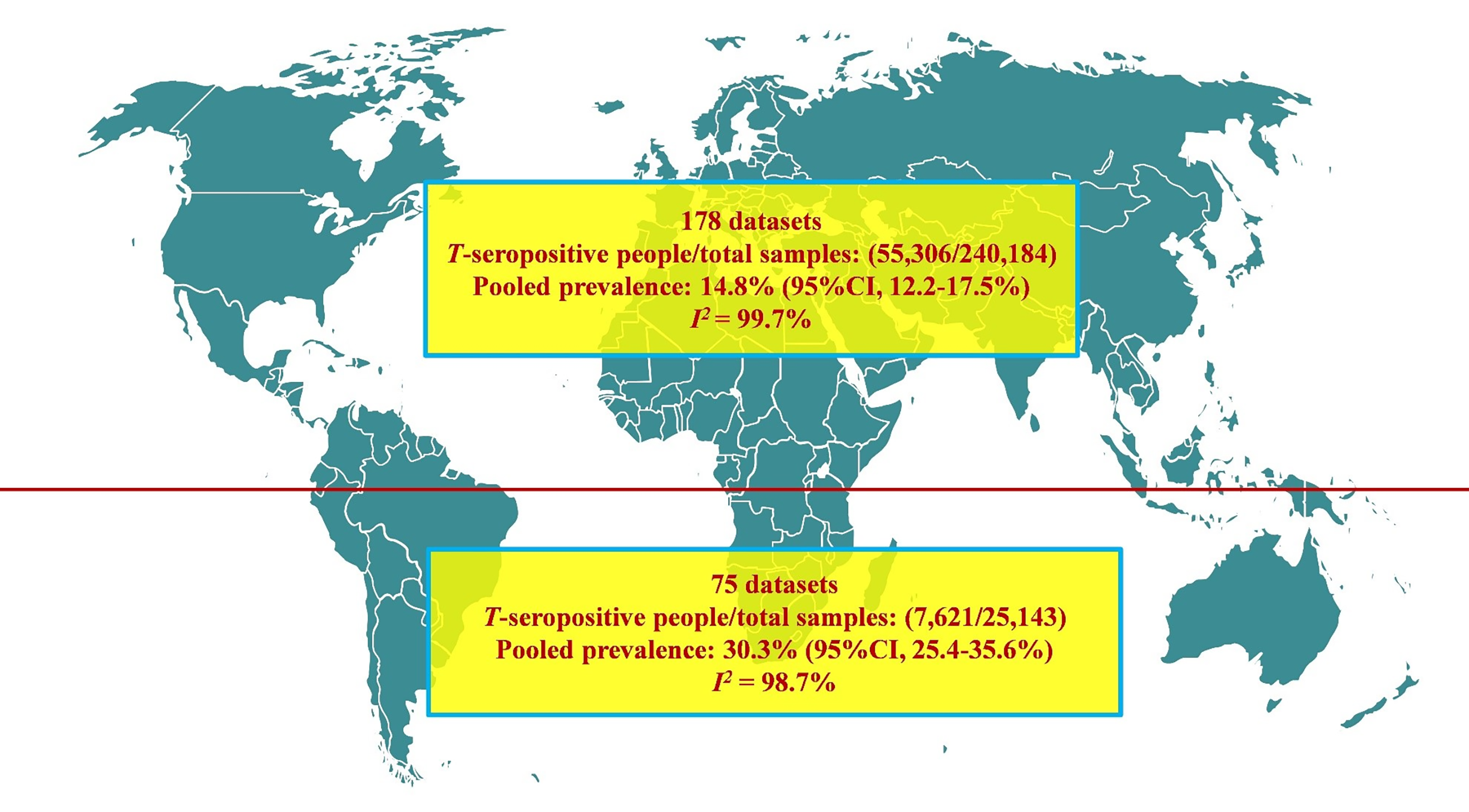

Supplement: S2 Fig — Abbreviations: CI, confidence interval; T-seropositive, number of seropositive people for toxocariasis. (TIF) [file pntd.0007809.s006.tif]

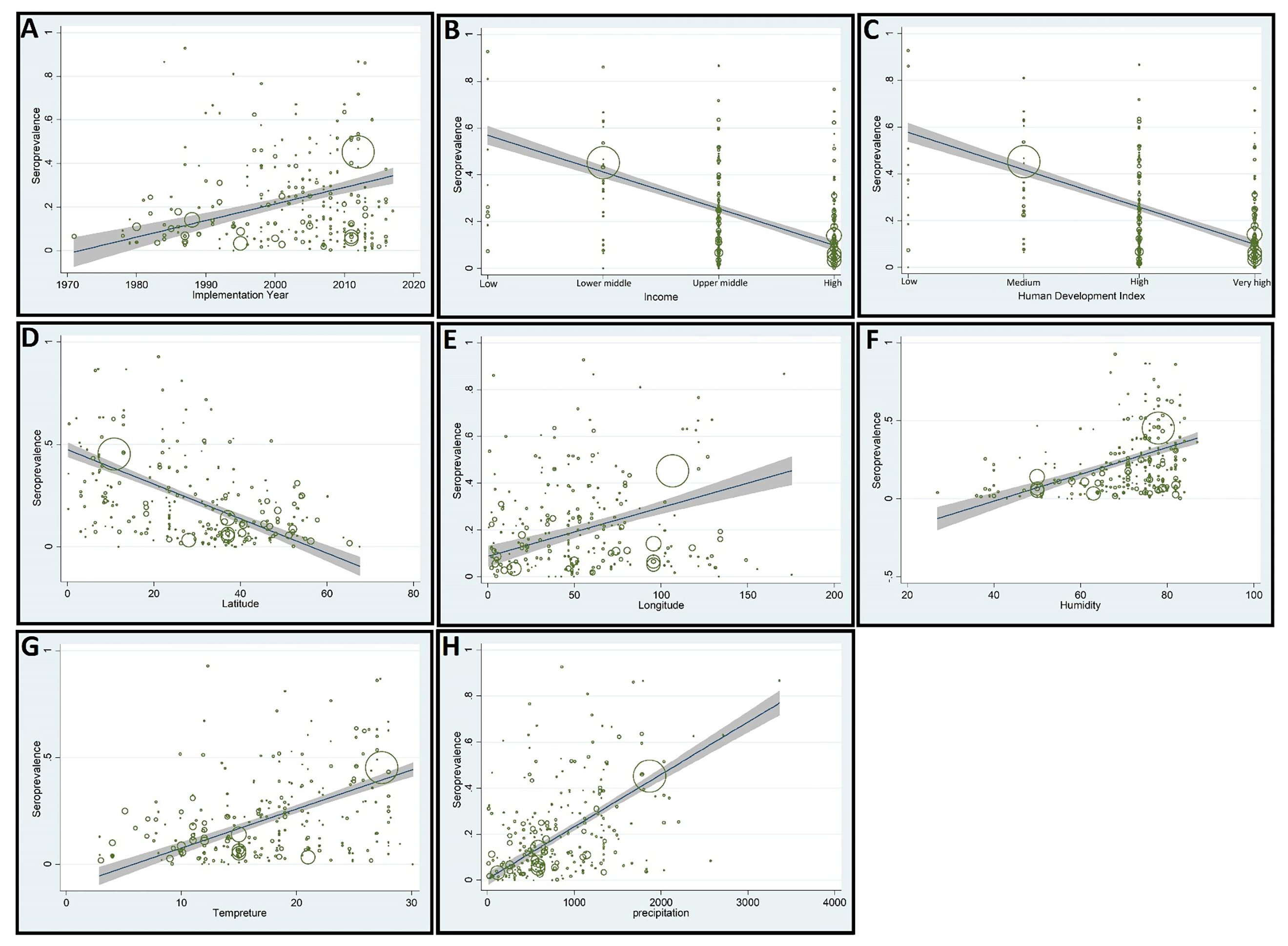

Supplement: S3 Fig — Ecological linear meta-regression analyses of the global T-seroprevalence: (panel A) implementation years of screening showing a non-statistically significant upward trend in seroprevalence in more recent years (C = 0.0007, P-value = 0.57); (panel B) country’s income level showing a showing a statistically significant downward trend in seroprevalence in countries with higher level of income (C = -0.082, P-value < 0.001); (panel C) human development index (HDI) showing a statistically significant downward trend in seroprevalence in countries with higher levels of HDI (C = -0.084, P-value < 0.001); (panel D) geographical latitude a showing a statistically significant downward trend in seroprevalence with increasing geographical latitude (C = -0.005, P-value < 0.001); (panel E) geographical longitude a showing a non-statistically significant upward trend in seroprevalence with increasing geographical longitude (C = 0.0005, P-value = 0.1); (panel F) the relative humidity showing a statistically significant upward trend in seroprevalence in areas with higher relative humidity (C = 0.005, P-value < 0.001); (panel G) the mean temperature showing a statistically significant upward trend in seroprevalence with increasing mean temperature (C = 0.01, P-value < 0.001); (panel H) the annual precipitation showing a statistically significant upward trend in seroprevalence with increasing rate of precipitation (C = 0.0001, P-value < 0.001). C = coefficient. (TIF) [file pntd.0007809.s007.tif]
