# Supplementary material for: Seroprevalence estimates for toxocariasis in people worldwide: A systematic review and meta-analysis
Source: PLoS Negl Trop Dis. 2019 Dec 19;13(12):e0007809. doi: 10.1371/journal.pntd.0007809 (PMC6922318; doi:10.1371/journal.pntd.0007809)
Supplement: S1 Text — (DOCX) [file pntd.0007809.s008.docx]

**S1 Text.** Details of the databases searches

**EMBASE=6120**

1. (('(Toxocara infection':ab,ti OR 'toxocariasis':ab,ti OR 'toxocara spp.)':ab,ti) AND '(prevalence':ab,ti OR 'seroprevalence':ab,ti OR 'seroepidemiology)':ab,ti) AND '(human)':ab,ti AND [1980-2019]/py = **4995**

2. ('Toxocara infection'/exp OR 'toxocara infection' OR (('toxocara'/exp OR toxocara) AND ('infection'/exp OR infection)) OR 'toxocariasis'/exp OR toxocariasis OR 'toxocara spp.' OR (('toxocara'/exp OR toxocara) AND spp.) OR 'toxocara canis'/exp OR 'toxocara canis' OR (('toxocara'/exp OR toxocara) AND ('canis'/exp OR canis)) OR 'toxocara cati'/exp OR 'toxocara cati' OR (('toxocara'/exp OR toxocara) AND cati)) AND ('prevalence'/exp OR prevalence OR 'seroprevalence'/exp OR seroprevalence OR 'seroepidemiology'/exp OR seroepidemiology) AND [1980-2019]/py = **1125**

**Web of sciences=2261**

**You searched for: TOPIC:** ((Toxocara infection OR Toxocariasis OR Toxocara spp.) AND (prevalence OR seroprevalence OR seroepidemiology)) = **913**

Timespan: All years. Indexes: SCI-EXPANDED, SSCI, CPCI-S, CPCI-SSH, ESCI.

**You searched for: TOPIC:** ((Toxocara infection OR Toxocariasis OR Toxocara spp.) AND (prevalence OR seroprevalence OR seroepidemiology) AND (human)) = **361**

Timespan: All years. Indexes: SCI-EXPANDED, SSCI, CPCI-S, CPCI-SSH, ESCI.

**You searched for: TOPIC:** ((Toxocara infection OR Toxocariasis OR Toxocara spp. OR toxocara canis OR toxocara cati) AND (prevalence OR seroprevalence OR seroepidemiology)) = **987**

Timespan: All years. Indexes: SCI-EXPANDED, SSCI, CPCI-S, CPCI-SSH, ESCI.

**Scopus: 1783**

1. TITLE-ABS-1. TITLE-ABS-TITLE-ABS-KEY (("Toxocara infection" OR "toxocariasis" OR "toxocara spp.") AND ("prevalence" OR "seroprevalence" OR "seroepidemiology")) = **773**

2. TITLE-ABS KEY ((Toxocara AND infection OR toxocariasis OR toxocara AND spp.) AND (prevalence OR seroprevalence OR seroepidemiology) AND (human)) = **232**

3. TITLE-ABS-KEY ((toxocara AND infection OR toxocariasis OR toxocara AND spp.) AND (prevalence OR seroprevalence OR  seroepidemiology) AND (human) AND (risk AND factor)) = **75**

**SCIELO=52**

(Toxocara infection OR Toxocariasis OR Toxocara spp.) AND (prevalence OR seroprevalence OR seroepidemiology) = **37**

(Toxocara infection OR Toxocariasis OR Toxocara spp.) AND (prevalence OR seroprevalence OR seroepidemiology) AND (human) = **15**

**PubMed= 2688**

1. (("Toxocariasis"[MeSH Terms] OR "toxocariasis"[All Fields] OR ("toxocara"[All Fields] AND "infection"[All Fields]) OR "toxocara infection"[All Fields]) OR ("toxocariasis"[MeSH Terms] OR "toxocariasis"[All Fields]) OR (("toxocara"[MeSH Terms] OR "toxocara"[All Fields]) AND ("Sci Public Policy"[Journal] OR "spp"[All Fields]))) AND (("epidemiology"[Subheading] OR "epidemiology"[All Fields] OR "prevalence"[All Fields] OR "prevalence"[MeSH Terms]) OR ("seroepidemiologic studies"[MeSH Terms] OR ("seroepidemiologic"[All Fields] AND "studies"[All Fields]) OR "seroepidemiologic studies"[All Fields] OR "seroprevalence"[All Fields]) OR seroepidemiology[All Fields]) AND ("1980/01/01"[PDAT] : "2019/03/15"[PDAT]) = **1117**

2. (("Toxocariasis"[MeSH Terms] OR "toxocariasis"[All Fields] OR ("toxocara"[All Fields] AND "infection"[All Fields]) OR "toxocara infection"[All Fields]) OR ("toxocariasis"[MeSH Terms] OR "toxocariasis"[All Fields]) OR (("toxocara"[MeSH Terms] OR "toxocara"[All Fields]) AND ("Sci Public Policy"[Journal] OR "spp"[All Fields]))) AND (("epidemiology"[Subheading] OR "epidemiology"[All Fields] OR "prevalence"[All Fields] OR "prevalence"[MeSH Terms]) OR ("seroepidemiologic studies"[MeSH Terms] OR ("seroepidemiologic"[All Fields] AND "studies"[All Fields]) OR "seroepidemiologic studies"[All Fields] OR "seroprevalence"[All Fields]) OR seroepidemiology[All Fields]) AND ("humans"[MeSH Terms] OR "humans"[All Fields] OR "human"[All Fields]) AND ("1980/01/01"[PDAT] : "2019/03/15"[PDAT]) = **802**

3. (("Toxocariasis"[MeSH Terms] OR "toxocariasis"[All Fields] OR ("toxocara"[All Fields] AND "infection"[All Fields]) OR "toxocara infection"[All Fields]) OR ("toxocariasis"[MeSH Terms] OR "toxocariasis"[All Fields]) OR (("toxocara"[MeSH Terms] OR "toxocara"[All Fields]) AND ("Sci Public Policy"[Journal] OR "spp"[All Fields]))) AND (("epidemiology"[Subheading] OR "epidemiology"[All Fields] OR "prevalence"[All Fields] OR "prevalence"[MeSH Terms]) OR ("seroepidemiologic studies"[MeSH Terms] OR ("seroepidemiologic"[All Fields] AND "studies"[All Fields]) OR "seroepidemiologic studies"[All Fields] OR "seroprevalence"[All Fields]) OR seroepidemiology[All Fields]) AND ("humans"[MeSH Terms] OR "humans"[All Fields] OR "human"[All Fields]) AND ("risk factors"[MeSH Terms] OR ("risk"[All Fields] AND "factors"[All Fields]) OR "risk factors"[All Fields] OR ("risk"[All Fields] AND "factor"[All Fields]) OR "risk factor"[All Fields]) AND ("1980/01/01"[PDAT] : "2019/03/15"[PDAT]) = **229**
